# Ibuprofen Degradation and Associated Bacterial Communities in Hyporheic Zone Sediments

**DOI:** 10.3390/microorganisms8081245

**Published:** 2020-08-16

**Authors:** Cyrus Rutere, Kirsten Knoop, Malte Posselt, Adrian Ho, Marcus A. Horn

**Affiliations:** 1 Department of Ecological Microbiology, University of Bayreuth, 95448 Bayreuth, Germany; cyrus.njeru@uni-bayreuth.de; 2 Institute of Microbiology, Leibniz University Hannover, 30419 Hannover, Germany; kirsten-knoop@gmx.de (K.K.); adrian.ho@ifmb.uni-hannover.de (A.H.); 3 Department of Environmental Science, Stockholm University, SE-106 91 Stockholm, Sweden; malte.posselt@aces.su.se

**Keywords:** ibuprofen, hyporheic zone, sediments, micropollutant, biodegradation, model organism, microbial ecology

## Abstract

Ibuprofen, a non-steroidal anti-inflammatory pain reliever, is among pharmaceutical residues of environmental concern ubiquitously detected in wastewater effluents and receiving rivers. Thus, ibuprofen removal potentials and associated bacteria in the hyporheic zone sediments of an impacted river were investigated. Microbially mediated ibuprofen degradation was determined in oxic sediment microcosms amended with ibuprofen (5, 40, 200, and 400 µM), or ibuprofen and acetate, relative to an un-amended control. Ibuprofen was removed by the original sediment microbial community as well as in ibuprofen-enrichments obtained by re-feeding of ibuprofen. Here, 1-, 2-, 3-hydroxy- and carboxy-ibuprofen were the primary transformation products. Quantitative real-time PCR analysis revealed a significantly higher 16S rRNA abundance in ibuprofen-amended relative to un-amended incubations. Time-resolved microbial community dynamics evaluated by 16S rRNA gene and 16S rRNA analyses revealed many new ibuprofen responsive taxa of the Acidobacteria, Actinobacteria, Bacteroidetes, Gemmatimonadetes, Latescibacteria, and Proteobacteria. Two ibuprofen-degrading strains belonging to the genera *Novosphingobium* and *Pseudomonas* were isolated from the ibuprofen-enriched sediments, consuming 400 and 300 µM ibuprofen within three and eight days, respectively. The collective results indicated that the hyporheic zone sediments sustain an efficient biotic (micro-)pollutant degradation potential, and hitherto unknown microbial diversity associated with such (micro)pollutant removal.

## 1. Introduction

Ibuprofen is a non-steroidal anti-inflammatory drug widely consumed globally for its analgesic, anti-inflammatory, and antipyretic properties. Its removal in most wastewater treatment plants (WWTPs) is relatively efficient (about 90%) [1]. However, low to trace concentrations of the pharmaceutical continue to be detected in the aquatic environment. This may be attributed to the continuous input of the compound via raw sewage coupled with a short retention time in the WWTPs, resulting in pseudo-persistence [2]. Toxic effects of ibuprofen in the environment have been documented. For example, ibuprofen alongside other pharmaceutical residues reduced the bacterial biomass of some riverine biofilm communities [3]. Cytotoxic effects of ibuprofen combined with other pharmaceuticals on human kidney embryonic cells have also been reported [4]. Ibuprofen was detected in the bile of wild fish caught downstream of a WWTP [5] in addition to the adverse impact on the reproduction of some aquatic organisms, such as zebrafish, planktonic crustaceans, and the Japanese rice fish [6]. Such findings give rise to concerns on the long-term ecological impact of the compound on aquatic ecosystems.

Ibuprofen-containing effluents are usually part of the surface water volume exchanged between the open channel and the permeable streambed sediments (hyporheic zone) of impacted rivers. These hyporheic zones are thought to play a critical role in the cycling of nutrients and degradation of pollutants as they are characterized by the active exchange of pore water, nutrients, and biota between surface water and aquifers [7]. The large surface area occupied by sediment particles, pore spaces, organic matter, and the prevalence of redox gradients along the sediment profile represent an ideal habitat for the proliferation of a diverse microbial community with high metabolic versatility [8]. Though biodegradation is considered the primary attenuation mechanism for ibuprofen in both constructed and natural environments, the bacteria associated with this degradation in the streambed sediments are virtually unknown [9,10,11,12].

To date, only several bacterial strains, *Bacillus thuringiensis* B1 (2015b), *Sphingomonas Ibu-2, Variovorax Ibu-1*, *Sphingobium yanoikuyae*, and *Pseudoxanthomonas* sp. DIN-3, have been shown to utilize ibuprofen as the sole source of carbon and energy, with oxygen as the terminal electron acceptor [13,14,15,16,17]. Transformation products, such as ibuprofen carboxylic acid (CBX IBU), 2-hydroxyibuprofen (2-OH IBU), 1-hydroxyibuprofen (1-OH IBU), and 1,2-dihydroxy ibuprofen detected in batch experiments with activated sludge, biofilm reactors, and *Bacillus thuringiensis* B1 [9,15,18], suggest diverse biodegradation pathways, likely driven by diverse microbial communities. In the sediment, a high diversity of the resident bacterial community and a rather long contact time in the sediment matrix [19,20,21] suggest an ideal microhabitat with an enhanced ibuprofen biodegradation potential relative to WWTPs.

The projected population growth and rapid urbanization will likely result in increased consumption of ibuprofen and associated release into the aquatic ecosystems. Such contamination of receiving rivers that are a significant source of drinking water globally may result in dire consequences to human and overall ecosystem wellbeing. Understanding the impact of ibuprofen on the indigenous microbial community composition and diversity would contribute to understanding the dynamics regulating ibuprofen removal in the hyporheic zone. However, the current knowledge of ibuprofen degradation-associated genes or metabolic pathways is limited. Thus, functional gene-based approaches are inadequate to date in order to target ibuprofen degraders, and new model organisms for their development are warranted. Identification of ibuprofen-responsive taxa based on analysis of their 16S rRNA gene, and isolation is a promising strategy to identify hitherto unknown candidates for key bacterial taxa with ibuprofen degradation capabilities.

Based on the information and knowledge gaps highlighted, we hypothesize that hyporheic zone sediments are a reservoir of diverse hitherto unknown microbes that are prone to quickly respond to ibuprofen, thus sustaining a high ibuprofen degradation potential. Our objectives were to (i) determine ibuprofen biodegradation potentials in the hyporheic zone sediment downstream from a WWTP using oxic microcosm incubations, (ii) identify degradation intermediates and hypothetical degradation pathways, (iii) relate microbial community changes to ibuprofen exposure, and hence to (iv) identify potential ibuprofen degraders, including the provision of new model ibuprofen degraders.

## 2. Materials and Methods

### 2.1. Study Site and Sampling

The River Erpe is a lowland stream located east of Berlin, Germany. It is a receiving river, highly impacted by the municipal wastewater from the WWTP Münchehofe that accounts for 60–80% of its total discharge. The sampling site is located approximately 0.7 km downstream of the effluent discharge site at Heidemühle (latitude, 52.478647; longitude, 13.635146). The site is characterized by fine-sandy siliceous sediment as determined in a previous field study [19]. Sediment and water samples were collected from the site in September 2015. Surface sediment samples (0–5 cm depth) were collected from several spots on the streambed with a flat hand shovel and stored in sterile Whirl-Pak sampling bags (Merck, Darmstadt, Germany). Surface water samples were obtained from the same site and stored in sterile 2-L screw-mouth Duran^®^ bottles (Merck, Darmstadt, Germany). Freshly collected sediment and water samples were transferred to the laboratory at 4 °C, manually homogenized, and an aliquot stored at −80 °C for subsequent nucleic acid extraction. The remaining fresh sediment was stored at 4 °C and used to set up microcosm incubation experiments within two weeks.

### 2.2. Chemicals

Analytical-grade ibuprofen, 1-, 2-, 3-hydroxyibuprofen, carboxy-ibuprofen, and sodium acetate trihydrate were purchased from Sigma-Aldrich (Steinheim, Germany). HPLC-grade acetonitrile and H_2_SO_4_ was purchased from Merck (Darmstadt, Germany). Isotopically labelled internal standards (purity >98%), were purchased from Toronto Research Chemicals Inc. (North York, ON, Canada). Stock solutions of ibuprofen (1 mM) and 10 mM acetate were prepared separately in distilled water and filter sterilized (0.2 µm pore diameter), whereas internal standards were prepared in methanol.

### 2.3. Microcosm Setup

The microcosms were prepared in triplicates using 25 g of fresh sediment and 50 mL of natural river water in autoclaved 120-mL conical flasks. A 10-day oxic pre-incubation at 15 °C to reduce endogenous dissolved organic carbon was performed. Subsequently, one set of microcosms was amended with ibuprofen to achieve final concentrations of 5, 40, 200, and 400 µM. The second set was amended with the same ibuprofen concentrations and 1 mM acetate to test for the cometabolic degradation potential of ibuprofen. An unamended control setup consisting of microcosms without ibuprofen and acetate and two abiotic controls comprising of autoclave-sterilized sediment (i.e., sorption control), and autoclave-sterilized river water (i.e., hydrolysis control) amended with a final concentration of 200 µM ibuprofen were included. All flasks were sealed with sterilized Steristoppers^®^ (Heinz Herenz, Hamburg, Germany). Oxic microcosms were then incubated in the dark at 15 °C with shaking at 100 rotations per minute. Liquid samples were taken under sterile conditions after the first amendment (t0) and at regular intervals following incubation and the concentration of ibuprofen and acetate determined using the high-performance liquid chromatography (HPLC) (see below). Ibuprofen or ibuprofen-acetate were refed four more times to the same initial concentrations upon complete substrate depletion (i.e., five amendments in total). Following the third refeeding and at the end of the incubation (after the fifth refeeding), 2 g of sediment subsamples were taken from the treatments and the unamended controls and stored at −80°C for subsequent nucleic acid extraction.

### 2.4. Chemical Analyses

The concentration of test compounds was determined from centrifuged slurry samples (13,000× *g*, 5 min). The supernatant was microfiltered (0.22 µm pore diameter, PTFE membrane) and quantification performed with HPLC. Ibuprofen was quantified using an Agilent 1260 series HPLC fitted with a Zorbax SB-C18 column at 30 °C and a diode array detector (Agilent 1260 series, Agilent Technologies, Santa Clara, CA, USA) under isocratic conditions. The mobile phase was acetonitrile-20 mM acetate buffer (50:50 *v/v*), pH 3 at a flow rate of 0.5 mL min^-1^. Spectra ranging from 200 to 320 nm were used to determine peak purity. The absorbance signal at 225 nm was used for quantification with external standards prepared in deionized water.

Aliquots of the samples obtained from the 200 µM ibuprofen treatment after the fifth refeeding were analyzed for ibuprofen transformation intermediates using a newly developed and validated direct injection method utilizing ultra-high-performance liquid chromatography-tandem mass spectrometry (UHPLC-MS/MS; [22]). Briefly, separations were carried out on a Thermo Scientific (Waltham, MA, USA) Dionex Ultimate 3000 UHPLC system equipped with a Waters (Manchester, UK) ACQUITY UPLC HSS T3 column (1.8 µm, 2.1 mm × 100 mm). A mobile phase gradient of 97% deionized water (+10 mM acetic acid, A) and 97% methanol (+10 mM acetic acid, B) was used. The mobile phase gradient was ramped from 97% A to 97% B in 16 min to achieve separation of 2-OH ibuprofen and 3-OH ibuprofen. For MS/MS analysis, a Thermo Scientific Quantiva triple-stage quadrupole mass spectrometer with an H-ESI interface was operated in negative selected reaction monitoring mode. MS data were processed using ‘Xcalibur 3.1.66.10′ (Thermo Scientific, Waltham, MA, USA). Precision was excellent for all compounds with <5% relative standard deviation of a quality control standard measured every 10 samples.

The acetate concentration was measured using HPLC (1090 series II, Hewlett Packard, Palo Alto, USA) equipped with a variable wavelength UV detector (Series 1200, Agilent Technologies, Böblingen, Germany) at an absorption wavelength of 210 nm. Liquid samples (10 µL) were injected into the mobile phase (4 mM H_3_PO_4_, pH 2.5) via an autosampler and the sample-mobile phase mixture was pumped through an ion exclusion column (Rezex ROA Organic Acid H+ column, 300 × 7.8 mm, Phenomenex, Torrance, CA, USA) with a flow rate of 0.8 mL min^−1^. The column was heated to 60 °C. The concentration was calculated based on the peak area from known concentrations of six to eight external standards.

### 2.5. Nucleic Acids Extraction, Quantification, and Reverse Transcription

Nucleic acids were extracted from each of the three independent replicates per treatment following a published protocol [23]. DNA and RNA were obtained from the coextracts using DNase-free RNase and RNase-free DNase (Promega, Mannheim, Germany), respectively, following the manufacturer’s instructions. RNA samples were confirmed to be DNA free after a control PCR targeting the 16S rRNA gene (primers 27F and 907R; [24] according to [25]), showing no amplification. DNA and RNA concentrations were determined with Quant-iT^®^ PicoGreen DNA and RiboGreen RNA assay kits (Invitrogen, Karlsruhe, Germany), respectively, on a Multiskan™ FC Microplate Photometer (Thermo Fischer Scientific, Darmstadt, Germany). Reverse transcription of RNA into complementary DNA (cDNA) was performed using random hexamer primers and Superscript™ III Reverse Transcriptase (Invitrogen, Karlsruhe, Germany) following [26].

### 2.6. Amplicon Sequencing

Amplification and sequencing of the bacterial 16S rRNA genes and 16S rRNA were performed at LGC Genomics GmbH (Berlin, Germany). Briefly, the PCR consisted of 1 × MyTaq buffer containing 1.5 units MyTaq DNA polymerase (Bioline, UK) and 2 µL of BioStabII PCR Enhancer (Sigma-Aldrich, Darmstadt, Germany), 15 pmol of each forward primer U341F and reverse primer U806R [27], and 5 ng of DNA/cDNA per sample in nuclease-free water (Thermo Fischer Scientific, Darmstadt, Germany) in a final 20-µL volume (protocol kindly provided by LGC). For each sample, the forward and reverse primers had the same 10-nt barcode sequence. The PCR was carried out using the following thermal profile: 2 min at 96 °C initial denaturation followed by 30 cycles of 96 °C for 15 s, 50 °C for 30 s, and elongation at 70 °C for 90 s. Amplification was confirmed by gel electrophoresis on 1% agarose gels. About 20 ng of amplicon DNA of each sample were pooled for up to 48 samples with different barcodes. If needed, PCRs showing low yields were further amplified for five cycles. The amplicon pools were purified with one volume AMPure XP beads (Beckman Coulter, Brea, CA, USA), to remove primer dimers, followed by an additional purification on MinElute columns (Qiagen, Hilden, Germany). About 100 ng of each purified amplicon pool DNA were used to construct Illumina sequencing libraries using the Ovation Rapid DR Multiplex System 1-96 (NuGEN, Leek, The Netherlands). Illumina libraries were pooled, and the size selected by preparative gel electrophoresis. Sequencing was performed on an Illumina MiSeq platform using V3 Chemistry (Illumina, San Diego, CA, USA) yielding 300-base paired-end reads.

#### Processing of Amplicon Sequence Data

Raw 16S rRNA gene and 16S rRNA sequences were pre-processed by demultiplexing all libraries using the Illumina bcl2fastq 1.8.4 software. The reads were then sorted by amplicon inline barcodes corresponding to independent samples. The barcode sequences were clipped from sequences after sorting, followed by clipping of the sequencing adapter remnants from all reads. Reads with a final length <100 bases were discarded. Primer sequences were removed, the sequence fragments turned into forward-reverse orientation, and merged using BBMerge 34.38. 16S pre-processing and operational taxonomic unit (OTU) picking from amplicons was performed with Mothur 1.35.1 [28]. Briefly, sequences containing ambiguous bases (Ns), with homopolymer stretches of more than eight bases or with an average Phred quality score below 33 were removed. Remaining sequences were aligned against the 16S Mothur-Silva SEED r119 reference alignment. Sequencing error reduction was achieved through preclustering, and elimination of chimera carried out with UCHIME [29]. This was followed by taxonomical classification of the sequences (against the Silva reference classification) and removal of sequences from domains of life other than Bacteria and Archaea. The resultant number of 16S rRNA gene and 16S rRNA sequences per sample ranged from 10,767 to 19,244 and 11,616 to 19,397, respectively. Single replicates with less than 1000 sequence reads were excluded from further analysis. OTU picking by clustering at the 97% identity level (using the cluster.split method) and OTU consensus taxonomical calling integrating the taxonomical classification of the cluster member sequences was then performed. The representative sequences of each OTU (with at least two observed sequences) were queried against a filtered (unknown and unclassified sequences were removed) version of the ribosomal database project release 11.4 reference. A summary table with the taxonomy and alignment details for each OTU representative sequence was generated. The OTU abundance table was variance filtered and normalized (total sum scaling resulting in relative abundances) according to default settings in the Microbiome Analyst pipeline unless indicated otherwise [30]. Datasets were rarefied to a uniform sequencing depth of 10,767 sequences per sample for comparative analyses except for DESeq2 analyses, where the non-rarefied non-normalized datasets were used according to [31]. Please note that Silva r119 used in the current study classifies the “Betaproteobacteria” as “Betaproteobacteriales”, an order of the Gammaproteobacteria. Thus, genera and higher taxonomic ranks that formerly represented “Betaproteobacteria” now belong to “Gammaprotobacteria”.

### 2.7. Primer Design and Quantification of Total and Taxon-Specific 16S rRNA and 16S rRNA Genes

Quantitative kinetic PCR (qPCR) was used to estimate the numerical abundance of 16S rRNA gene and 16S rRNA sequences of the total bacterial community as well as of some representative OTUs. The representative OTUs were chosen for their significantly high differential relative abundance in the 400 µM ibuprofen treatment amplicon libraries relative to unamended controls as determined by the DeSeq2 approach (see Figure 3 and Figure S7 and Table S2). To quantify these OTUs, specific primers were designed in Primer-Blast using the default settings [32]. One primer set targeting an unclassified OTU affiliated to the class Actinobacteria was obtained from [33] (Table S1). All primers were tested in silico against target and non-target sequences to ascertain specificity, with verified primers subsequently synthesized at Microsynth Seqlab (Göttingen, Germany). The fidelity of the synthesized primers against DNA samples was assessed using PCR and subsequent cloning [34]. The cloned amplicons were subjected to Sanger sequencing and checked against the NCBI nucleotide database, confirming correct amplification and classification of the targeted taxa. Sequences with a 99% similarity to the targeted taxa were used to generate qPCR standards as previously described [35].

For both total community and specific OTU amplification, DNA and cDNA samples were diluted 100- and 50-fold, respectively, to reduce potential inhibition of qPCR by coextracted PCR-inhibiting compounds. Spiking assays demonstrated inhibition-free amplifications at such dilutions [35]. Five microliters of DNA or cDNA were used as templates in 20-µL reaction mixtures containing 10 μL of SensiMix Plus with SYBR Green and Fluorescein (Bioline GmbH, Luckenwalde, Germany), 1.2 µL of a 50 mM MgCl_2_ solution, 150 ng/µL of bovine serum albumin, 0.2–1.6 pM of each primer (Biomers, Ulm, Germany), and nuclease-free water (Thermo Fischer Scientific, Darmstadt, Germany). Each sample was assayed in technical quadruplicates to determine copy numbers with external standards generated from the amplified clones using a Bio-Rad iQ5 optical system software version 2.0. (Bio-Rad Laboratories Inc, Hercules, CA, USA). The total bacterial community was amplified according to [35]. Briefly, the primer set 341F/534R, and the following program was used: Initial denaturation at 95 °C for 10 min, and 35 cycles of denaturation at 94 °C for 30 s, primer annealing at 55.7  °C for 40 s, and elongation at 72 °C for 40 s. The final elongation was at 72 °C for 5 min. The program for amplification of specific OTUs was initial denaturation at 95 °C for 3 min, and 35 cycles of denaturation at 94 °C for 30 s, primer annealing at the respective temperatures shown in Table S1 for 30 s, and elongation at 72 °C for 90 s. The final elongation was at 72 °C for 7 min.

### 2.8. Statistical Analyses

Alpha diversity was assessed based on Shannon indices and richness estimators using rarefied sequence data applying post-hoc Tukey test using PAST v3.15 [36]. Differences in bacterial community compositions across treatments were visually assessed with principal coordinate analysis (PCoA) and analysis of similarity (ANOSIM) applying the Bray–Curtis distance metric. The linear discriminant analysis (LDA) effect size (LEfSe) algorithm [37], as implemented in the Microbiome Analyst pipeline [30], was used to identify specific taxa whose relative abundance changed significantly over time. All samples were compared against each other, and significant taxa were ranked according to the difference in their mean relative abundances from all ibuprofen-supplemented samples and all unamended samples, including t0. The LEfSe analysis was based on three replicates, and a Kruskal–Wallis test-derived *p*-value of <0.05 was used to indicate a statistically significant difference. Potential ibuprofen degraders were identified based on the significant differential abundance values (*p*-adj <0.05) between unamended controls and amended treatments using the DESeq2 package [31] in R (3.5.2) [38]. Genera with a significant Log2FoldChange >0 in ibuprofen treatments relative to unamended controls were considered enriched. ANOVA and Tukey tests were applied to evaluate the effect of treatments on total bacteria and OTU-specific 16S rRNA and rRNA gene abundance.

### 2.9. Enrichment and Isolation of Ibuprofen-Degrading Bacteria from Hyporheic Zone Sediments

**Figure 1:**
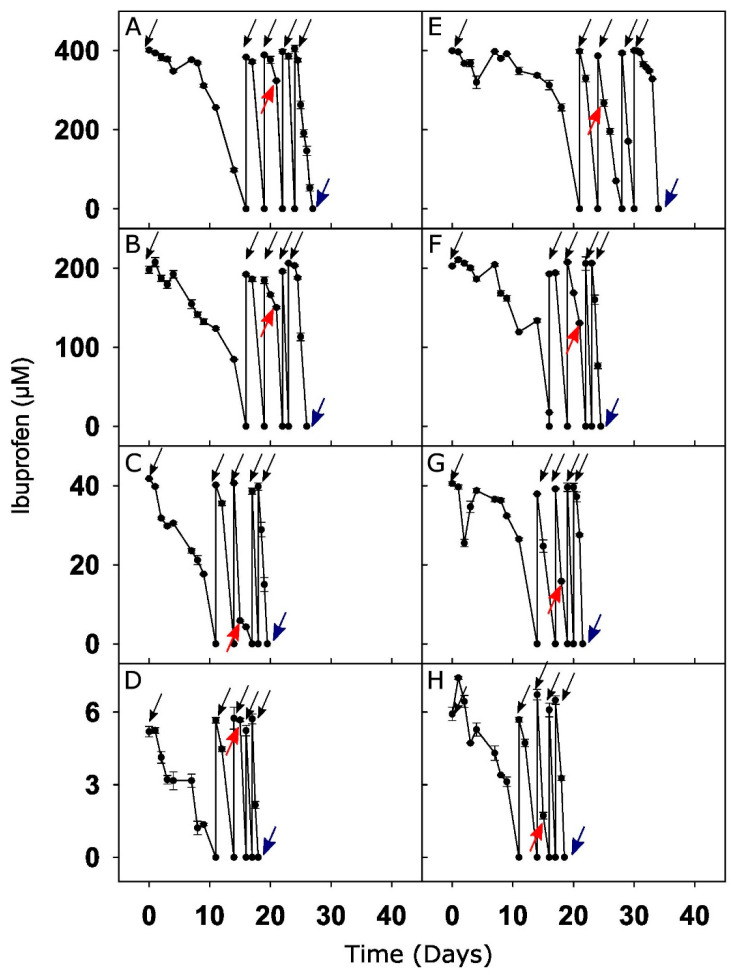
Degradation of ibuprofen in oxic hyporheic zone sediment microcosms. Plots (**A**–**D**) correspond to sediment amended with ibuprofen concentrations of 5, 40, 200, and 400 μM, respectively. Plots (**E**–**H**) correspond to sediment amended with both 1 mM acetate and ibuprofen concentrations of 5, 40, 200, and 400 μM, respectively. Values are the arithmetic means of triplicate oxic incubations. Error bars indicate standard deviations. Some standard deviations are smaller than the symbol size and therefore not apparent. Arrows indicate the time of refeeding of microcosms with ibuprofen (**A**–**D**) and acetate and ibuprofen (**E**–**H**), respectively. Red and blue arrows indicate sampling of the sediment for nucleic acid extraction after the third and fifth refeeding, respectively.

The microcosms, amended exclusively with 400 µM ibuprofen after 27 days of incubation (Figure 1), were pooled and a subsample of sediment was obtained and subjected to a serial dilution (1:10 (*w/v*)) in modified oxic mineral salts medium [39]. The medium contained (in mg L^−1^) mineral salts (NaCl, 100; (NH_4_)_2_SO_4_, 25; CaCl_2_ 2H_2_O, 10; MgCl_2_·6H_2_O, 10; NH_4_Cl, 50; KH_2_PO_4_, 50), trace elements (C_6_H_6_NNa_3_O_6_·H_2_O, 15; MnSO_4_·H_2_O, 5; FeSO_4_·7H_2_O, 1; CoCl_2_·6H_2_O, 1; CaCl_2_·2H_2_O, 1; ZnSO_4_·7H_2_O, 1; AlK(SO_4_)_2_·12H_2_O, 0.2; CuSO_4_·5H_2_O, 0.1; H_3_BO_3_, 0.1; Na_2_MoO_4_·2H_2_O, 0.1), and 10 mL L^−1^ vitamin stock solution [40]. Each dilution was supplemented with 400 µM ibuprofen as the only-carbon and energy source. Sediment dilutions were incubated for 21 days at 15°C in the dark and ibuprofen degraders further enriched. The 10^−6^ dilution was the highest sediment dilution showing turbidity indicative of growth and was streak-plated onto the same medium solidified with 1% agar. Six isolated colonies were then transferred to the oxic liquid mineral salts medium containing 400 µM ibuprofen and tested for ibuprofen-degrading capabilities using HPLC. Two ibuprofen-degradation-positive colonies were purified via repeated streak-plating, picking of single colonies, and transfer to liquid media supplemented with ibuprofen. Cell morphology was examined with a Zeiss AxioPlan Fluorescence Phase Contrast Microscope (ZEISS, Oberkochen, Germany). The homogeneous colony and cell morphologies indicated the purity of isolates. At least five colonies from each isolate were subjected to colony PCR, and the 16S rRNA gene was amplified from genomic DNA using the primer set 27F/1492R [41,42] and the following program: Initial denaturation at 94 °C for 10 min, and 30 cycles of denaturation at 94 °C for 30 s, primer annealing at 52 °C for 40 s, and elongation at 72 °C for 90 s. The final elongation was at 72 °C for 7 min. Sanger-sequencing reactions were performed at Microsynth Seqlab (Göttingen, Germany). 16S rRNA gene sequences from all respective isolate colonies were compared using the NCBI blast search tool with the GenBank database. The similarity between the five or more 16S rRNA gene sequences from different colonies of one isolate was higher than 99%. Nearly complete 16S rRNA gene sequences of 1444 and 1380 bases for the two isolates, i.e., strains CN1 and MAH1, were obtained. These 16S rRNA gene sequences were aligned with closely cultured relatives as indicated by blastn analysis against GeneBank using the ARB-SILVA aligner tool (www.arb-silva.de/aligner). Phylogenetic tree reconstruction was performed using the Molecular Evolutionary Genetic Analysis (MEGA v7) tool with the neighbor-joining method and bootstrap analysis [43]. Following the identification of these strains, detailed characterization of the ibuprofen degraders will be performed in the future.

#### Accession Numbers

Illumina sequence data were deposited in the NCBI Sequence Read Archive under the accession number PRJNA529686. Sequences of qPCR standards generated in this study were deposited in the GenBank nucleotide sequence database under the accession numbers MK732962-MK732969. 16S rRNA gene sequences of *Novosphingobium* sp. strain CN1 and *Pseudomonas thivervalensis* strain MAH1 were deposited in the GenBank nucleotide sequence database under the accession numbers MK910996 and MN317372, respectively.

## 3. Results

### 3.1. Transformation of Ibuprofen in Hyporheic Zone Sediments

Degradation of ibuprofen in ibuprofen-amended sediment microcosms occurred without an appreciable delay except for the 400 µM treatment, which exhibited an initial nine-day lag phase (Figure 1A–D). Ibuprofen was depleted within 11 days for the 5 and 40 µM treatments, and within 16 days for the 200 and 400 µM treatments. In the presence of acetate as a primary carbon source, the degradation of ibuprofen tended to be delayed across most concentrations after the first amendment with ibuprofen and acetate (Figure 1E–H). The initial time needed for the depletion of ibuprofen depended on the initial ibuprofen concentration as well as acetate supplementation and ranged from 11–34 days (Figure 1). After subsequent refeedings, ibuprofen was degraded entirely within 1–3 days in the presence and absence of acetate, indicating the enrichment of ibuprofen degraders. Ibuprofen concentrations were essentially constant in control microcosms containing autoclaved sediment and river water, while ibuprofen was below the detection limit in the unamended microcosms (Figure S1). Microcosms amended with 200 µM ibuprofen were chosen for in-depth transformation product analysis as representative treatments with a high initial ibuprofen concentration as well as a quick ibuprofen degradation after the fourth refeeding. 1-hydroxyibuprofen, 2-hydroxyibuprofen, 3-hydroxyibuprofen, and carboxyibuprofen were transiently detected in concentrations accounting for less than 1% of the initially supplied ibuprofen, suggesting that such compounds were transformation intermediates (Figure S2).

### 3.2. Effect of Treatments on Bacterial Community Structure and Composition

#### 3.2.1. Total Bacterial Abundance and Diversity

**Figure 2:**
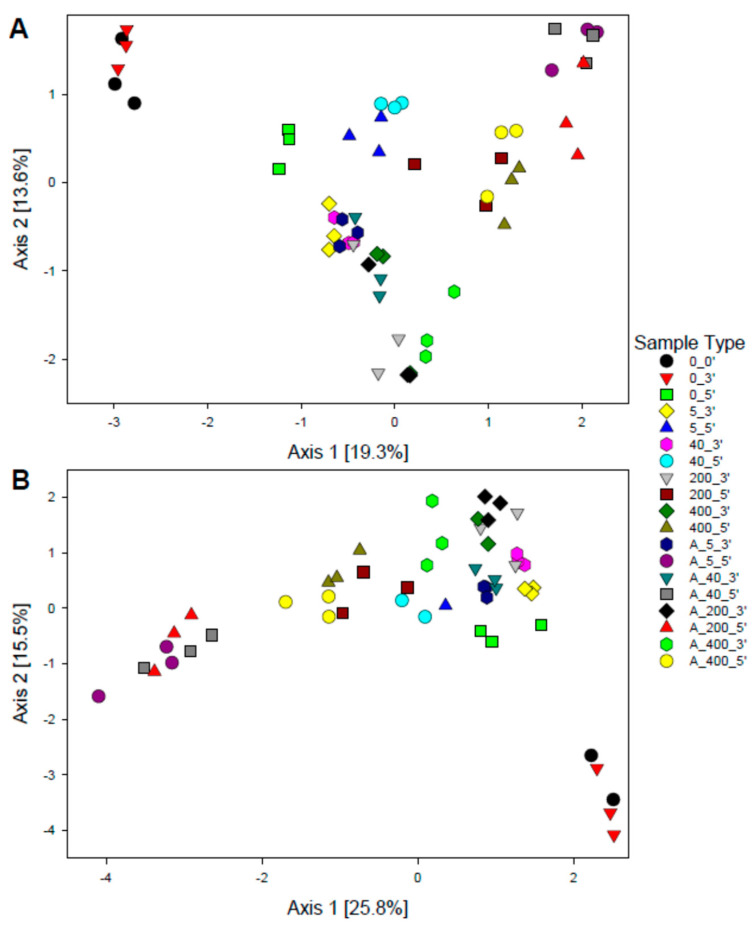
Principal coordinate analysis of the Bray–Curtis dissimilarity metric showing the effect of ibuprofen and ibuprofen/acetate treatments on the bacterial community composition based on OTUs from the 16S rRNA gene (panel **A**) and 16S rRNA (panel **B**). Sample code: A, amended with 1 mM acetate and ibuprofen per feeding; 0, 5, 40, 200, and 400 indicate supplemental ibuprofen concentrations of 0, 5, 40, 200, and 400 μM, respectively, given per feeding; 0′, 3′, and 5′, correspond to samples obtained at the start of the incubation, and after the third and fifth refeeding, respectively. Sampling times for unamended controls were according to those of the 400 μM ibuprofen treatment.

The total bacterial 16S rRNA gene abundance in the ibuprofen treatments and controls based on samples assessed after the third and fifth refeeding was essentially similar (ANOVA, *p* > 0.05) (Figure S3). The 16S rRNA gene abundance tended to be higher in unamended controls relative to ibubrofen-amended treatments, and after the third relative to the fifth refeeding. Such a pattern was less prominent on the rRNA level. The 16S rRNA abundance was, however, significantly higher in treatments relative to unamended controls after the third refeeding but lower than the unamended controls after the fifth refeeding (ANOVA, *p* < 0.05) (Figure S3). The alpha diversity from samples taken after the third and fifth refeeding revealed a significantly lower Shannon diversity (ANOVA, *p* ≤ 0.05) at the OTU level in samples amended exclusively with ibuprofen or ibuprofen-acetate relative to unamended controls (Figure S4). Species richness and evenness tended to follow the same trend; however, differences were not significant (ANOVA, *p* > 0.05) (Figure S4). Beta diversity visualization using principal coordinate analysis (PCoA) based on Bray–Curtis distances indicated that microbial communities in ibuprofen and ibuprofen-acetate treatments were distinct from the unamended controls and original sediment before incubation based on 16S rRNA gene and 16S rRNA sequences, respectively (Figure 2A,B). Consistently, two-way ANOSIM tests indicated that in samples amended exclusively with ibuprofen, both ibuprofen treatment (DNA: R = 0.7, RNA: R = 0.63, *p* < 0.0001) and incubation time (DNA: R = 0.98, RNA: R = 0.98, *p* < 0.0001) contributed significantly to the differences in the microbial community composition among the samples. Similarly, the ibuprofen-acetate treatment (DNA: R = 0.75, RNA: R = 0.72, *p* < 0.0001) and incubation time (DNA: R = 1, RNA: R = 0.99, *p* < 0.0001) also contributed significantly to the differences in the microbial community composition of the corresponding samples. The R-values greater than 0.6 indicated a rather strong dissimilarity between microbial communities from different treatments and time points. Communities from treatments amended with low and high ibuprofen concentrations (i.e., 5–40 and 200–400 µM, respectively) formed clusters separated along axis 1, suggesting a dose-dependent effect of ibuprofen (Figure 2A,B).

#### 3.2.2. General Phylum-Level Taxonomic Composition

Because of the scarcity of cultured ibuprofen degraders, structural genes associated with ibuprofen degradation as well as PCR assays targeting such genes are lacking. Hence, the 16S rRNA gene and transcript were targeted to characterize the response of the bacterial community after ibuprofen addition. The dominant phyla (>1% relative abundance) on both DNA and RNA levels were Proteobacteria, Chloroflexi, Acidobacteria, and Actinobacteria, followed by Firmicutes, Bacteroidetes, Gemmatimonadetes, Latescibacteria, and Nitrospirae (Figure S5A,B). Such phyla were among the significant top 10 based on the linear discriminant analysis (LDA) score on the DNA and RNA level, explaining differences among treatments (Figure S5C). The relative abundance of Proteobacteria, Acidobacteria, Bacteroidetes, Gemmatimonadetes, and Latescibacteria tended to increase in the presence of ibuprofen or ibuprofen and acetate (Figure S5). Actinobacteria and Chlorobi showed a variable response to the treatments when the DNA and RNA levels were compared. Interestingly, Acidobacteria, Gemmatimonadetes, and Latescibacteria appeared to be stimulated in treatments with low concentrations of ibuprofen (5–40 µM) in the absence of supplemental acetate on the DNA and RNA level. Proteobacteria were most abundant in amplicon libraries of treatments with acetate and high concentrations of ibuprofen (200–400 µM). Such a finding was attributed primarily to the Gammaproteobacteria, whose relative abundance was significantly higher relative to unamended controls (ANOVA, *p* < 0.05). Bacteroidetes responded to all ibuprofen concentrations with a high relative abundance in amplicon libraries. Such a high relative abundance was particularly prominent on the RNA level in treatments with high concentrations of ibuprofen (200–400 µM). Chloroflexi, Firmicutes, and Nitrospirae tended to decrease on average in relative abundance in response to ibuprofen. Archaeal sequences were generally less abundant than bacterial sequences in amplicon libraries, and affiliated primarily with Thaumarchaeota and Euryarchaeota. Likewise, they tended to decrease in response to ibuprofen treatments on the DNA and RNA level except for Thaumarchaeota, which tended to be stimulated on the RNA level with low ibuprofen concentrations.

#### 3.2.3. Family-Level Taxonomic Composition

**Figure 3:**
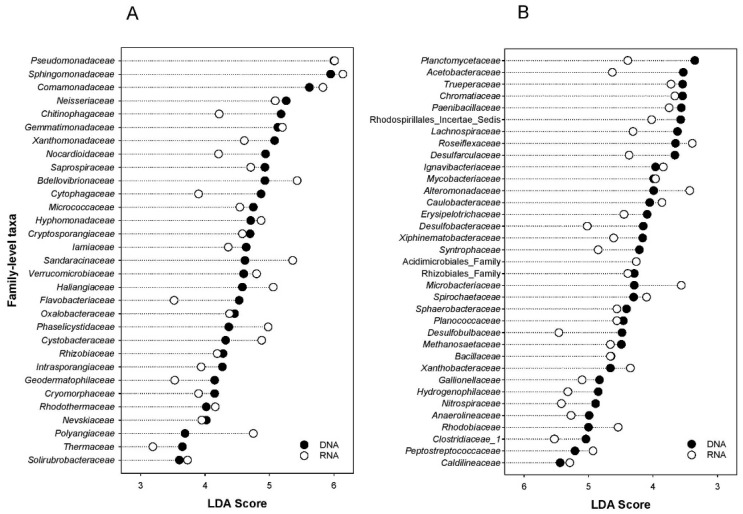
Linear discriminant analysis (LDA) scores on the DNA and RNA levels for families that were more (**A**) or less (**B**) abundant in treatments with ibuprofen relative to non-supplemented controls and displayed a consistent response on the DNA and RNA level.

*Pseudomonadaceae, Sphingomonadaceae*, and *Commamonadaceae* were the families with the highest LDA scores that had on average higher relative abundances in ibuprofen treatments compared to non-supplemented controls on the DNA and RNA level (Figure 3A), which was likewise reflected in the OTU-based analysis (Figure S6A,B) and the following phylum-level analysis. Notably, such families were consistently crucial in ibuprofen and ibuprofen-acetate treatments. Other families that had high LDA scores and higher relative abundances in amplicon libraries from ibuprofen treatments compared to non-supplemented controls on the DNA and RNA level included *Gemmatimonadaceae, Xanthomonadaceae, Nocardioidaceae, Flavobacteriaceae, Sandaracinaceae,* and *Cytophagaceae* (Figure 3A), suggesting that members of these families were stimulated in ibuprofen treatments. *Bdellovibrionaceae* was likewise stimulated. In contrast, LDA scores of family-level taxa that had lower relative abundances in ibuprofen treatments compared to non-supplemented controls on the DNA and RNA level suggested a negative impact of ibuprofen (Figure 3B). Many well-known anaerobes (e.g., *Caldilineaceae, Peptostreptococcaceae, Desulfobacteraceae,* and *Syntrophaceae*), as well as aerobic nitrifiers of the *Nitrospiraceae* and uncultured families, met such a criterion, suggesting that anaerobic processes, such as primary and secondary syntrophic, fermentations, and nitrification, might be impaired by ibuprofen.

#### 3.2.4. OTU-Level Taxa Associated with Ibuprofen Degradation

**Figure 4:**
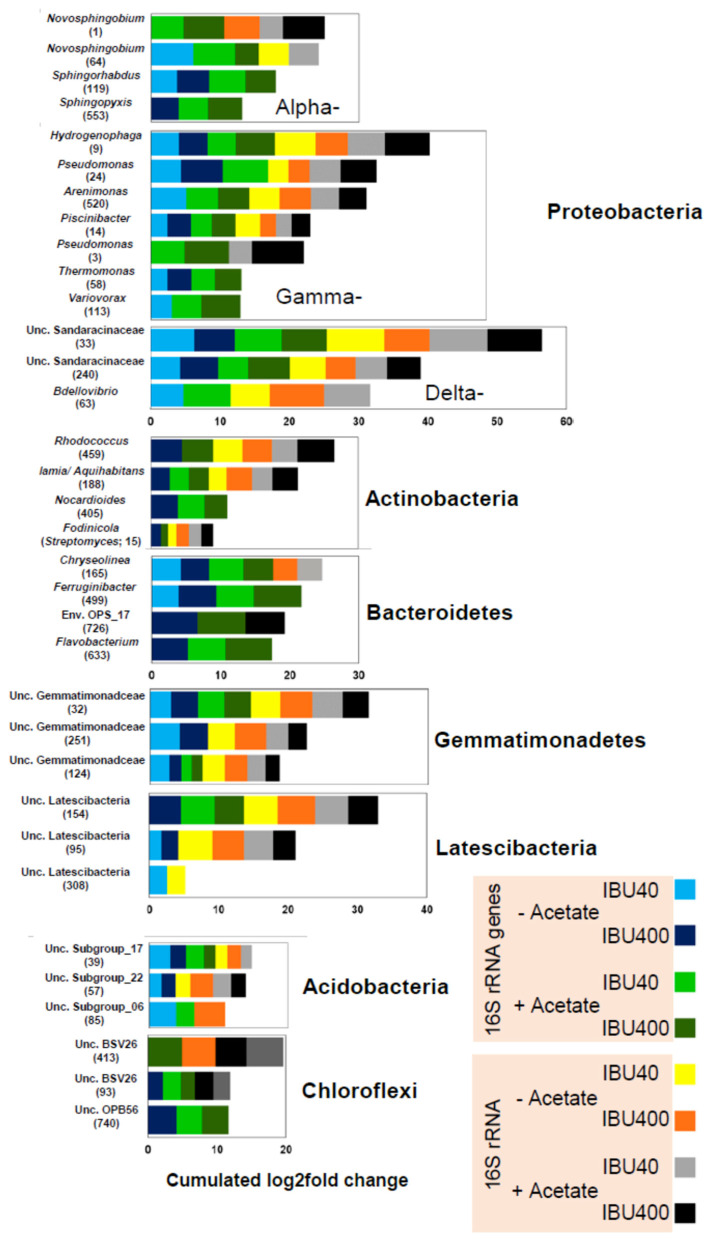
Log2fold change of ibuprofen-responsive OTUs summed up for all OTUs affiliating with the same (sub-) phylum (based on data from Table S2). A, DNA; B, RNA (cDNA). OTUs significantly enriched by ibuprofen relative to unamended controls sampled at the same time point had a Log2-fold change >0 at *p*-adj < 0.05. IBU40 and IBU400, ibuprofen amendment with 40 and 400 μM ibuprofen, respectively. IBA40 and IBA400, ibuprofen amendment with 40 and 400 μM ibuprofen, respectively, together with 1 mM acetate.

Differential abundance analysis was performed to identify ibuprofen-responsive OTU-level taxa. Taxa whose abundances significantly (*p* < 0.05) changed in ibuprofen treatments relative to unamended controls (log2foldchange > 0.5) were considered enriched and thus candidate taxa for representing ibuprofen degraders. Many OTUs (78 to 92) were enriched in ibuprofen treatments representative for high and low ibuprofen amendments (400 and 40 µM, respectively; Table S2). Consistent with the analyses on the phylum and family levels, OTUs enriched in response to ibuprofen affiliated primarily with Acidobacteria; Alpha-, Gamma-, and Deltaproteobacteria; Bacteroidetes; Gemmatimonadetes; and Latescibacteria. Cumulated log2fold change values in response to ibuprofen relative to unamended controls suggested a preferential enrichment of OTUs affiliating with Acidobacteria, Chloroflexi, Deltaproteobacteria, Gemmatimonadetes, Latescibacteria, and Saccharibacteria in treatments with low ibuprofen concentrations (40 µM) on the DNA level (Figure 4, Table S2). In contrast, OTUs affiliating with Alpha- and Gammaproteobacteria, Actinobacteria, Bacteroidetes, and Verrucomicrobia were more enriched in treatments with high ibuprofen concentrations (400 µM;) Figure 4). OTUs affiliating with Alpha-, Gamma-, and Deltaproteobacteria; Actinobacteria; and Chlorobi tended to be stimulated by supplemental acetate.

Proteobacterial OTUs dominated ibuprofen-responsive taxa in general. Such taxa included alphaproteobacterial *Novosphingobium, Hyphomicrobium*, and *Woodsholea*-affiliated OTUs; Gammaproteobacterial (Betaproteobacteriales affiliating) OTUs related to *Hydrogenophaga, Piscinibacter*, and *Vogesella*; Gammaproteobacteria OTUs related to *Pseudomonas* and *Arenimonas*; as well as OTUs affiliating with *Bdellovibrio* and distantly related to *Sandaracinus*. *Rhodococcus*, *Iamia, Aquihabitans*, *Nocardioides*, and *Fodinicola* related OTUs along with uncultured taxa were significant among the Actinobacteria. Ibuprofen-enriched important taxa of the Bacteroidetes included *Chryseolinea, Ferruginibacter, Flavobacterium*, and uncultured taxa. Ibuprofen-enriched OTUs of the Gemmatimonadaceae and Latescibacteria were distantly related to *Gemmatimonas* sp. and to uncultured organisms, respectively. OTUs affiliating with subgroups 6, 17, and 22 within the Acidobacteria were enriched in response to ibuprofen. Notably, certain phyla that showed on average a generally variable or even negative response to ibuprofen nevertheless contained OTUs that were enriched in response to ibuprofen (Table S2), demonstrating the need for high taxonomic resolution on the OTU level. Such phyla were Chloroflexi (Figure 4, Table S2), Chlorobi (e.g., OTUs 93 and 740), Nitrospirae (e.g., OTU 7 related to *Nitrospira moscoviensis*), and Verrucomicrobia (e.g., OTU 162 related to *Prosthecobacter* sp.). Hitherto uncultured groups (e.g., NS9_marine_group, env. OPS_17, KD4-96; Table S2) enriched by ibuprofen indicate new potential ibuprofen degraders in hyporheic zone sediments. Most of the OTUs enriched in ibuprofen treatments following incubation were detected in the original community and/ or the unamended controls (Table S3). OTUs 1, 15, 32, 95, 39, and 93, indicative of *Novosphingobium* sp. (Alphaproteobacteria), *Fodinicola* sp. (Actinobacteria), uncultured Gemmatimonadetes, uncultured Latescibacteria, Acidobacterial subgroup 17, and uncultured Chloroflexi of the BSV26 group, respectively, had relative abundances of greater than 0.1% in the original community on the DNA level in the non-rarified dataset (data not shown).

### 3.3. Quantification of Representative Ibuprofen-Enriched OTUs

**Figure 5:**
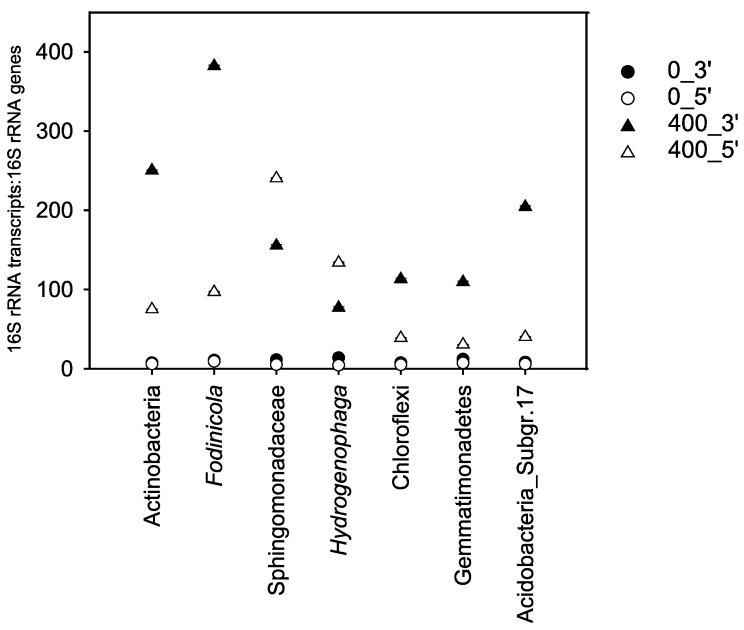
16S rRNA (cDNA) to 16S rRNA gene ratios determined by qPCR for selected taxa stimulated by ibuprofen in the 400 μM treatment (Figure 1 and Figure 4) as an indicator of taxon-specific activity. Values are the arithmetic means of triplicate incubations. Error bars indicate the standard deviation but are smaller than the symbol size and therefore not apparent. Sample code: 0 and 400 indicate supplemental ibuprofen concentrations in μM given per feeding; 3′ and 5′ correspond to samples obtained after the third and fifth refeeding, respectively. Sampling times for unamended controls were according to those of the 400 μM ibuprofen treatment.

An increase in relative abundance in amplicon libraries does not necessarily indicate stimulation. Thus, qPCR was utilized to verify ibuprofen-stimulated taxa as indicated by relative abundance data (see previous sections) using 16S rRNA (cDNA) to 16S rRNA gene ratios as an indicator of taxon-specific activity. Representative OTUs were selected based on significantly higher expression of 16S rRNA genes (ANOVA, *p* ≤ 0.05) in 400 µM ibuprofen treatments relative to unamended controls (Figure 5, Table S1), which agreed with the Deseq2 differential abundance data analysis. This highlighted the reliability of the differential abundance approach to identify potential ibuprofen-responsive taxa in hyporheic zone sediment microcosms.

### 3.4. Ibuprofen-Degrading Strains CN1 and MAH1

**Figure 6:**
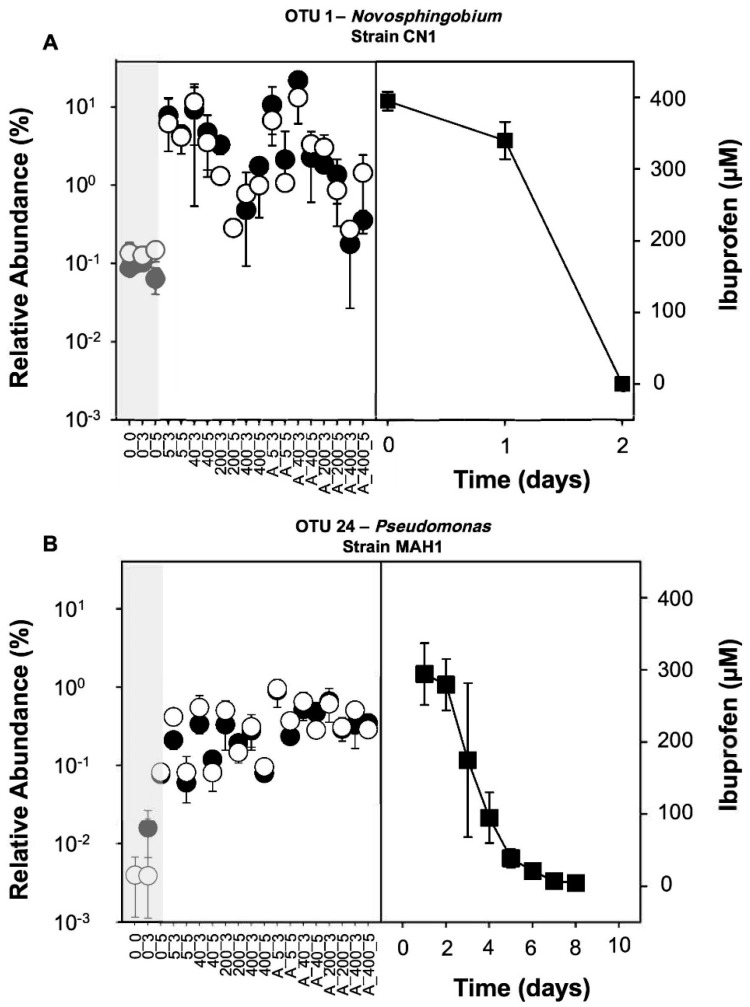
Effect of ibuprofen on the relative abundance of OTUs in 16S rRNA gene (DNA) and 16S rRNA (RNA or cDNA)-derived amplicon libraries from oxic hyporheic zone sediment microcosms (Figure 1) affiliating with ibuprofen-degrading strains *Novosphingobium* CN1 (**A**) and *Pseudomonas* MAH1 (**B**), and the capacity of both strains to degrade ibuprofen under oxic conditions. The grey box indicates unsupplemented oxic control microcosms. Values represent the arithmetic means of triplicates, and error bars indicate standard deviations. Filled and open circles, DNA and RNA (cDNA) level, respectively; filled squares, ibuprofen concentration. Sample code: A, amended with 1 mM acetate and ibuprofen per feeding; 0, 5, 40, 200, and 400 indicate supplemental ibuprofen concentrations in μM given per feeding; 0′, 3′, and 5′ correspond to samples obtained at the start of the incubation, and after the third and fifth refeeding, respectively. Sampling times for unamended controls were according to those of the 400 μM ibuprofen treatment.

The two strains isolated from the 400 µM ibuprofen-only treatments (Figure 1), affiliated with ibuprofen-responsive OTUs 1 (*Novosphingobium* related) and 24 (*Pseudomonas* related; Figure 5). These representative OTUs for *Sphingomonadaceae* and *Pseudomonadaceae*, respectively, were present in the original hyporheic zone community prior to incubation at a relative abundance of approximately 0.01–0.1%, and stimulated by ibuprofen on the DNA and RNA level (Figure 6A,B and Figure S6; Table S2). The stimulation of OTU 1 generally resulted in higher relative abundances than OTU 24 and tended to be most prominent in treatments with low ibuprofen concentrations (Figure 5). The relative abundance of OTU 24 was more similar across all treatments. 16S rRNA gene similarities of CN1 and MAH1 to representative sequences of OTUs 1 and 24, respectively, were >97%. Strain CN1 consumed approximately 400 µM of ibuprofen within two days (Figure 6A). CN1 had a 16S rRNA gene similarity of 96.8% and 96.3% to the *Novosphingobium flavum* strain UCM-28 (Acc. Nr. NR_152007) and *N. aromaticivorans* strain IFO 16084 (Acc. Nr. NR_112090), respectively, and clustered with other organisms of the genus *Novosphingobium* (Figure S7A), suggesting that strain CN1 represents a new ibuprofen degrader of the genus. Strain MAH1 consumed approximately 300 µM of ibuprofen within eight days (Figure 6B). A 16S rRNA gene similarity of 99.9% of MAH1 to *Pseudomonas thivervalensis* (Acc. Nr. KF528727) and clustering with *P. thivervalensis* (Figure S7B) suggests MAH1 as an ibuprofen-degrading strain of this species.

## 4. Discussion

### 4.1. Biodegradation of Ibuprofen in the Hyporheic Zone Sediments

Microbial degradation of ibuprofen was demonstrated in the hyporheic zone sediments (Figure 1). Thus, our findings extend previous studies reporting the attenuation of ibuprofen in engineered and natural environments [6,9,10,11,12,44]. Consistent with previous studies [45,46], abiotic losses due to sorption and hydrolysis played a marginal role in total ibuprofen removal, demonstrating that ibuprofen removal was indeed mainly due to biodegradation (Figure S1). The ibuprofen concentration was observed to influence the rate of degradation after the first feeding. The 400 µM ibuprofen treatment exhibited a nine-day lag phase in contrast to lower concentrations, whose disappearance exhibited no significant delay. This is likely attributable to an inhibitory effect on the microbial activity as has been reported for ibuprofen concentrations exceeding 50 mg L^−1^ (242 µM) in which a decline in oxygen respiration and microbial diversity was observed in activated sludge [47]. Subsequent rapid depletion of ibuprofen following refeeding irrespective of the initial concentration suggests a rapid enrichment of microbes capable of ibuprofen degradation and thus adaption of the microbial community (Figure 1A–D). A similar pattern was reported in a recent study [47], where an activated sludge microbial community adapted to high concentrations of ibuprofen (5000 mg L^−1^; 24 mM) after long-term exposure, suggesting a rather high limit for ibuprofen tolerance.

Microcosms containing supplemental acetate as the primary carbon source exhibited a delayed onset of ibuprofen degradation following initial spiking (Figure 1). This is in contrast to ibuprofen oxidation by enzymes activated during the initial degradation of acetate rather than ibuprofen, a phenomenon associated with cometabolic degradation [12]. The preferential consumption of acetate by the indigenous hyporheic zone bacteria capable of ibuprofen consumption is a more likely explanation, considering that acetate is a more easily degradable substrate than ibuprofen. In the latter case, ibuprofen is likely degraded upon acetate depletion. Refeeding the microcosms with ibuprofen-acetate, however, exhibited a rapid ibuprofen degradation similar to ibuprofen-only microcosms, suggesting that ibuprofen degraders were enriched in the absence and presence of supplemental acetate. In most cases, similar taxa were stimulated in treatments with ibuprofen only and acetate/ ibuprofen (Figure S6), supporting the view that metabolic degradation of ibuprofen was significant in the sediments and that many taxa in the hyporheic zone are prone to respond to ibuprofen as a source of carbon and energy.

The different transformation intermediates 1-, 2-, 3-hydroxy- and carboxyibuprofen were transiently observed during ibuprofen degradation (Figure S2). Moreover, these ibuprofen degradation intermediates were likewise detected previously in situ in the river Erpe hyporheic zone sediments [22], indicating ongoing ibuprofen biodegradation in situ as a primary attenuation mechanism for ibuprofen and the utilization of different metabolic pathways characteristic of a diverse bacterial community (Figure S8).

Indeed, previous studies using pure cultures have shown variable biodegradation pathways for ibuprofen [48]. 2-hydroxyibuprofen was the most abundant transformation intermediate detected during ibuprofen degradation in the hyporheic zone sediments (Figure S2). 2-hydroxyibuprofen is generated by an aliphatic monooxygenase activity followed by a series of other enzymes, leading to the eventual production of 3-hydroxy-*cis*,*cis*-muconic acid, which enters the tricarboxylic acid cycle in *Bacillus thuringiensis* B1 [15]. *Nocardia NRRL 5646* was shown to employ a carboxylic acid reductase enzyme system that reduces the carboxylic functional group of ibuprofen to the corresponding alcohol, which is then acetylated [49]. Thus, one of the initial reactions of ibuprofen utilization in our microcosms was the transformation of the aliphatic chain as observed with such isolates and other environmental samples [9,18].

Further characterized ibuprofen degraders producing degradation intermediates not detected in our study include *Sphingomonas* Ibu-2 and *Variovorax* Ibu-1. *Sphingomonas* Ibu-2 hydroxylates ibuprofen to isobutylocatechol following CoA ligation, which is then cleaved to 5-formyl-2-hydroxy-7-methylocta-2,4-dienoic acid, before oxidation to 2-hydroxy-5-isobutylhexa-2,4-dienedioic acid [13,50]. *Variovorax* Ibu-1 degrades ibuprofen via ring-hydroxylated ibuprofen [14]. However, such degradation intermediates were only detected when further metabolism was inhibited by 3-fluorocatechol, suggesting that such intermediates are subject to rapid turnover and will escape detection during routine analyses. 1,2-dihydroxyibuprofen has previously been reported as a probable dead-end product of fungal ibuprofen metabolism [51]. As these compounds were not measured in the current study, conclusions on the importance of such ibuprofen degradation pathways in our sediment microcosms remain elusive. Nevertheless, all of the biodegradation pathways named above converge in the tricarboxylic acid cycle, allowing for the assimilation of ibuprofen carbon and potentially the mineralization of ibuprofen to CO_2_. The cumulative findings of the current study, i.e., the enrichment of many taxa in response to ibuprofen (Figure 4, Table S2), the acceleration of ibuprofen degradation during incubation (Figure 1), utilization of ibuprofen by the two isolates as the only carbon and energy source to support their growth (Figure 6), the lack of inhibitory effects on ibuprofen degradation after five refeedings (Figure 1), and the disappearance of the ibuprofen transformation products (Figure S2), argue in favor of mineralization and assimilation of ibuprofen carbon by hyporheic zone sediment microorganisms.

### 4.2. Bacterial Community Structure and Diversity

The significant increase in 16S rRNA-based copy numbers and sequence abundance in ibuprofen-amended samples relative to controls after the third refeeding (Figures S3 and S5), suggests a strong stimulation of specific ibuprofen responders, since rRNA abundance serves as a proxy for activity [52]. The decline in rRNA abundance in most treatments after the fifth refeeding corresponds with the sampling, which was carried out at the end of the incubation when ibuprofen was almost depleted in most microcosms (see Figure 1). Despite a significant decline in the Shannon diversity index (ANOVA, *p* < 0.05) in the high ibuprofen treatments (200 and 400 µM; Figure S4), the River Erpe hyporheic zone sediments hosted a taxonomically diverse microbial community (Figures S4 and S5; Table S2). Accumulating evidence suggests a positive correlation between taxonomic diversity and some specific microbially mediated processes, such as micropollutant degradation [53,54].

#### 4.2.1. Generalized Ecological Niches of Ibuprofen-Responsive Phyla

OTUs affiliating with the phyla Proteobacteria, Bacteroidetes, Chloroflexi, Acidobacteria, Actinobacteria, and Gemmatimonadetes were particularly shown to positively respond to ibuprofen amendment (Figure 4, Figures S5 and S6). The higher degradation rates with subsequent ibuprofen addition (after initial lag; Figure 1)**,** concurrent with the high 16S rRNA gene/transcript abundance ratio in amended relative to unamended sediment samples (Figure 5), suggested that members of these phyla were enriched and dominated the degrader population. Previously, Proteobacteria and Bacteroidetes were observed in studies on a subsurface flow-constructed wetland system treating ibuprofen-contaminated wastewater, and in oxic ibuprofen-amended microcosms with activated sludge [6,47], indicating the presence and response to ibuprofen of members belonging to these key phyla in diverse environments. Proteobacteria were the most responsive to high ibuprofen concentrations (Figure 4 and Davids et al. [47]). Their response may be attributed to their general functional traits as aromatic compound degraders and the capability to quickly respond to substrate availability [55,56]. Microcosms with acetate as a primary substrate exhibited similar patterns relative to ibuprofen-only treatments in terms of relative phylum abundance (Figure S5). However, the relative abundance of Gammaproteobacteria was significantly higher (ANOVA, *p* < 0.05) in ibuprofen-acetate-containing than in ibuprofen-only microcosms. This would indicate that the Gammaproteobacteria stimulated by ibuprofen are likewise capable of acetate utilization.

Members of the different phyla, such as Acidobacteria, Gemmatimonadetes, and Latescibacteria, positively responded to lower ibuprofen concentrations (5 and 40 µM) and less to higher concentrations (200 and 400 µM), while the opposite was observed for Proteobacteria (Figure 4, Figure S5), suggesting that some bacteria can use micropollutants as a substrate within a certain range of concentrations, above which it turns toxic or inhibitory. Thus, our study suggests distinct ecological niches for Proteobacteria that reflect the lifestyles of r-strategists, and Acidobacteria, Gemmatimonadetes, and Latescibacteria that reflect the lifestyles of K-strategists.

#### 4.2.2. Putative Taxa Associated with Degradation of Ibuprofen in Oxic Hyporheic Zone Sediments

New and known bacteria enriched in ibuprofen-amended microcosms relative to unamended controls were associated with degradation of ibuprofen (Figure 4 and Figure S6, Table S2). Using 40 and 400 µΜ ibuprofen concentrations as representative concentrations for low and high ibuprofen concentrations, diverse families and genera from many phyla were enriched. Some of these bacteria were also observed in microcosms with intermediate ibuprofen concentrations (Figure S6). Moreover, most of the enriched taxa were detected in the original hyporheic zone sediment bacterial community, suggesting their involvement in ibuprofen degradation in situ (Table S3).

Stimulation of Proteobacteria-affiliated taxa by ibuprofen was most significant (Figure 4 and Figures S5, S6; Table S2). The enrichment of members belonging to the Alphaproteobacteria, such as *Sphingomonadaceae* and *Hyphomonadaceae*, corresponded to previous studies in which genera belonging to these families were associated with the degradation of xenobiotics [26,57,58]. Moreover, *Hyphomicrobium*, a genus from the family *Hyphomicrobiaceae*, has been previously associated with assimilation of 2,4-dichlorophenol, a soil and groundwater contaminant [26]. Genera closely related to *Sphingopyxis*, *Sphingorhabdus*, and *Novosphingobium* affiliating with the ibuprofen-enriched family *Sphingomonadaceae* have been isolated from a wide variety of environments, including freshwater and marine sediments, and were associated with the degradation of a wide variety of natural aromatic compounds and xenobiotics [59,60]. The ibuprofen degradation kinetics of *Novosphingobium* strain CN1 isolated in this study showed a high capacity to quickly degrade ibuprofen (Figure 5) and were in the range of ibuprofen degradation rates observed after the fifth refeeding of ibuprofen in hyporheic zone sediments (Figure 1). The high relative abundance of CN1 representing OTU 1 in treatments with low rather than with high concentrations of ibuprofen suggest that the *Novosphingobium* strain CN1 represents a copiotrophic organism whose growth is impaired in situ in the presence of high ibuprofen concentrations. The isolation of the ibuprofen-degrading *Novosphingobium* strain CN1 in this study along with the ibuprofen-degrading *Sphingomonas* IBU-2 [13] extends previous observations to hyporheic zones, consolidates correlations, and emphasizes the biodegradation potential associated with *Sphingomonadaceae*.

New and known members of the Betaproteobacteriales (considered as part of the Gammaproteobacteria) family *Comamonadaceae* exhibited a positive association with ibuprofen. *Comamonadaceae* is among the families with members previously reported to aerobically degrade aromatic compounds [26]. One of the characterized ibuprofen degraders belonging to this family is *Variovorax* Ibu-1 [14]. In a study exploring the co-occurrence patterns between organic micropollutants and bacterial community structure, the ibuprofen-enriched genus *Hydrogenophaga* (*Comamonadaceae*) was among bacteria significantly correlated to micropollutants and believed to host enzymes for the biotransformation of specific micropollutants [61]. The ibuprofen-enriched genus *Piscinibacter*, whose closest cultivated relative *Piscinibacter aquaticus* (basonym: *Methylibium aquaticum*) was isolated from a eutrophic freshwater pond [62], and the family *Oxalobacteraceae* were hitherto unassociated with the degradation of aromatic compounds (Figure 4, Figure S5 and S6; Table S2). However, these taxa belong to the order Burkholderiales, whose other families, including *Comamonadaceae*, are associated with such potentials. It is, therefore, likely that Burkholderiales members may have ibuprofen biotransformation potential. Enrichment of unknown genera belonging to the family *Nitrosomonadaceae* by ibuprofen corroborates the reported potential of members of this family, such as *Nitrosomonas*, in bioremediation. Through the activity of ammonia monooxygenase, most ammonia-oxidizing bacteria in this family can co-metabolize micropollutants [12]. Potential indirect effects like enhanced ammonia release from biomass turnover due to ibuprofen-stimulated microbial predation (see below) and ammonification might even allow for enhanced growth of certain nitrifiers in the presence of ibuprofen.

Significant enrichment of the Gammaproteobacterial family *Pseudomonadaceae* in ibuprofen treatments is in congruence with previous findings, where *Pseudomonadaceae* was reported to be involved in the biodegradation of polyaromatic compounds, such as naphthalene [59]. The genus *Pseudomonas* accommodates many isolates capable of aromatic compound degradation [55]. Our isolate *Pseudomonas thivervalensis* MAH1 grew with ibuprofen as the sole carbon and energy source, demonstrating the ibuprofen degradation capabilities of this genus (Figure 6B). Interestingly, the relative abundance of OTU 24 indicative of the strain MAH1 was essentially similar in all ibuprofen treatments, although ibuprofen degradation of MAH1 was rather slow (Figure 5). Such data suggest that *Pseudomonas thivervalensis* MAH1 represents an oligotrophic organism with a high ibuprofen tolerance. The ibuprofen-enriched genus *Arenimonas* (*Xanthomonadaceae*) has been previously associated with degradation of drugs, such as penicillin and carbamazepine, in activated sludge and contaminated soils [63], highlighting the importance of Gammaproteobacteria for biodegradation.

The ibuprofen-enriched Deltaproteobacteria were from the orders Myxococcales and Bdellovibrionales (Figure 4, Figures S5 and S6; Table S2). Myxococcales degrade complex organic substrates, possess sophisticated secondary metabolism, and have a predatory lifestyle [64,65]. *Bdellovibiro* sp. enriched in response to ibuprofen indicated the stimulation of a group recognized as predatory organisms feeding on Gram-negative bacteria [66]. Predation on Gram-negative ibuprofen degraders might likewise increase the microbial biomass turnover and thus represent a source of variation in the data set, limiting ibuprofen-dependent stimulation. Thus, we suggest that the enrichment of Deltaproteobacteria in ibuprofen treatments is due to indirect effects, i.e., stimulation of ibuprofen-degrading Gram negatives serving as prey, rather than due to the direct ibuprofen degradation capabilities of this group.

Many taxa affiliated with Actinobacteria were also enriched in response to ibuprofen (Figure 4, Figures S5 and S6; Table S2). This phylum is known to accommodate many species involved in the degradation of complex compounds, including phenol, diesel oil, n-alkanes, and polycyclic aromatic hydrocarbons [1,55,67]. Such taxa included *Rhodococcus* sp. and *Nocardioides*-related OTUs. Both genera are well known for their capabilities to degrade aromatic compounds [55]. Previously, the *Nocardia* strain NRRL 5646 of the Actinobacteria that transformed ibuprofen using the carboxylic acid reductase enzyme system has been characterized [49]. *Fodinicola* sp., closely related to the pesticide-degrading genus *Streptomyces* [68], a new *Iamia* related and *Illumatobacter* sp. were further examples for ibuprofen-enriched taxa. *Ilumatobacter* sp. was among Actinobacteri-affiliated taxa previously considered as potential indicators for exposure to organic pollutants [69], further highlighting the importance of known and hitherto undetected taxa of the Actinobacteria for biodegradation of pollutants in the environment.

Ibuprofen-enriched genera affiliating with the phylum Bacteroidetes include *Terrimonas* and *Ferruginibacter* (family *Chitinophagaceae*, order Sphingobacteriales; (Figure 4, Figures S5 and S6; Table S2). *Terrimonas* has been shown to degrade benzo[a]pyrene, a polycyclic aromatic hydrocarbon (PAH) [70]. The genes encoding the PAH-ring hydroxylating dioxygenase enzymes involved in the first hydroxylation steps of benzo[a]pyrene and other PAHs under aerobic conditions may predictably be involved in ibuprofen degradation. *Flavobacterium*, a genus in the family *Flavobacteriaceae*, was also stimulated in ibuprofen treatments as also previously reported [6]. Members of this genus have been isolated on 2,4-dichlorophenol [71] and also associated with the transformation of pharmaceuticals [72], suggesting capabilities for aromatic compound degradation. Bacteroidetes-affiliated genera like *Chryseolinea* and other unclassified genera in the family env. OPS_17 (order Sphingobacteriales) were positively associated with ibuprofen amendment, extending the reported association of the phylum with the degradation of high-molecular-weight organic compounds, including petroleum hydrocarbons [73], and supporting the view that Bacteroidetes include environmentally relevant aerobic pollutant degraders.

Few uncultured taxa from low-abundance phyla whose ecophysiology is not well characterized were also enriched in ibuprofen treatments, suggesting their potential contribution to the degradation of ibuprofen. Enrichment of hitherto unclassified families belonging to subgroups 6, 17, 22, and other Acidobacterial families in the phylum Acidobacteria in response to ibuprofen extends previous reports on the association of members of this phylum with the degradation of contaminants like polychlorinated biphenyls [74], and petroleum compounds [75]. Taxa of the phyla Gemmatimonadetes and Latescibacteria responded to ibuprofen treatments. *Gemmatimonas aurantiaca* is capable of utilizing benzoate as the sole carbon and energy source, and the genus *Gemmatimonas* was related to linear alkylbenzene sulfonate degradation in a bioreactor [57]. Thus, there is some support for the hypothesis that such uncultured taxa are likewise involved in ibuprofen degradation [67]. However, even less information is available for other ibuprofen-stimulated uncultured taxa of the Armatimonadetes, Chloroflexi, Chlorobi, and some Candidate divisions (Table S2), demonstrating the need for further research to consolidate the role of such taxa for biodegradation.

## 5. Conclusions

Microbial degradation of micropollutants, usually occurring in the ng to µg L^−1^ range, is a critical attenuation mechanism in the hyporheic zone [22]. Evidence for the capability of microbes to respond to in situ relevant minute concentrations of organic compounds comes from biosensor studies utilizing isolates obtained by enrichments with high growth-supportive substrate concentrations. Concentrations of organic compounds down to the pg L^−1^ range suffice to induce the transcription of catabolic genes associated with the degradation of such compounds, demonstrating that microbes growing on high substrate concentrations also respond to trace quantities of their substrate by consuming it (e.g., [76]). Thus, microbes enriched with ibuprofen concentrations higher than those usually occurring in situ have a high likelihood of being capable of degrading ibuprofen at in situ relevant concentrations in the hyporheic zone.

Primarily, Proteobacteria along with diverse hitherto unknown and known microbes were associated with the degradation of ibuprofen based on a correlative data set of relative abundance data from 16S rRNA and 16S rRNA gene amplicons. qPCR of selected taxa and calculation of the ratio of 16S rRNA to 16S rRNA genes as an indicator of a potential specific activity [77] supported the relative abundance data. Isolation of ibuprofen degraders provided causality for the ibuprofen enrichment of two OTUs and their ibuprofen degradation capabilities. Such OTUs likewise indicated that isolated ibuprofen degraders were present in situ and prone to quickly respond to the presence of ibuprofen. In the absence of characterized functional genes associated with emerging micropollutants, phylogeny-based analysis of microbial communities provides an alternative step to understand the interaction between potential degraders and the micropollutants. Thus, by exploiting complementary data from process studies, 16S rRNA and 16S rRNA gene amplicon sequencing data, the ratio of 16S rRNA to 16S rRNA genes as an indicator of the potential activity of bacterial taxa, and characterization of isolates, we provide evidence for many hitherto unknown ibuprofen degraders and provide new ibuprofen-degrading model organisms relevant for an important ecosystem service in a poorly characterized environment from a microbiological perspective.

## Supplementary Materials

The following are available online at https://www.mdpi.com/2076-2607/8/8/1245/s1, Figure S1: Control microcosms for the ibuprofen degradation experiment (Figure 1). ‘Non-supplemented’ represents the unamended sediment. The abiotic controls ‘Sorption’ and ‘Hydrolysis’ contained autoclaved sediment and river water, respectively, and were amended with 200 μM ibuprofen. Value s are the arithmetic means of three replicate incubations. Red and blue-headed arrows indicate sampling of the sediment for nucleic acids extraction after third and fifth re-spiking, respectively. Error bars indicating standard deviations are smaller than the size of the symbols and therefore not apparent. Figure S2: Ibuprofen transformation products in oxic hyporheic zone sediment microcosms amended with 200 μM of ibuprofen (4th respike; see Figure 1B). Values are the arithmetic mean of three replicate incubations, and error bars indicate standard deviations. Some standard deviations are smaller than the symbol size and therefore not apparent. Figure S3: Copy numbers of the 16S rRNA gene and 16S rRNA detected in total bacterial community. Sample code: A, amended with 1 mM acetate and ibuprofen per feeding; 0, 5, 40, 200, and 400, indicate supplemental ibuprofen concentrations of 0 μM, 5 μM, 40 μM, 200 μM and 400 μM, respectively, given per feeding; 0′, 3′, and 5′, correspond to samples obtained at the start of the incubation, and after the third and fifth refeeding, respectively. Sampling times for unamended controls were according to those of the 400 μM ibuprofen treatment. Values are the arithmetic average of three replicates. Error bars indicate standard deviation values. Figure S4: Alpha diversity and richness estimators of 16S rRNA gene and 16S rRNA obtained from Illumina amplicon sequencing. Sample code: A, amended with 1 mM acetate and ibuprofen per feeding; 0, 5, 40, 200, and 400, indicate supplemental ibuprofen concentrations of 0 μM, 5 μM, 40 μM, 200 μM and 400 μM, respectively, given per feeding; 0′, 3′, and 5′, correspond to samples obtained at the start of the incubation, and after the third and fifth refeeding, respectively. Sampling times for unamended controls were according to those of the 400 μM ibuprofen treatment. Values are the arithmetic average of three replicates. Error bars indicate standard deviation values. Figure S5: Relative abundance of major bacterial (greater than 1% relative abundance) and archaeal phyla on DNA (A) and RNA (cDNA, B) level, as well as corresponding LDA scores (C). Archaea are indicated by the grey box. Values and error bars represent means from all time points per treatment (see Figure 1 and Table S1) of up to 12 samples and standard deviation, respectively. 0, unamended controls; 5–40, ‘low ibuprofen treatments’ with 5 and 40 μM ibuprofen; 200–400, ‘high ibuprofen treatments’ with 200 and 400 μM ibuprofen; A5–40, ‘low ibuprofen treatments’ with supplemental 1 mM acetate; A200–400, ‘high ibuprofen treatments’ with supplemental 1 mM acetate. Filled and open circles, DNA and RNA (cDNA) level, respectively. Figure S6: Heat map of LEfSe identified top 50 most differentially abundant taxa based on 16S rRNA gene (A) and 16S rRNA (cDNA; B) amplicon analysis after the third and fifth refeeding (Figure 1). OTUs are sorted according to the difference in their mean read counts in ibuprofen amended and unamended samples. OTUs with a linear discriminant analysis score of ≥4 and their phylogenetic affiliation are shown. The color code reflects median, as well as upper and lower quantiles of read counts normalized to the total number of reads per OTU from all samples. Sample code: A, amended with 1 mM acetate and ibuprofen per feeding; 0, 5, 40, 200, and 400 indicate supplemental ibuprofen concentrations in μM given per feeding; 0′, 3′, and 5′ correspond to samples obtained at the start of the incubation, and after the third and fifth refeeding, respectively. Sampling times for unamended controls were according to those of the 400 μM ibuprofen treatment. *, Samples with significant differential relative abundance compared to the start of the incubation based on DESeq2 (adj *p* < 0.1). Figure S7: Phylogenetic tree reconstructed with the neighbour-joining method based on 16S rRNA gene sequences of strain CN1 and other *Novosphingobium* species (panel A) and strain MAH1 and other Pseudomonas species (panel B), showing their position among phylogenetic neighbours. *Aquicella siphonis* strain SGT-108 was used as an outgroup. Bootstrap values (based on 1000 replications) above 70% are shown at branch nodes. Figure S8: Hypothetical ibuprofen degradation pathways in oxic hyporheic zone sediments based on the transformation products identified in the current study and previous studies as reported in [48]. Table S1: Sequences and annealing temperature of primer sets targeting representative ibuprofen-responsive taxa (400 µM ibuprofen treatment; Figure 2 and Figure 3, Table S2). The primers were used to amplify the 16S rRNA gene and corresponding 16S rRNA transcripts (cDNA). Table S2: Classification of bacterial OTUs enriched by ibuprofen relative to unamended controls sampled at the same time point, and closest cultured relatives of OTU representative 16S rRNA gene sequences. Significant (*p*-adj < 0.05) Log2-fold change >0 are reported as determined by Deseq2. IBU40 and IBU400, ibuprofen amendment with 40 and 400 µM ibuprofen, respectively. IBA40 and IBA400, ibuprofen amendment with 40 and 400 µM ibuprofen, respectively, together with 1 mM acetate. Table S3: Relative abundance of bacterial OTUs from the original microbial community enriched by 40 and 400 µM ibuprofen treatments at DNA and RNA levels under oxic conditions after incubation. 0, represents unamended samples and 40 and 400 correspond to samples amended with 40 and 400 µM ibuprofen, respectively. 0′, 3′, and 5′ correspond to samples obtained at the start of the incubation, and after the third and fifth refeeding, respectively. Data represents the mean of triplicate samples in % of total rarified reads (uniform sequencing depth of 10,767 per sample) ± standard error of mean (SEM).
