# Supplementary material for: Ibuprofen Degradation and Associated Bacterial Communities in Hyporheic Zone Sediments
Source: Microorganisms. 2020 Aug 16;8(8):1245. doi: 10.3390/microorganisms8081245 (PMC7464344; doi:10.3390/microorganisms8081245)
Supplement: Supplementary file 1 [file microorganisms-08-01245-s001.pdf]

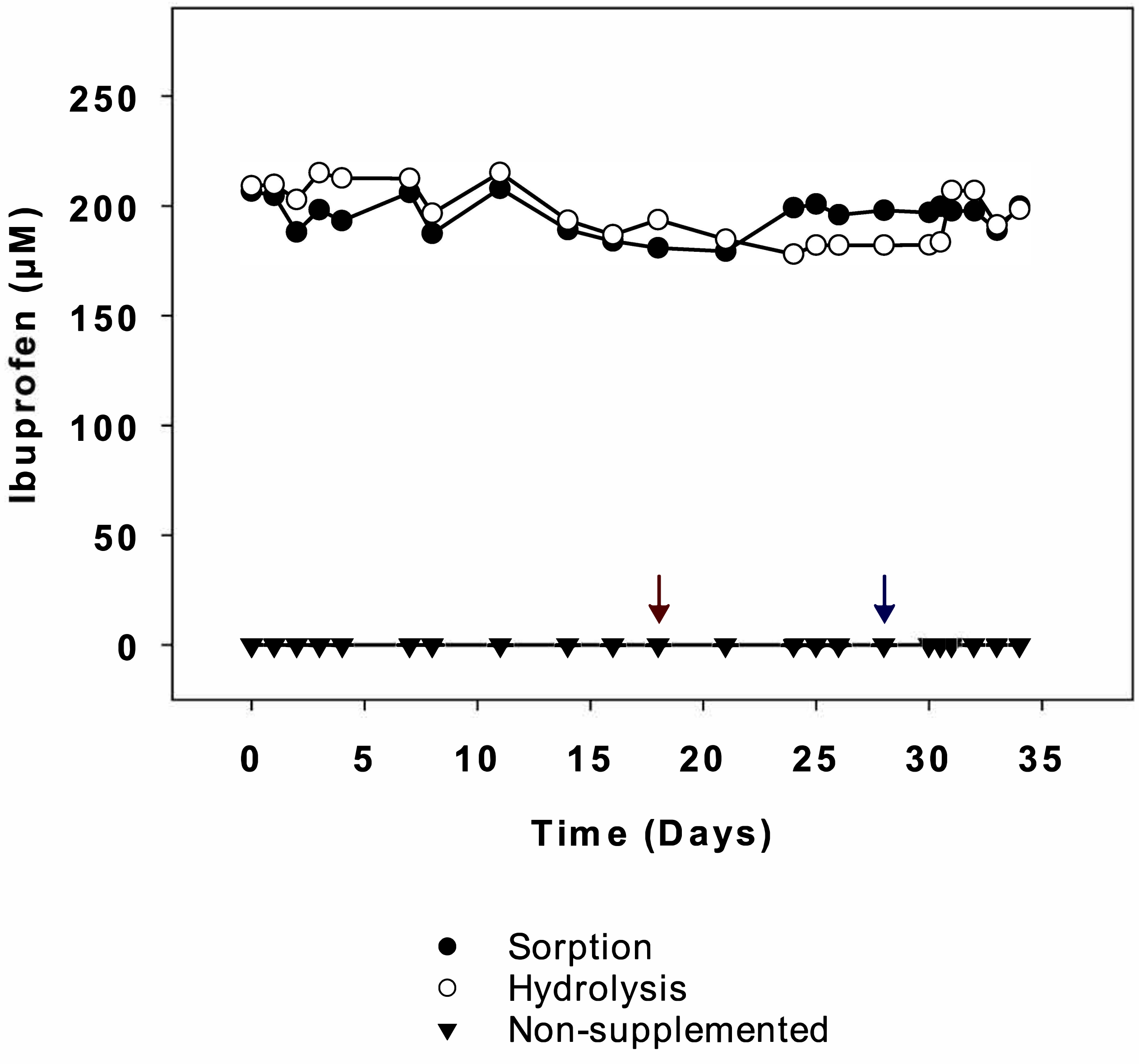

Figure S1: Control microcosms for the ibuprofen degradation experiment (Fig. 1). 'Non -supplemented' represents the unamended sediment. The abiotic controls 'Sorption' and 'Hydrolysis' contained autoclaved sediment and river water, respectively, and were amended with 200 µM ibuprofen. Value s are the arithmetic means of three replicate incubations. Red and blue-headed arrows indicate sampling of the sediment for nucleic acids extraction after third and fifth re-spiking, respectively. Error bars indicating standard deviations are smaller than the size of the symbols and therefore not apparent.

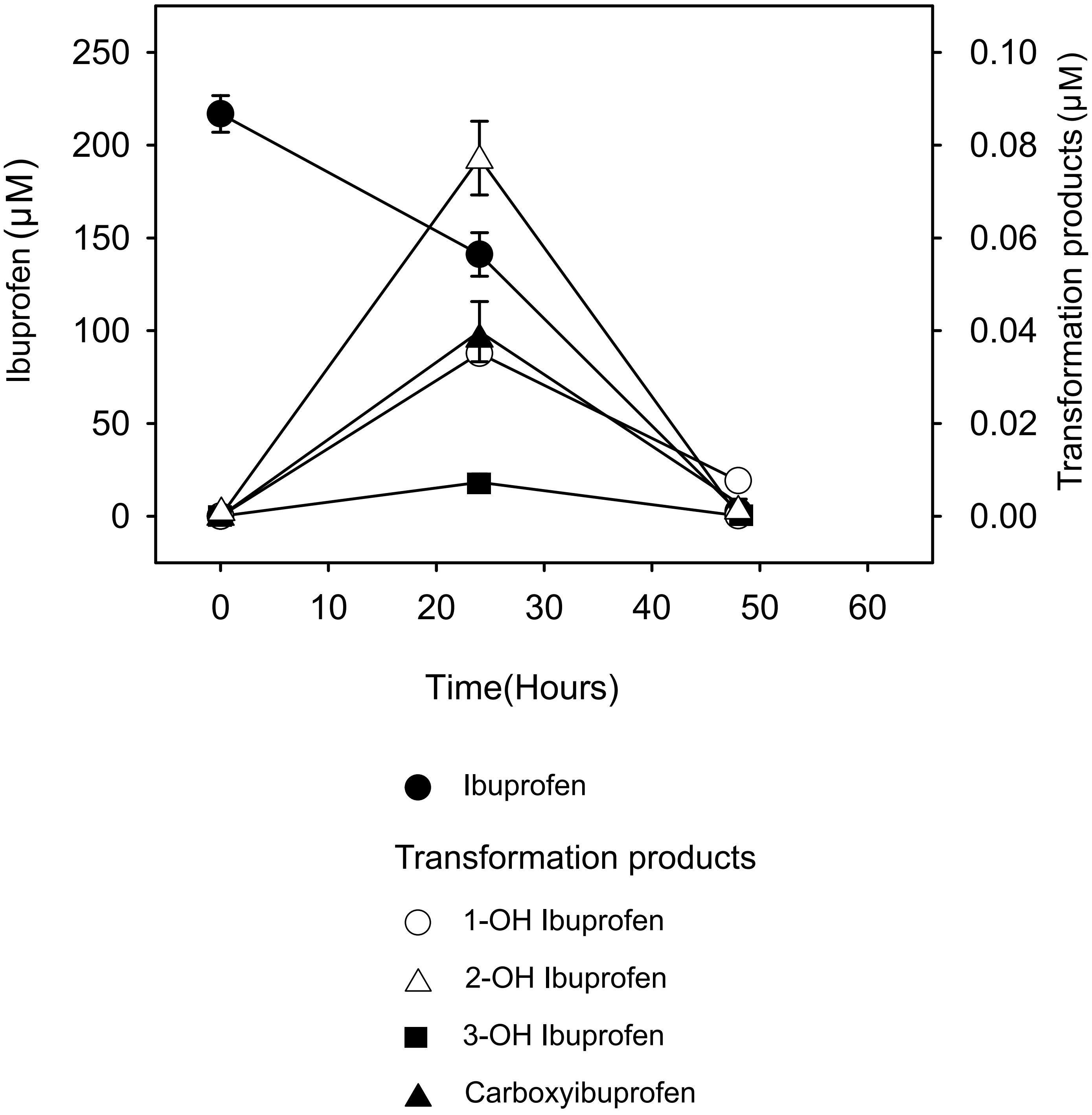

Figure S2: Ibuprofen transformation products in oxic hyporheic zone sediment microcosms amended with 200 µM of ibuprofen (4th respike; see Fig. 1 B). Values are the arithmetic mean of three replicate incu bations, and error bars indicate standard deviations. Some standard deviations are smaller than the symbol size and therefore not apparent.

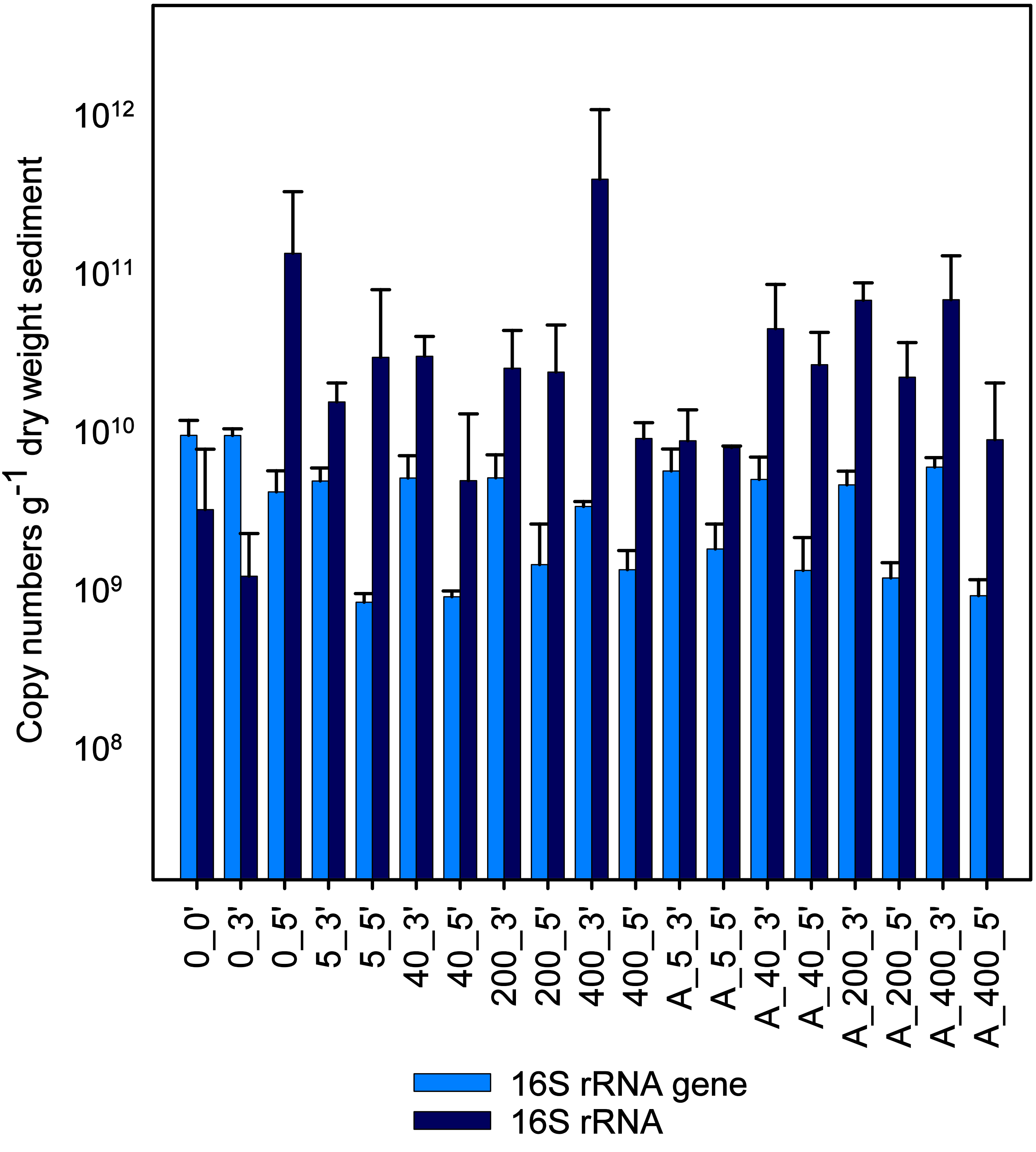

Figure S3: Copy numbers of the 16S rRNA gene and 16S rRNA detected in total bacterial community. Sample code: A, amended with 1 mM acetate and ibuprofen per feeding; 0, 5, 40, 200, and 400, indicate supplemental ibuprofen concentrations of 0 μM, 5 μM,40 μM, 200 μM and 400 μM, respectively, given per feeding; 0', 3', and 5', correspond to samples obtained at the start of the incubation, and after the third and fifth refeeding, respectively. Sampling times for unamended controls were according to those of the 400 μM ibuprofen treatment. Values are the arithmetic average of three replicates. Error bars indicate standard deviation values.

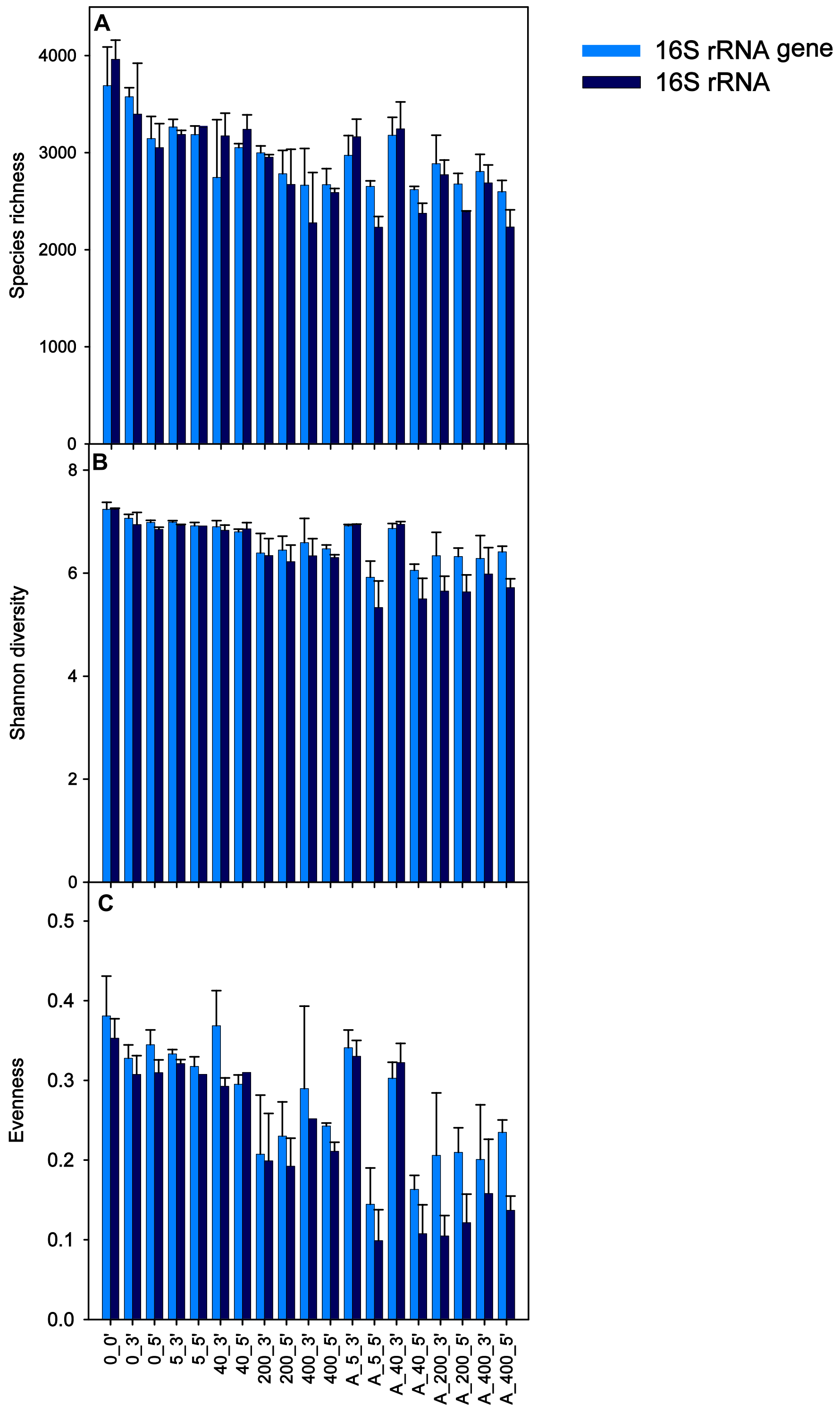

Figure S4: Alpha diversity and richness estimators of 16S rRNA gene and 16S rRNA obtained from Illumina amplicon sequencing. Sample code: A, amended with 1 mM acetate and ibuprofen per feeding; 0, 5, 40, 200, and 400, indicate supplemental ibuprofen concentrations of 0 µM, 5 µM, 40 µM, 200 µM and 400 µM, respectively, given per feeding; 0', 3', and 5', correspond to samples obtained at the start of the incubation, and after the third and fifth refeeding, respectively. Sampling times for unamended controls were according to those of the 400 µM ibuprofen treatment. Values are the arithmetic average of three replicates. Error bars indicate standard deviation values.

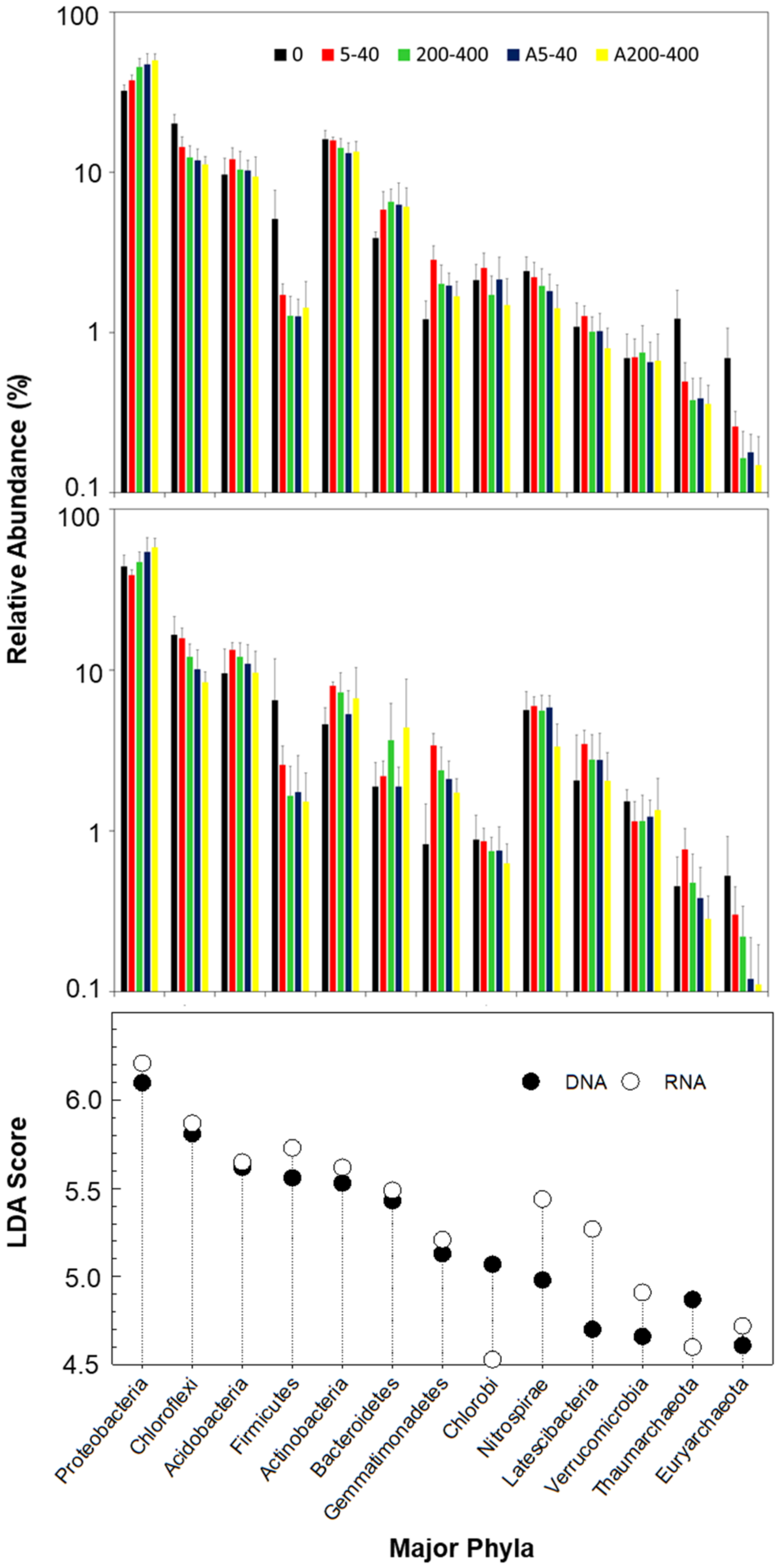

Figure S5: Relative abundance of major bacterial (greater than 1% relative abundance) and archaeal phyla on DNA (A) and RNA (cDNA, B) level, as well as corresponding LDA scores (C). Archaea are indicated by the grey box. Values and error bars represent means from all time points per treatment (see Figures. 1, S1) of up to 12 samples and standard deviation, respectively. 0, unamended controls; 5-40, 'low ibuprofen treatments' with 5 and 40 µM ibuprofen; 200-400, 'high ibuprofen treatments' with 200 and 400 µM ibuprofen; A5-40, 'low ibuprofen treatments' with supplemental 1 mM acetate; A200-400, 'high ibuprofen treatments' with supplemental 1 mM acetate. Filled and open circles, DNA and RNA (cDNA) level, respectively.

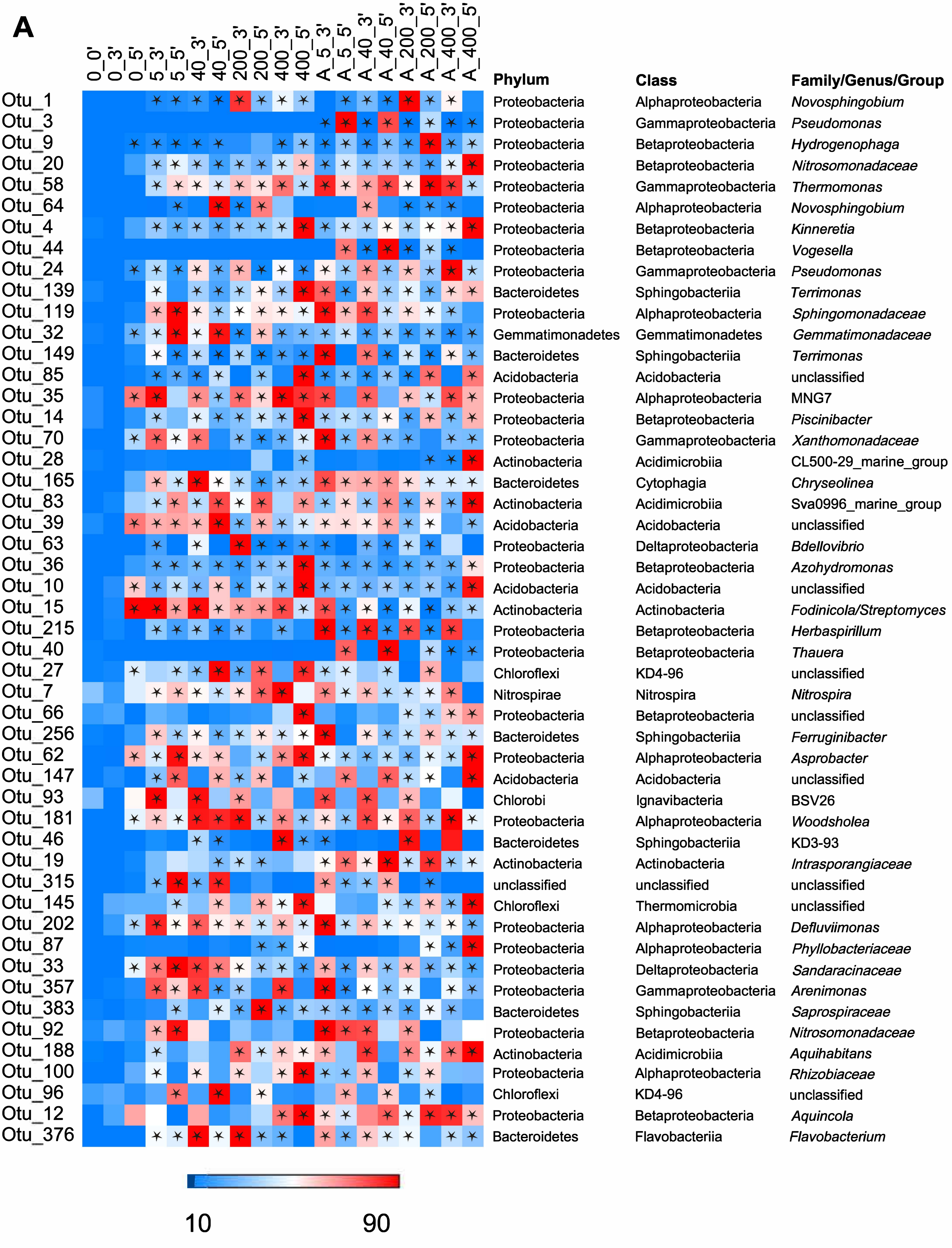
A
0_0'
0_3'
0_5'
5_3'
5_5'
40_3'
40_5'
200_3'
200_5'
400_3'
400_5'
A_5_3'
A_5_5'
A_40_3'
A_40_5'
A_200_3'
A_200_5'
A_400_3'
A_400_5'
Phylum
Class
Family/Genus/Group
Otu_1 Proteobacteria Alphaproteobacteria Novosphingobium
Otu_3 Proteobacteria Gammaproteobacteria Pseudomonas
Otu_9 Proteobacteria Betaproteobacteria Hydrogenophaga
Otu_20 Proteobacteria Betaproteobacteria Nitrosomonadaceae
Otu_58 Proteobacteria Gammaproteobacteria Thermomonas
Otu_64 Proteobacteria Alphaproteobacteria Novosphingobium
Otu_4 Proteobacteria Betaproteobacteria Kinneretia
Otu_44 Proteobacteria Betaproteobacteria Vogesella
Otu_24 Proteobacteria Gammaproteobacteria Pseudomonas
Otu_139 Bacteroidetes Sphingobacteriia Terrimonas
Otu_119 Proteobacteria Alphaproteobacteria Sphingomonadaceae
Otu_32 Gemmatimonadetes Gemmatimonadetes Gemmatimonadaceae
Otu_149 Bacteroidetes Sphingobacteriia Terrimonas
Otu_85 Acidobacteria Acidobacteria unclassified
Otu_35 Proteobacteria Alphaproteobacteria MNG7
Otu_14 Proteobacteria Betaproteobacteria Piscinibacter
Otu_70 Proteobacteria Gammaproteobacteria Xanthomonadaceae
Otu_28 Actinobacteria Acidimicrobiia CL500-29_marine_group
Otu_165 Bacteroidetes Cytophagia Chryseolinea
Otu_83 Actinobacteria Acidimicrobiia Sva0996_marine_group
Otu_39 Acidobacteria Acidobacteria unclassified
Otu_63 Proteobacteria Deltaproteobacteria Bdellovibrio
Otu_36 Proteobacteria Betaproteobacteria Azohydromonas
Otu_10 Acidobacteria Acidobacteria unclassified
Otu_15 Actinobacteria Actinobacteria Fodinicola/Streptomyces
Otu_215 Proteobacteria Betaproteobacteria Herbaspirillum
Otu_40 Proteobacteria Betaproteobacteria Thauera
Otu_27 Chloroflexi KD4-96 unclassified
Otu_7 Nitrospirae Nitrospira Nitrospira
Otu_66 Proteobacteria Betaproteobacteria unclassified
Otu_256 Bacteroidetes Sphingobacteriia Ferruginibacter
Otu_62 Proteobacteria Alphaproteobacteria Asprobacter
Otu_147 Acidobacteria Acidobacteria unclassified
Otu_93 Chlorobi Ignavibacteria BSV26
Otu_181 Proteobacteria Alphaproteobacteria Woodsholea
Otu_46 Bacteroidetes Sphingobacteriia KD3-93
Otu_19 Actinobacteria Actinobacteria Intrasporangiaceae
Otu_315 unclassified unclassified unclassified
Otu_145 Chloroflexi Thermomicrobia unclassified
Otu_202 Proteobacteria Alphaproteobacteria Defluviimonas
Otu_87 Proteobacteria Alphaproteobacteria Phyllobacteriaceae
Otu_33 Proteobacteria Deltaproteobacteria Sandaracinaceae
Otu_357 Proteobacteria Gammaproteobacteria Arenimonas
Otu_383 Bacteroidetes Sphingobacteriia Saprospiraceae
Otu_92 Proteobacteria Betaproteobacteria Nitrosomonadaceae
Otu_188 Actinobacteria Acidimicrobiia Aquihabitans
Otu_100 Proteobacteria Alphaproteobacteria Rhizobiaceae
Otu_96 Chloroflexi KD4-96 unclassified
Otu_12 Proteobacteria Betaproteobacteria Aquincola
Otu_376 Bacteroidetes Flavobacteriia Flavobacterium
10
90

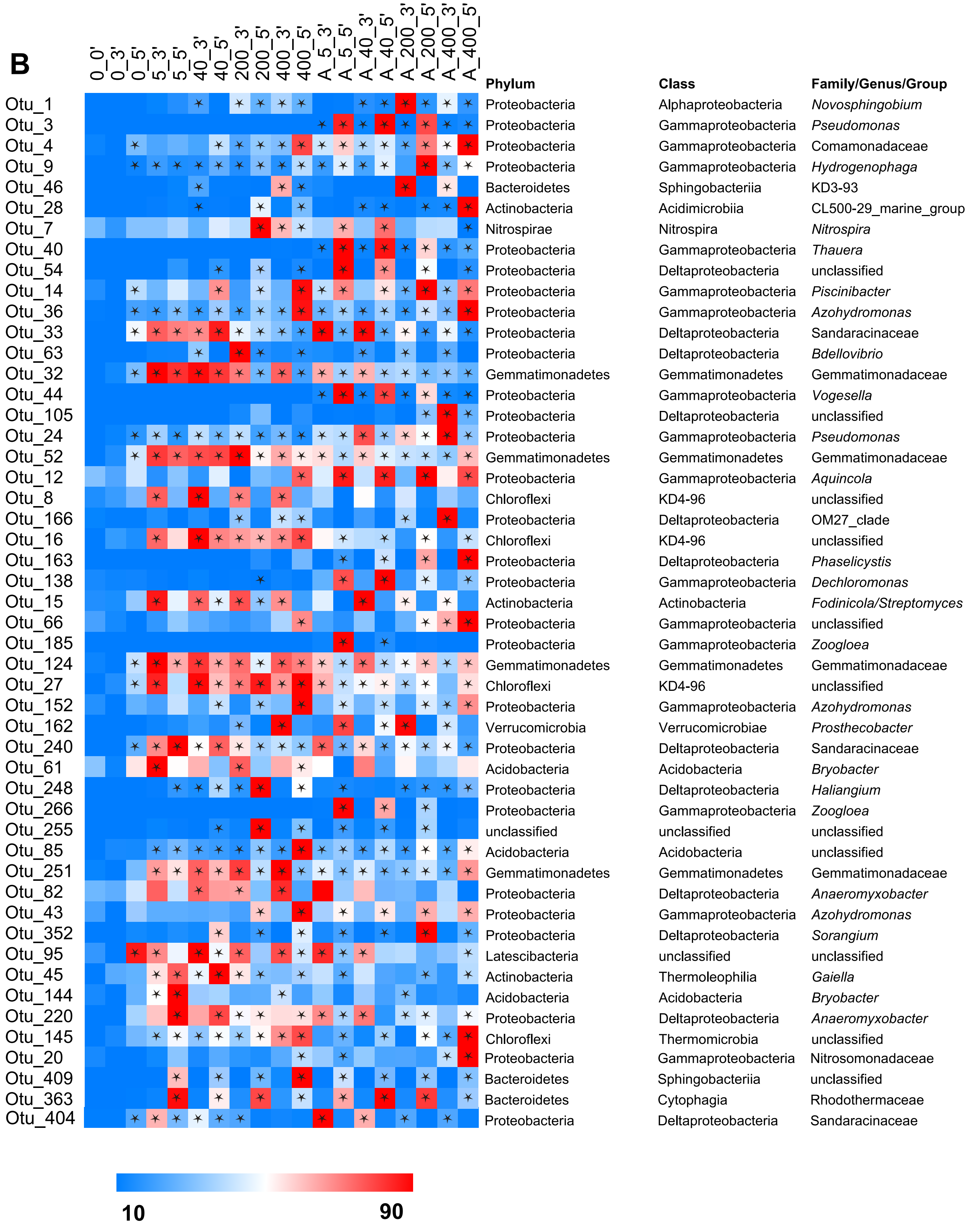

Figure S6: Heat map of LEfSe identified top 50 most differentially abundant taxa based on 16S rRNA gene (A) and 16S rRNA (cDNA; B) amplicon analysis after the third and fifth refeeding (Figure. 1). OTUs are sorted according to the difference in their mean read counts in ibuprofen amended and unamended samples. OTUs with a linear discriminant analysis score of ≥ 4 and their phylogenetic affiliation are shown. The color code reflects median, as well as upper and lower quantiles of read counts normalized to the total number of reads per OTU from all samples. Sample code: A, amended with 1 mM acetate and ibuprofen per feeding; 0, 5, 40, 200, and 400 indicate supplemental ibuprofen concentrations in µM given per feeding; 0’, 3’, and 5’ correspond to samples obtained at the start of the incubation, and after the third and fifth refeeding, respectively. Sampling times for unamended controls were according to those of the 400 µM ibuprofen treatment. *, Samples with significant differential relative abundance compared to the start of the incubation based on DESeq2 (adj $p < 0.1$).

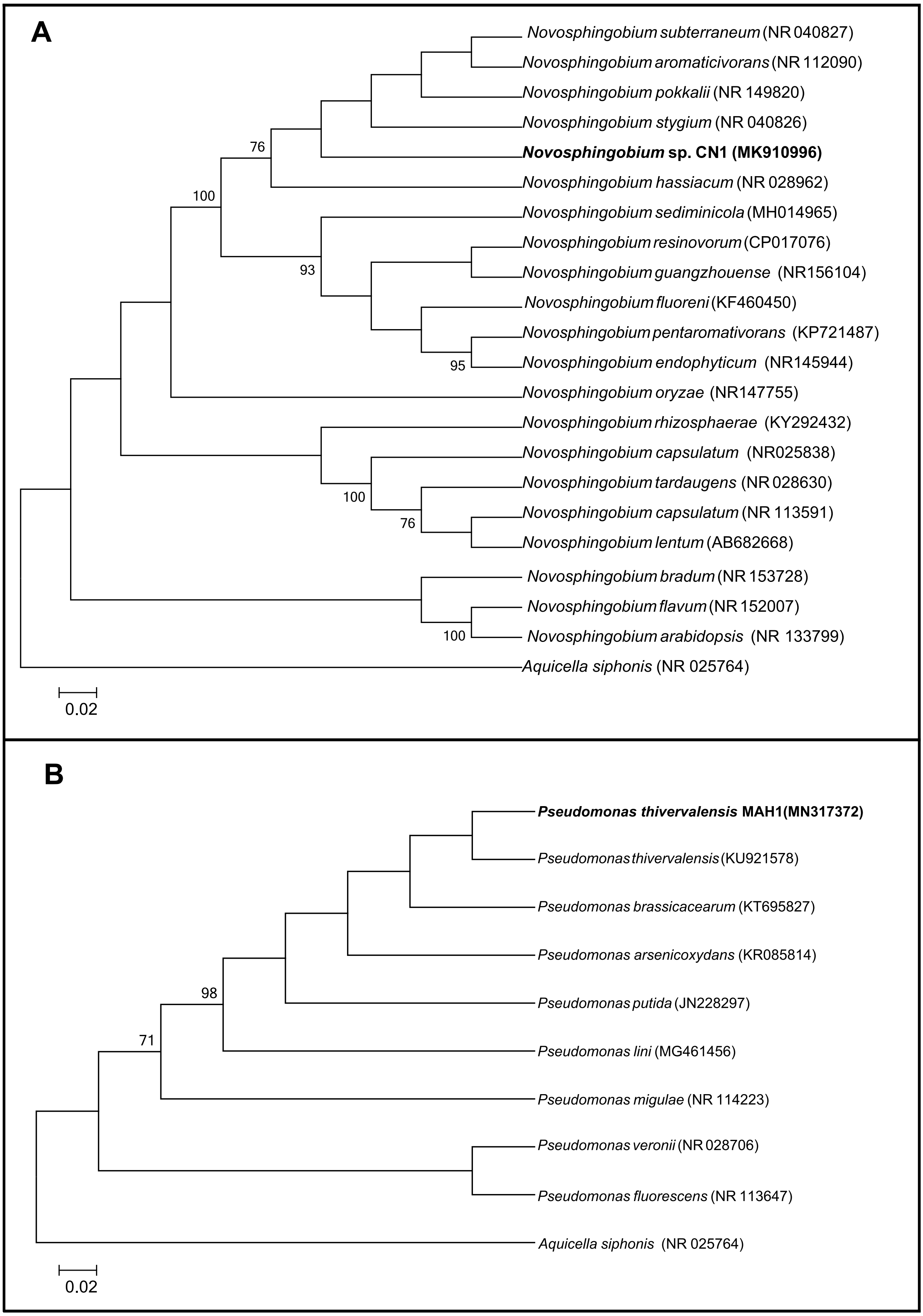

Figure S7: Phylogenetic tree reconstructed with the neighbour-joining method based on 16S rRNA gene sequences of strain CN1 and other *Novosphingobium* species (panel A) and strain MAH1 and other *Pseudomonas* species (panel B), showing their position among phylogenetic neighbours. *Aquicella siphonis* strain SGT-108 was used as an outgroup. Bootstrap values (based on 1000 replicati ons) above 70 % are shown at branch nodes.

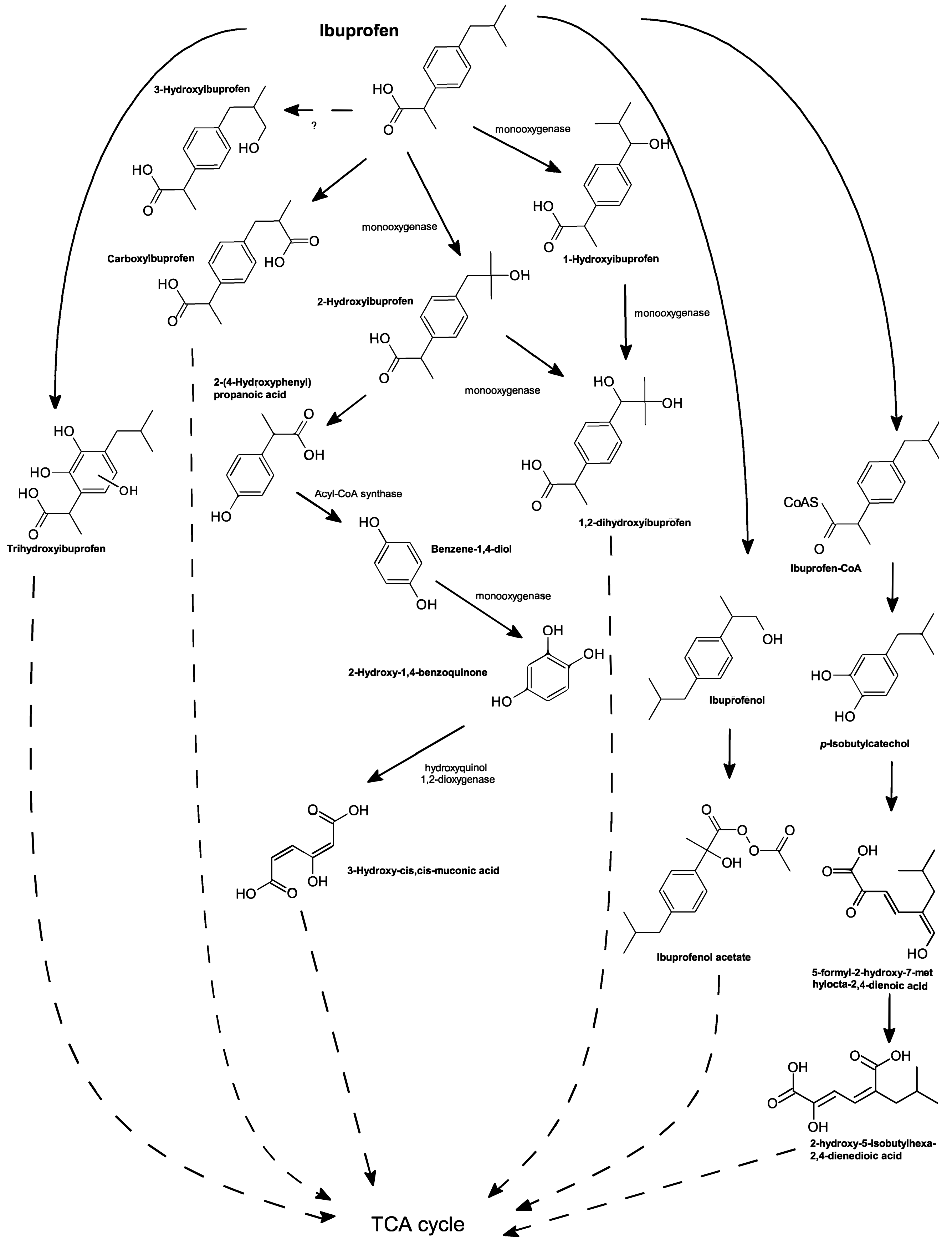

Figure. S8: Hypothetical ibuprofen degradation pathways in oxic hyporheic zone sediments based on transformation products identified in this study and previous studies as compiled in Żur et al. [48].

Table S1. Sequences and annealing temperature of primer sets targeting representative ibuprofen-responsive taxa (400 μM ibuprofen treatment; Figure 4, Table S2). The primers were used to amplify the 16S rRNA gene and corresponding 16S rRNA transcripts (cDNA).

Target taxa (representative OTUs from amplicon libraries included in the groups targeted)	Primer name	Primer sequence	Annealing temperature	Reference
Actinobacteria (OTU 28)	HGC236F	5'-GCG GCC TAT CAG CTT GTT-3'	55.5°C	Warnecke et al. [33]
	HGC664R	5'-AGG AAT TCC AGT CTC CCC-3'		
Sphingomonadaceae (OTU 1)	SPF	5'-AAG TCA GAG GTG AAA GCC CG-3'	58°C	This study
	SPR	5'-TTG TCC AGT CAG TCG CCT TC-3'		
Acidobacteria Subgroup17/Unclassified (OTU 39)	S17F	5'-TTG TCC AGT CAG TCG CCT TC-3'	58°C	This study
	S17R	5'-TCA AGC CTG CTA GTT TCC CG-3'		
Hydrogenophaga (OTU 9)	HYF	5'-GCC TTC GGG TTG TAA ACT GC-3'	58°C	This study
	HYR	5'-AAA CGC CAT TCC CAG GTT GA-3'		
Fodinicola (OTU 15)	FDF	5'-CCT CTT TCA GCA GGG ACG AA-3'	58°C	This study
	FDR	5'-AAG TCT GCC CGT ATC GAG TG-3'		
Chloroflexi/Unclassified (OTU 16)	CHF	5'-TCG GGA ATT TTG CGC AAT GG-3'	58°C	This study
	CHR	5'-CCT GCC TTC GAG TCG ATC AG-3'		
Gemmatimonadetes/Unclassified (OTU 32)	GMF	5'-AAA CCA CTG TCG GAA GGG AC-3'	58°C	This study
	GMR	5'-CGA GCC TGG CAG TCT AGA AG-3'		

Table S2. Classification of bacterial OTUs enriched by ibuprofen relative to unamended controls sampled at the same time point, and closest cultured relatives of OTU representative 16S rRNA gene sequences. Significant (p-adj < 0.05) Log2-fold change > 0 are reported as determined by Deseq2. IBU40 and IBU400, ibuprofen amendment with 40 and 400 µM ibuprofen, respectively. IBA40 and IBA400, ibuprofen amendment with 40 and 400 µM ibuprofen, respectively, together with 1 mM acetate.

Phylum/Subphylum-level	Genus-level (OTU No.)	Closest cultured relative	Acc. No. [a]	[%] [b]	Log2-fold change							
					16S rRNA genes				16S RNA			
					- Acetate		+ Acetate		- Acetate		+ Acetate	
					IBU40	IBU400	IBA40	IBA400	IBU40	IBU400	IBA40	IBA400
Acidobacteria	*Bryobacter* (144)	*Bryobacter aggregatus*	AM 887762	92	2.1	-- [c]	--	--	2.1	2.3	1.8	--
	Bryobacter (61)*d	*Paludibaculum fermentans*	NR_134120	93	--	--	--	--	1	2.0	1.8	1.4
	Bryobacter (115)	*Bryobacter aggregatus*	AM887762	90	--	--	--	--	2.1	--	1.5	--
	Bryobacter (562)	*Bryobacter aggregatus*	AM887762	90	--	--	--	--	--	2.5	--	2.5
	Uncultured subgroup_3 (901)	*Paludibaculum fermentans*	NR_134120	93	--	--	--	--	--	3.3	2.7	3.8
	Uncultured subgroup_3 (55)	*Paludibaculum fermentans*	NR_134120	94	--	--	--	--	--	1.5	--	3.3
	Uncultured subgroup_3 (516)	*Paludibaculum fermentans*	NR_134120	93	--	--	--	--	--	2.2	--	1.5
	Blastocatella (126)	*Blastocatella fastidiosa*	NR_118350	95	1.3	--	--	--	--	--	--	--
	Uncultured subgroup_6 (85)	*Vicinamibacter silvestris*	CP011998	87	3.9	--	2.6	--	--	4.4	--	--
	Uncultured subgroup_6 (10)	*Vicinamibacter silvestris*	NR_151905	92	1.9	--	--	--	2.6	2.1	--	--
	Uncultured subgroup_6 (212)	*Vicinamibacter silvestris*	NR_151905	90	--	--	--	--	3.6	3.7	3	2.5
	Uncultured subgroup_6 (304)	*Luteitalea pratensis*	NR_156918	91	2.6	--	--	--	2.9	--	--	--
	Uncultured subgroup_6 (401)	*Luteitalea pratensis*	NR_156918	93	--	--	--	--	2.8	2.9	--	--

	Uncultured subgroup_6 (581)	*Vicinamibacter silvestris*	NR_151905	93	--	--		--	--		--	2.9		--	2.7
	Uncultured subgroup_6 (412)	*Vicinamibacter silvestris*	NR_151905	91	--	--		--	--		3.1	2.7		--	--
	Uncultured subgroup_6 (147)	*Ralstonia solanacearum*	CP011998	90	1.9	2.0		--	--		--	--		--	--
	Uncultured subgroup_6 (85)	*Ralstonia solanacearum*	CP011998	90	--	--		--	--		--	4.4		--	--
	Uncultured subgroup_17 (39)	*Vicinamibacter silvestris*	NR_151905	88	3.1	2.2		2.6	1.7		1.7	2		1.6	--
	Uncultured subgroup_22 (329)	*Anaeromyxobacter dehalogenans*	NR_074927	86	3.3	--		--	--		4.4	2.8		3.9	--
	Uncultured subgroup_22 (340)	*Thermaerobacter marianensis*	NR_074944	86	--	--		--	--		3.1	--		--	2
	Uncultured subgroup_22 (57)	*Vicinamibacter silvestris*	NR_151905	85	1.8	2		--	--		2.2	3.2		2.6	2.2
Actinobacteria	Uncultured Sva0996_marine_group (83)	*Acidimicrobium ferrooxidans*	NR_074390	92	2.1	--		--	--		2.6	--		2.2	2
	Aeromicrobium (332)	*Aeromicrobium panaciterrae*	NR_041382	100	4	--		--	--		--	--		--	--
	Marmoricola (69)	*Marmoricola pocheonensis*	NR_108597	99	1.9	--		--	--		--	--		--	--
	Nocardioides (372)	*Nocardioides intraradicalis*	NR_152019	97	2.6	2.9		2.5	--		--	--		--	--
	Nocardioides (210)	*Nocardioides sediminis*	NR_044228	100	1.9	--		--	--		2.9	--		--	--
	Nocardioides (102)	*Nocardioides islandensis*	NR_044235	96	--	1.7		1.6	1.8		--	--		--	--
	Uncultured MB-A2-108 (94)	*Kitasatospora kazusensis*	NR_041539	92	--	--		--	--		--	3.7		3.3	2.9

	Iamia (188)	*Aquihabitans daechungensis*	NR_132289	99	--	2.7		2.8	2.9		2.5	3.7		3	3.7
	Iamia (362)	*Iamia majanohamensis*	NR_041634	94	--	--		--	--		3.3	--		--	--
	Uncultured Acidimicrobiales (1265)	*Aciditerrimonas ferrireducens*	NR_112972	94	--	--		--	4.2		--	--		--	4.8
	Uncultured 480-2(627)	*Solirubrobacter phytolaccae*	NR_133858	90	--	--		--	--		3.6	--		3.5	--
	Solirubrobacter (29)	*Solirubrobacter ginsenosidimutans*	NR_108192	99	--	--		--	--		2.8	--		--	--
	Sva0996_marine_group(441)	*Acidimicrobium ferrooxidans*	NR_074390	91	--	--		--	--		2.9	--		--	--
	Flavobacterium (633)	*Flavobacterium cheniae*	NR_044198	97	--	5.3		5.4	6.7		--	--		--	--
	Flavobacterium (2394)	*Flavobacterium cheonhonense*	NR_125552	100	--	4.9		--	4.7		--	--		--	--
	Uncultured NS9_marine_group (438)	*Vicingus serpentipes*	NR_159281	87	--	3.1		--	3.6		--	--		--	4.1
	Uncultured NS9_marine_group (277)	*Phaeocystidibacter marisrubri*	NR_136475	86	--	3.2		--	3.6		--	3.1		--	3.5
	Rhodococcus (459)	*Rhodococcus koreensis*	LT838098	100	--	4.4		--	4.5		4.3	4.3		3.7	5.4
	Fodinicola (15)	*Streptomyces aomiensis*	NR_112998	97	--	1.4		--	1		1.3	1.8		1.8	1.6

	Nocardioides (405)	*Nocardioides opuntiae*	NR_133796	97	--	3.9		3.8	3.4		--	--		--	--
	Uncultured Acidimicrobiales (612)	*Ilumatobacter nonamiensis*	NR_112713	92	--	--		4.1	--		--	--		3.7	3.7
	Uncultured Actinobacteria (359)	*Longivirga aurantiaca*	NR_159886	91	--	2.8		--	2.9		--	--		--	--
	Uncultured MB-A2-108 (137)	*Streptacidiphilus hamsterleyensis*	NR_133867	90	1.7	1.8		--	--		3.3	4		3.2	--
	CL500-29_marine_group (28)	*Ilumatobacter nonamiensis*	NR_112713	92	--	--		--	2.2		--	--		1.9	2.1
	Uncultured Acidimicrobiales (569)	*Aciditerrimonas ferrireducens*	NR_112972	94	--	--		--	--		--	--		3.2	3.5
	Uncultured *Intrasporangiaceae* (19)	*Knoellia flava*	MG205530	99	--	--		1.3	1.3		--	--		--	--
	Gaiella (45)	*Gaiella occulta*	NR_118138	97	--	--		--	--		1.7	--		--	--
Armatimonadetes	Uncultured Armatimonadetes (371)	*Fimbriimonas ginsengisoli*	NR_121726	88	4.7	--		--	--		--	--		--	--
Bacteroidetes	*Chryseolinea* (165)	*Chryseolinea serpens*	NR_108511	96	4.3	4		5	4.4		--	3.4		3.6	--
	Chryseolinea (483)	*Chryseolinea serpens*	NR_108511	93	3.4	4.1		4.4	--		--	--		--	--
	Chryseolinea (231)	*Chryseolinea serpens*	NR_108511	92	2.6	2.9		3.3	2.5		--	--		--	--
	Chryseolinea (1195)				3.8	--		4.1	--		--	--		--	--
	Uncultured *Rhodothermaceae* (363)	*Natronotalea proteilytica*	NR_158101	88	4.5	--		--	--		5.1	--		--	--

	Ferruginibacter (499)	*Ferruginibacter lapsinanis*	NR_044589	96	3.9	5.5		5.4	6.9		--	--		--	--
	Ferruginibacter (378)	*Ferruginibacter yonginensis*	NR_133743	96	--	3.1		--	--		--	--		--	--
	Flavobacterium (2394)	*Flavobacterium cheonhonense*	NR_125552	100	--	6.5		--	--		--	--		--	--
	Flavobacterium (376)	*Flavobacterium inkyongense*	NR_156036	99	4.9	--		--	5.6		--	--		--	--
	Uncultured *Cytophagaceae* (451)	*Chryseolinea serpens*	NR_108511	91	4.4	--		4.5	--		--	--		--	--
	Ferruginibacter (256)	*Ferruginibacter alkalilentus*	NR_044588	97	2.5	2.6		3	3.1		--	--		--	--
	Uncultured *Saprospiraceae* (380)	*Phaeodactylibacter luteus*	NR_136808	88	--	2.9		--	3.2		--	2.3		--	--
	Uncultured *Saprospiraceae* (383)	*Portibacter lacus*	NR_113569	90	4.4	3.7		--	3.8		--	--		--	--
	Uncultured *Saprospiraceae* (472)	*Phaeodactylibacter xiamenensis*	NR_134132	88	--	4.5		3.6	3.9		--	--		--	--
	Uncultured Sphingobacteriales (711)	*Ferruginibacter profundus*	NR_148259	90	--	5.9		4.7	6.5		--	--		--	--
	Uncultured env.OPS_17 (726)	*Lentimicrobium saccharophilum*	NR_149795	87	--	6.6		--	7		--	--		--	5.7
	Uncultured env.OPS_17 (876)	*Mucilaginibacter terrae*	NR_158094	85	--	5.7		5.1	--		--	--		--	--
	Uncultured env.OPS_17 (548)	*Anseongella ginsenosidimutans*	NR_148803	85	4.1	--		--	--		4.3	--		--	--
	Uncultured KD3-93 (46)	*Geofilum rhodophaeum*	NR_158091	89	--	7.5		--	--		--	7.8		--	5.4
	Uncultured NS11-12_marine_group (616)	*Ekhidna lutea*	NR_115061	89	--	5.3		4	4.9		--	--		--	--
	PHOS-HE51 (1186)	*Owenweeksia hongkongensis*	NR_074100	88	--	5		--	5.3		--	--		--	--

	Uncultured Sphingobacteriales (409)	*Ferruginibacter profundus*	NR_148259	88	3.7	--		2.8	--		5.1	--		--	--
	Terrimonas (139)	*Terrimonas lutea*	NR_041250	98	2.7	3.5		4.3	4.6		--	--		--	--
	Terrimonas (149)	*Terrimonas soli*	NR_159891	98	--	--		4.2	4.3		--	--		--	--
	Terrimonas (253)	*Terrimonas arctica*	NR_134213	98	--	2.4		2.7	2.4		--	--		--	--
					--	--		--	--						
Cand_div_BRC1	Uncultured Candidate_div_BRC1 (542)	*Desulfomonile limimaris*	NR_025079	81	--	--		--	--		6.4	--		--	--
	Uncultured Candidate_div_BRC1 (1346)	*Desulfosalsimonas propionicica*	NR_115678	80	--	--		--	--		5.5	--		--	--
Gemmatimonadetes	Uncultured *Gemmatimonadaceae* (124)	*Gemmatimonas phototrophica*	NR_136770	91	3	3.9		3.8	3.8		4.3	4.6		4.4	3.7
	Uncultured *Gemmatimonadaceae* (32)	*Gemmatimonas phototrophica*	NR_136770	86	2.8	1.7		1.5	1.5		3.3	3.2		2.6	2.1
	Uncultured *Gemmatimonadaceae* (52)	*Gemmatimonas phototrophica*	NR_136770	85	2.2	--		--	--		2.9	2.7		2.2	1.8
	Uncultured *Gemmatimonadaceae* (251)	*Gemmatimonas aurantiaca*	NR_074708	85	4.3	4		--	--		3.9	4.5		3.2	2.7
	Uncultured *Gemmatimonadaceae* (269)	*Gemmatimonas phototrophica*	NR_136770	91	3	--		--	3.3		5	5.6		5.2	4.6
	Uncultured *Gemmatimonadaceae* (299)	*Gemmatimonas aurantiaca*	NR_074708	85	2.5	--		--	--		3.5	--		2.8	2.6
	Uncultured *Gemmatimonadaceae* (388)	*Gemmatirosa kalamazoonesis*	NR_132675	89	--	--		--	--		3	2.7		2.3	--

Chlorobi	Uncultured BSV26 (580)	*Ignavibacterium album*	NR_074698	86	4.6	--		--	--		--	--		--	--
	Uncultured BSV26 (93)	*Ignavibacterium album*	NR_074698	82	--	2.1		2.6	2		--	--		2.7	2.5
	Uncultured BSV26 (413)	*Ignavibacterium album*	NR_074698	82	--	4.2		3.6	3.9		--	--		--	--
	Uncultured OPB56 (740)	*Hippea maritima*	NR_119289	81	--	--		--	4.9		--	4.9		4.5	5.2
	Uncultured SJA-28 (284)	*Melioribacter roseus*	NR_074796	83	1.9	--		--	--		--	--		--	--
Chloroflexi	Uncultured *Caldilineaceae* (110)	*Litorilinea aerophila*	NR_132330	88	--	--		--	--		3.5	--		--	--
	Uncultured KD4-96 (27)	*Dehalogenimonas alkenigignens*	NR_109657	86	1.5	--		--	--		1.6	1.8		--	--
	Uncultured *Anaerolineaceae* (233)	*Thermanaerothrix daxensis*	NR_117865	88	2.7	--		--	--		3.9	--		2.8	2.5
	Uncultured *Anaerolineaceae* (611)	*Thermomicrobium roseum*	NR_044678	84	4.3	--		4.8	4.8		4.5	--		3.9	--
	Uncultured *Anaerolineaceae* (628)	*Bellilinea caldifistulae*	NR_041354	84	3.4	--		--	--		3.1	--		--	--
	Unclassified Gitt-GS-136 (127)	*Hydrogenispora ethanolica*	NR_125455	85	1.8	--		--	--		2.4	--		--	--
	Uncultured KD4-96 (96)	*Hydrogenispora ethanolica*	NR_125455	85	1.6	--		--	--		2.3	2.1		--	--
	Unclassified JG30-KF-CM45 (145)	*Sphaerobacter thermophilus*	NR_074379	88	2.1	2		--	2.2		2.8	--		--	--
	Uncultured KD4-96 (8)	*Dehalogenimonas lykanthroporepellens*	NR_074337	86	--	--		--	--		--	1.5		--	--
	Uncultured KD4-96 (16)	*Dehalogenimonas alkenigignens*	JQ994267	86	--	--		--	--		1.2	1.3		--	1.3

Latescibacteria	Uncultured Latescibacteria (95)	*Desulfonatronum thiosulfatophilum*	NR_116694	81	1.8	2.3		--	--		5	4.6		4.1	3.3
	Uncultured Latescibacteria (154)	*Acidicapsa acidisoli*	NR_148580	88	--	4.6		4.8	4.3		4.8	5.4		4.8	4.3
	Uncultured Latescibacteria (308)	*Syntrophorhabdus aromaticivorans*	NR_041306	84	2.6	--		--	--		2.6	--		--	--
	Uncultured Latescibacteria (326)	*Desulfopila inferna*	NR_115066	83	--	--		--	--		4.2	4.1		3.9	3.3
	Uncultured Latescibacteria (342)	*Desulfobulbus mediterraneus*	NR_025150	84	--	--		--	--		2.1	--		2.3	--
	Uncultured Latescibacteria (453)	*Desulfobulbus oligotrophicus*	NR_156089	85	--	--		--	--		3	2.8		2.4	--
	Uncultured Latescibacteria (503)	*Pseudomaricurvus alkylphenolicus*	NR_114390	81	--	--		--	--		3.9	4.2		4	3.5
	Uncultured Latescibacteria (534)	*Thermoanaerobaculum aquaticum*	NR_109681	83	--	--		--	--		4.4	--		3	--
	Uncultured Latescibacteria (706)	*Desulfotalea psychrophila*	NR_028729	83	--	--		--	--		3.1	3.8		3.6	3.3
	Uncultured Latescibacteria (732)	*Syntrophus gentianae*	NR_029295	82	--	--		--	--		4.3	3.9		4.5	3.9
	Uncultured Latescibacteria (864)	*Desulfobulbus oligotrophicus*	NR_156089	85	--	--		--	--		4.1	3.6		3.9	
	Uncultured Latescibacteria (422)	*Desulfotalea psychrophile*	NR_028729	82	--	--		--	--		3.7	3.9		3.2	3.2
	Uncultured Latescibacteria (1054)	*Desulfotalea psychrophila*	NR_028729	83	--	--		--	--		5.5	--		--	--
Nitrospirae	*Nitrospira* (7)	*Nitrospira moscoviensis*	NR_029287	98	1.4	2.7		1.9	2.4		1.4	2		--	1.6
	Nitrospira (23)	*Nitrospira moscoviensis*	NR_029287	94	--	2.3		2.3	2.1		1.6	--		--	--
	Nitrospira (26)	*Nitrospira lenta*	NR_148573	100	--	--		2.4	2.4		--	--		--	--

Alphaproteobacteria	*Sphingobium* (17)	*Sphingobium czechense*	NR_118306	99	3.7	--		--	--		--	--		--	5.4
	Hyphomicrobium (60)	*Hyphomicrobium facile*	NR_027610	98	1.6	1.8		--	2		--	--		--	--
	Hyphomicrobium (347)	*Hyphomicrobium facile*	NR_027610	96	--	--		--	--		5.2	4.8		4	4.9
	Hirschia (62)	*Asprobacter aquaticus*	NR_159164		2.4	2.9		2.1	2.5		--	--		--	--
	Novosphingobium (64)	*Novosphingobium fuchskuhlense*	NR_118270	99	6.1	--		6.1	3.3		4.4	--		4.3	--
	Pedomicrobium (84)	*Pedomicrobium manganicum*	NR_104841	96	1.7	1.8		--	2		--	--		--	2.3
	Uncultured Rhizobiales (335)	*Blastochloris viridis*	NR_117911	95	2.1	2.6		--	2.9		--	--		--	--
	Defluviimonas (202)	*Defluviimonas aestuarii*	NR_118305	98	2.2	2.8		2.8	2.6		--	--		--	--
	Hyphomicrobium (129)	*Hyphomicrobium aestuarii*	NR_104954	96	2.1	--		--	--		2.5	--		--	1.7
	Uncultured AKYH478 (645)	*Lacibacterium aquatile*	NR_125556	94	--	--		--	--		--	5		--	--
	Uncultured F0723 (1059)	*Phreatobacter stygius*	NR_158009	98	--	--		--	--		--	6.2		--	--
	Uncultured MNG7 (35)	*Nordella oligomobilis*	NR_114615	96		2.2		2	2.3		--	--		--	--
	Uncultured *Phyllobacteriaceae* (87)	*Carbophilus carboxidus*	NR_104931	91	--	--		5.9	--		--	3.4		--	--
	Uncultured *Sphingomonadaceae* (1)	*Novosphingobium chloroacetimidivorans*	NR_134105	99	--	--		4.6	6		--	5.1		3.3	6.1
	Uncultured *Sphingomonadaceae* (119)	*Sphingorhabdus wooponensis*	NR_109148	99	3.7	4.7		5.2	4.4		--	--		--	--
	Sphingopyxis (553)	*Sphingopyxis fribergensis*	NR_137271	100	--	4		4.2	5		--	--		--	--
	Woodsholea (181)	*Aquidulcibacter paucihalophilus*	NR_156862	91	5.2	5.4		5.4	6.1		2.9	--		--	2.3
	Woodsholea (183)	*Roseitalea porphyridii*	NR_156092	91	3	3.2		3.3	3.6		--	--		--	--
	Woodsholea (615)				--	3.2		--	3.1		--	--		--	--
	Uncultured *Rhizobiaceae* (100)	*Rhizobium azooxidifex*	NR_144599	99	--	3.5		3	2.7		--	--		--	--

	Uncultured AKYH478 (645)	*Lacibacterium aquatile*	NR_125556	94	--	4		--	4.2		--	4.9		--	--
	Defluviimonas (202)	*Defluviimonas aestuarii*	NR_118305	99	2.2	2.8		2.8	2.6		--	--		--	--
Deltaproteobacteria	*Bdellovibrio* (63)	*Bdellovibrio exovorus*	NR_102876	98	4.7	--		6.9	--		5.6	7.9		6.6	--
	Haliangium (479)	*Haliangium tepidum*	NR_024781	91	--	--		--	--		3.9	--		4	3.3
	Haliangium (248)	*Kofleria flava*	NR_041981	92	5	--		--	--		6.2	--		3.3	--
	Haliangium (457)	*Kofleria flava*	NR_041981	93	3.9	--		--	--		5	3.7		4.4	4.7
	Haliangium (739)	*Kofleria flava*	NR_041981	90	--	--		--	--		3.8	4		4.1	4.6
	OM27-Clade (166)	*Rhodomicrobium vannielii*	NR_074186	97	--	6.3		4.6	--		--	4.6		--	6.4
	Anaeromyxobacter (220)	*Anaeromyxobacter dehalogenans*	NR_074927	92	--	--		3.2	--		1.9	1.6		1.5	--
	Anaeromyxobacter (82)	*Anaeromyxobacter dehalogenans*	FJ190057	95	--	--		--	--		1.2	1.6		--	--
	Sandaracinus (260)	*Sandaracinus amylolyticus*	NR_118001	94	--	--		--	--		7.8	--		7.1	--
	Sandaracinus (382)	*Sandaracinus amylolyticus*	NR_118001	95	--	--		--	4.8		--	3.4		5.6	5
	Sorangium (709)	*Chondromyces lanuginosus*	NR_025345	95	--	--		--	--		4.9	3.6		4.5	4.9
	Sorangium (1348)	*Labilithrix luteola*	NR_126182	95	--	--		--	--		6.1	--		6.4	--
	Sorangium (352)	*Labilithrix luteola*	NR_126182	96	--	--		3.4	--		--	3.2		--	--
	Uncultured *Cystobacteraceae* (203)	*Vitiosangium cumulatum*	NR_156939	94	--	--		3.9	4.6		--	2.5		--	2.6
	Uncultured *Sandaracinaceae* (33)	*Sandaracinus amylolyticus*	NR_118001	91	6.3	5.8		6.8	6.5		8.3	6.6		8.4	7.8
	Uncultured *Sandaracinaceae* (404)	*Sandaracinus amylolyticus*	NR_118001	92	--	--		--	--		4.2	--		5.9	4.4

	Uncultured *Sandaracinaceae* (686)	*Sandaracinus amylolyticus*	NR_118001	89	--	--		--	--		--	3.7		3.9	4.8
	Uncultured *Sandaracinaceae* (240)	*Sandaracinus amylolyticus*	NR_118001	90	4.3	5.5		4.3	6		5.2	4.3		4.6	4.8
	Uncultured *Sandaracinaceae* (764)	*Sandaracinus amylolyticus*	NR_118001	88	--	--		--	--		6.2	--		6	--
	Uncultured Sh765B-TzT-29 (414)	*Desulfonatronum parangueonense*	NR_159236	91	--	--		--	--		4.1	--		3.9	4.3
	Uncultured δ-bacteria (105)	*Anaeromyxobacter dehalogenans*	NR_074927	89	--	--		5.5	--		--	7.5		--	--
	Uncultured δ-bacteria (54)	*Phaselicystis flava*	NR_044523	89	--	--		--	--		5.0	5.0		--	4.5
	Phaselicystis (163)	*Jahnella thaxteri*	NR_117461	95	--	--		--	--		--	--		--	8.2
Gammaproteobacteria (Betaproteobacterales)	*Hydrogenophaga* (9)	*Hydrogenophaga defluvii*	NR_029024	99	4.2	4.2		4.3	5.8		6	4.8		5.5	6.7
	Leeia (513)	*Annwoodia aquaesulis*	NR_044793	98	--	5		--	--			--		2.1	--
	Piscinibacter (14)	*Piscinibacter aquaticus*	NR_043921	99	2.5	3.5		3.1	3.5		3.7	2.3		2.4	2.8
	Rhizobacter (123)	*Rhizobacter profundi*	NR_149234	98	--	5.7		--	5.3		3	4.4		2.9	4.7
	Uncultured *Comamonadaceae* (4)	*Kinneretia asaccharophila*	NR_115151	99	1.4	2.6		2.3	3		2.2	1.8		1.8	2.7
	Uncultured *Comamonadaceae* (36)	*Azohydromonas lata*	NR_114103	98	3.1	4		3.4	4.1		4.6	4.3		3.3	4.4
	Uncultured *Comamonadaceae* (113)	*Variovorax boronicumulans*	NR_114214	98	3.1	--		4.4	5.8		--	--		--	--
	Uncultured *Comamonadaceae* (152)	*Azohydromonas lata*	NR_114103	98	--	--		--	--		2.9	2.5		2.1	2.8
	Uncultured *Comamonadaceae* (1113)	*Rhodoferax antarcticus*	NR_104835	97	3.8	--		--	5.1		--	4		--	4.1

	Uncultured *Comamonadaceae* (12)	*Aquincola tertiaricarbonis*	MH844953	99	--	1.3		--	1.6		--	1.0		--	1.0
	Azohydromonas (43)	*Zhizhongheella caldifontis*	NR_134232	98	--	2.7		--	3.1		1.8	2.1		1.9	2.8
	Uncultured *Gallionellaceae* (320)	*Sideroxydans lithotrophicus*	NR_074731	95	--	2.4		2.4	2.2		--	--		--	--
	Uncultured *Nitrosomonadaceae* (20)	*Collimonas fungivorans*	NR_028007	93	--	1.8		1.3	2		--	--		--	--
	Uncultured *Nitrosomonadaceae* (350)	*Herbaspirillum chlorophenolicum*	NR_114143	91	--	3.7		2.8	3.8		--	--		--	--
	Uncultured *Nitrosomonadaceae* (92)	*Nitrosospira multiformis*	NR_074736	92	--	--		1.1	--		--	--		--	--
	Uncultured *Oxalobacteraceae* (215)	*Herbaspirillum autotrophicum*	NR_113747	98	--	4		5.8	6.1		--	--		5.4	5.1
	Vogesella (44)	*Vogesella indigofera*	NR_040800	100	--	--		4.7	5.7		--	--		3.5	4.3
	Dechloromonas (138)	*Dechloromonas hortensis*	NR_042819	98	--	--		3.1	3.3		--	--			1.9
	Thauera (40)	*Thauera humireducens*	NR_109534	99	--	--			5.3		--	--		2.7	4.8
	Uncultured SC-I-84 (89)	*Robbsia andropogonis*	NR_104960	91	--	--			1.4		--	2.7		2.3	3.4
	Uncultured SC-I-84 (354)	*Tepidimonas thermarum*	NR_042418	91	--	--			2		--	--		--	4.1
	Uncultured β-proteobacteria (66)	*Derxia gummosa*	NR_114127	93	--	--			2		--	--		--	2.6
	Zoogloea (266)	*Zoogloea ramigera*	MK138653	100	--	--		--	--		--	--		--	3.1
	Acidiferrobacter (67)	*Sulfuricaulis limicola*	NR_147747	93		1.8		1.4	1.4		--	--		--	--
	Arenimonas (520)	*Arenimonas subflava*	NR_135888	97	5.3	--		4.7	4.7		4.5	4.7		4.1	4.1

	Arenimonas (357)	*Arenimonas subflava*	NR_135888	99		5.6		4.4	--		4.8	--		3.9	4.5
	Pseudomonas (24)	*Pseudomonas migulae*	NR_114223	99	4.5	6.2		6.8	--		3	3.1		4.6	5.4
	Uncultured *Pseudomonadaceae* (3)	*Pseudomonas linyingensis*	MG576046	100	--	--		5.0	6.6		--	--		3.4	7.8
	Thermomonas (58)	*Thermomonas carbonis*	NR_134219	97	2.5	3.6		3.4	4		--	--		--	--
	Uncultured *Xanthomonadaceae* (70)	*Dyella marensis*	NR_042691	94	--	--		2.8	1.8		--	--		--	--
	Uncultured γ-proteobacteria (452)	*Permianibacter aggregans*	NR_134131	96	--	4.7		4.2	4.5		4.5	4.4		5.6	3.9
	Uncultured γ-proteobacteria (541)	*Permianibacter aggregans*	NR_134131	93	--	--		5.3	--		5.1	--		5.9	--
	Uncultured γ-proteobacteria (505)	*Steroidobacter denitrificans*	NR_044309	90	--	4.4		3.4	3.7		--	--		--	--
	Uncultured γ-proteobacteria (715)	*Pseudomonas reidholzensis*	NR_157777	91	6.3	--		--	5.1			4.4		5.2	4.7
	Uncultured γ-proteobacteria (1758)	*Sulfuriflexus mobilis*	NR_152000	90	6.3	--		--	--		--	--		--	--
	Acidiferrobacter (196)	*Acidiferrobacter thiooxydans*	NR_114629	90	1.6	2		--	1.7		--	--		--	--
Saccharibacteria	Uncultured Saccharibacteria (980)	*Alkaliphilus metalliredigens*	NR_074633	81	4.1	--		4.9	--		--	--		--	--
					--	--		--	--		--	--		--	--
Verrucomicrobia	*Prosthecobacter* (162)	*Prosthecobacter vanneervenii*	NR_026022	97	3.5	6.8		--	6		--	3.8		--	2.9
	Verrucomicrobium (903)	*Verrucomicrobium spinosum*	NR_026266	87	--	4.3		--	--		--	3.8		--	--
	Uncultured DEV007 (1517)	*Roseibacillus ponti*	NR_041622	88	--	--		5.5			--	--		--	--
Unclassified	Uncultured bacterium (315)	*Thiomonas islandica*	NR_116394	80	6.5	--		5.1	--		--	--		--	--
	Uncultured bacterium (255)	*Negadavirga shengliensis*	NR_136439	81	--	--		--	--		4.1	6.2		--	3.7

[a] Gene bank accession number.

[b] Similarity of OTU representative 16S rRNA gene sequence to that of closest cultured relative.

[c] Non-significant differential abundance between treatment and unamended controls.

[d] Significant (p-adj < 0.1) Log2-fold change > 0 are reported as determined by Deseq2.

Table S3: Relative abundance of bacterial OTUs from the original microbial community enriched by 40 and 400 µM ibuprofen treatments at DNA and RNA levels under oxic conditions after incubation. 0, represents unamended samples and 40 and 400 correspond to samples amended with 40 and 400 µM ibuprofen, respectively. 0', 3', and 5' correspond to samples obtained at the start of the incubation, and after the third and fifth refeeding, respectively. Data represents the mean of triplicate samples in % of total rarified reads (uniform sequencing depth of 10,767 per sample) ± standard error of mean (SEM).

Phylum/Subphylum-level	Genus-level (OTU No.)	**DNA**						
		0_0‘	0_3‘	0_5‘	40_3‘	40_5‘	400_3‘	400_5‘
Acidobacteria	*Bryobacter* (144)	0.28 ± 0.05	0.04 ± 0.00	0.12 ± 0.02	0.15 ± 0.02	0.17 ± 0.02	0.10 ± 0.02	0.09 ± 0.03
	Bryobacter (61)	0.07 ± 0.03	0.05 ± 0.00	0.11 ± 0.01	0.1 ± 0.02	0.09 ± 0.00	0.12 ± 0.01	0.17 ± 0.04
	Bryobacter (115)	0.25 ± 0.06	0.03 ± 0.01	0.23 ± 0.02	0.06 ± 0.01	0.09 ± 0.02	0.07 ± 0.00	0.09 ± 0.01
	Bryobacter (562)	0.01 ± 0.00	0.01 ± 0.01	0.01 ± 0.00	0.01 ± 0.00	0.02 ± 0.01	0.01 ± 0.01	0.05 ± 0.02
	Uncultured subgroup_3 (901)	0.03 ± 0.00	0.01 ± 0.00	0.01 ± 0.00	0.01 ± 0.00	0.01 ± 0.01	0.00 ± 0.00	0.02 ± 0.01
	Uncultured subgroup_3 (55)	0.00 ± 0.00	0.11 ± 0.01	0.26 ± 0.05	0.07 ± 0.02	0.14 ± 0.03	0.08 ± 0.02	0.18 ± 0.02
	Uncultured subgroup_3 (516)	0.01 ± 0.01	0.01 ± 0.00	0.03 ± 0.01	0.01 ± 0.00	0.03 ± 0.01	0.01 ± 0.00	0.02 ± 0.00
	Blastocatella (126)	0.16 ± 0.02	0.17 ± 0.00	0.27 ± 0.01	0.16 ± 0.01	0.45 ± 0.04	0.19 ± 0.01	0.61 ± 0.12
	Uncultured subgroup_6 (85)	0.05 ± 0.01	0.02 ± 0.01	0.05 ± 0.01	0.13 ± 0.02	0.34 ± 0.12	0.09 ± 0.03	1.09 ± 0.43
	Uncultured subgroup_6 (10)	0.01 ± 0.00	0.23 ± 0.05	0.86 ± 0.12	0.48 ± 0.03	0.92 ± 0.04	0.52 ± 0.01	1.39 ± 0.22
	Uncultured subgroup_6 (212)	0.21 ± 0.03	0.03 ± 0.01	0.11 ± 0.01	0.09 ± 0.01	0.11 ± 0.02	0.06 ± 0.00	0.11 ± 0.01
	Uncultured subgroup_6 (304)	0.00 ± 0.00	0.01 ± 0.00	0.04 ± 0.00	0.08 ± 0.00	0.10 ± 0.01	0.05 ± 0.02	0.08 ± 0.01
	Uncultured subgroup_6 (401)	0.01 ± 0.01	0.04 ± 0.01	0.04 ± 0.00	0.07 ± 0.02	0.07 ± 0.02	0.09 ± 0.02	0.12 ± 0.02
	Uncultured subgroup_6 (581)	0.03 ± 0.01	0.03 ± 0.00	0.1 ± 0.02	0.02 ± 0.01	0.05 ± 0.01	0.01 ± 0.00	0.03 ± 0.00

	Uncultured subgroup_6 (412)	0.02 ± 0.00	0.04 ± 0.01	0.09 ± 0.03	0.08 ± 0.02	0.15 ± 0.02	0.08 ± 0.02	0.05 ± 0.02
	Uncultured subgroup_6 (147)	0.04 ± 0.00	0.06 ± 0.01	0.05 ± 0.01	0.11 ± 0.02	0.23 ± 0.05	0.10 ± 0.01	0.17 ± 0.02
	Uncultured subgroup_6 (85)	0.05 ± 0.01	0.02 ± 0.01	0.05 ± 0.01	0.13 ± 0.02	0.34 ± 0.12	0.09 ± 0.03	1.09 ± 0.43
	Uncultured subgroup_17 (39)	0.00 ± 0.00	0.09 ± 0.02	0.70 ± 0.06	0.54 ± 0.07	0.89 ± 0.09	0.42 ± 0.02	0.60 ± 0.17
	Uncultured subgroup_22 (329)	0.01 ± 0.01	0.01 ± 0.02	0.06 ± 0.01	0.06 ± 0.01	0.11 ± 0.01	0.04 ± 0.02	0.03 ± 0.01
	Uncultured subgroup_22 (340)	0.01 ± 0.01	0.01 ± 0.01	0.13 ± 0.01	0.03 ± 0.01	0.05 ± 0.00	0.04 ± 0.00	0.02 ± 0.01
	Uncultured subgroup_22 (57)	0.34 ± 0.03	0.04 ± 0.00	0.35 ± 0.06	0.17 ± 0.03	0.17 ± 0.05	0.17 ± 0.01	0.25 ± 0.04
Actinobacteria	Uncultured Sva0996_marine_group (83)	0.12 ± 0.01	0.1 ± 0.00	0.20 ± 0.02	0.24 ± 0.05	0.46 ± 0.03	0.22 ± 0.05	0.38 ± 0.06
	Aeromicrobium (332)	0.04 ± 0.01	0.00 ± 0.00	0.02 ± 0.01	0.01 ± 0.00	0.11 ± 0.07	0.01 ± 0.01	0.25 ± 0.13
	Marmoricola (69)	0.31 ± 0.09	0.05 ± 0.01	0.04 ± 0.02	0.20 ± 0.02	0.22 ± 0.05	0.14 ± 0.04	0.20 ± 0.10
	Nocardioides (372)	0.00 ± 0.00	0.02 ± 0.00	0.05 ± 0.02	0.09 ± 0.01	0.13 ± 0.02	0.14 ± 0.01	0.10 ± 0.02
	Nocardioides (210)	0.07 ± 0.02	0.04 ± 0.01	0.02 ± 0.00	0.15 ± 0.02	0.15 ± 0.03	0.14 ± 0.05	0.16 ± 0.09
	Nocardioides (102)	0.07 ± 0.01	0.15 ± 0.01	0.23 ± 0.00	0.39 ± 0.02	0.21 ± 0.01	0.46 ± 0.01	0.28 ± 0.09
	Uncultured MB-A2-108 (94)	0.22 ± 0.03	0.24 ± 0.07	0.39 ± 0.03	0.36 ± 0.01	0.41 ± 0.02	0.30 ± 0.04	0.21 ± 0.00
	Iamia (188)	0.15 ± 0.03	0.03 ± 0.01	0.05 ± 0.01	0.10 ± 0.01	0.08 ± 0.01	0.18 ± 0.03	0.15 ± 0.02
	Iamia (362)	0.06 ± 0.00	0.04 ± 0.01	0.02 ± 0.00	0.06 ± 0.02	0.13 ± 0.04	0.04 ± 0.01	0.08 ± 0.01
	Uncultured Acidimicrobiales (1265)	0.05 ± 0.01	0.00 ± 0.00	0.00 ± 0.00	0.02 ± 0.01	0.00 ± 0.00	0.03 ± 0.01	0.01 ± 0.00
	Uncultured 480-2(627)	0.00 ± 0.00	0.03 ± 0.01	0.03 ± 0.01	0.04 ± 0.01	0.07 ± 0.01	0.03 ± 0.00	0.05 ± 0.01
	Solirubrobacter (29)	0.05 ± 0.02	0.04 ± 0.02	0.03 ± 0.01	0.03 ± 0.01	0.04 ± 0.01	0.03 ± 0.00	0.04 ± 0.00
	Sva0996_marine_group(441)	0.00 ± 0.00	0.00 ± 0.00	0.04 ± 0.00	0.04 ± 0.01	0.04 ± 0.01	0.03 ± 0.01	0.04 ± 0.00
	Flavobacterium (633)	0.02 ± 0.01	0.00 ± 0.00	0.00 ± 0.00	0.11 ± 0.04	0.01 ± 0.00	0.18 ± 0.14	0.00 ± 0.00
	Flavobacterium (2394)	0.01 ± 0.00	0.00 ± 0.00	0.00 ± 0.00	0.00 ± 0.00	0.00 ± 0.01	0.12 ± 0.09	0.00 ± 0.00
	Uncultured NS9_marine_group (438)	0.06 ± 0.01	0.03 ± 0.01	0.05 ± 0.01	0.06 ± 0.03	0.01 ± 0.00	0.23 ± 0.07	0.08 ± 0.04
	Uncultured NS9_marine_group (277)	0.02 ± 0.00	0.02 ± 0.01	0.04 ± 0.02	0.08 ± 0.01	0.06 ± 0.00	0.24 ± 0.09	0.13 ± 0.03

	Rhodococcus (459)	0.00 ± 0.00	0.00 ± 0.00	0.08 ± 0.01	0.01 ± 0.01	0.04 ± 0.02	0.04 ± 0.01	0.04 ± 0.01
	Fodinicola (15)	0.06 ± 0.02	0.52 ± 0.08	1.25 ± 0.07	1.26 ± 0.17	0.97 ± 0.07	1.31 ± 0.18	0.76 ± 0.11
	Nocardioides (405)	0.00 ± 0.00	0.01 ± 0.00	0.10 ± 0.02	0.12 ± 0.04	0.06 ± 0.01	0.13 ± 0.01	0.12 ± 0.01
	Uncultured Acidimicrobiales (612)	0.00 ± 0.00	0.00 ± 0.00	0.05 ± 0.01	0.03 ± 0.01	0.03 ± 0.01	0.03 ± 0.00	0.04 ± 0.01
	Uncultured Actinobacteria (359)	0.00 ± 0.00	0.01 ± 0.01	0.07 ± 0.01	0.13 ± 0.02	0.05 ± 0.01	0.11 ± 0.01	0.08 ± 0.01
	Uncultured MB-A2-108 (137)	0.11 ± 0.02	0.09 ± 0.01	0.41 ± 0.06	0.24 ± 0.01	0.32 ± 0.02	0.31 ± 0.05	0.20 ± 0.03
	CL500-29_marine_group (28)	0.26 ± 0.04	0.04 ± 0.01	0.07 ± 0.03	0.07 ± 0.02	0.09 ± 0.01	0.09 ± 0.01	0.57 ± 0.04
	Uncultured Acidimicrobiales (569)	0.04 ± 0.00	0.05 ± 0.00	0.01 ± 0.00	0.08 ± 0.03	0.02 ± 0.00	0.02 ± 0.01	0.01 ± 0.01
	Uncultured *Intrasporangiaceae* (19)	0.47 ± 0.03	0.12 ± 0.01	0.18 ± 0.02	0.24 ± 0.03	0.22 ± 0.02	0.24 ± 0.03	0.22 ± 0.06
	Gaiella (45)	0.00 ± 0.00	0.45 ± 0.07	0.30 ± 0.02	0.26 ± 0.01	0.47 ± 0.02	0.22 ± 0.01	0.29 ± 0.02
Armatimonadetes	Uncultured Armatimonadetes (371)	0.17 ± 0.06	0.00 ± 0.00	0.00 ± 0.00	0.01 ± 0.01	0.07 ± 0.03	0.01 ± 0.00	0.33 ± 0.08
Bacteroidetes	*Chryseolinea* (165)	0.10 ± 0.02	0.01 ± 0.00	0.08 ± 0.03	0.45 ± 0.07	0.26 ± 0.04	0.18 ± 0.05	0.17 ± 0.04
	Chryseolinea (483)	0.05 ± 0.02	0.00 ± 0.00	0.03 ± 0.01	0.15 ± 0.07	0.07 ± 0.02	0.09 ± 0.01	0.07 ± 0.03
	Chryseolinea (231)	0.21 ± 0.02	0.03 ± 0.01	0.13 ± 0.01	0.32 ± 0.06	0.19 ± 0.02	0.20 ± 0.04	0.13 ± 0.04
	Chryseolinea (1195)	0.02 ± 0.00	0.00 ± 0.00	0.02 ± 0.00	0.04 ± 0.01	0.04 ± 0.01	0.02 ± 0.01	0.02 ± 0.01
	Uncultured *Rhodothermaceae* (363)	0.02 ± 0.01	0.00 ± 0.00	0.00 ± 0.00	0.02 ± 0.00	0.10 ± 0.01	0.01 ± 0.01	0.04 ± 0.03
	Ferruginibacter (499)	0.09 ± 0.01	0.00 ± 0.00	0.01 ± 0.01	0.04 ± 0.00	0.04 ± 0.01	0.09 ± 0.00	0.15 ± 0.05
	Ferruginibacter (378)	0.02 ± 0.01	0.02. ± 0.01	0.05 ± 0.02	0.11 ± 0.04	0.06 ± 0.01	0.09 ± 0.01	0.16 ± 0.04
	Flavobacterium (2394)	0.01 ± 0.00	0.00 ± 0.00	0.00 ± 0.00	0.00 ± 0.00	0.00 ± 0.00	0.12 ± 0.09	0.00 ± 0.00
	Flavobacterium (376)	0.02 ± 0.01	0.00 ± 0.00	0.00 ± 0.00	0.20 ± 0.07	0.08 ± 0.02	0.04 ± 0.01	0.00 ± 0.00
	Uncultured *Cytophagaceae* (451)	0.11 ± 0.02	0.00 ± 0.00	0.02 ± 0.01	0.10 ± 0.04	0.09 ± 0.04	0.03 ± 0.01	0.10 ± 0.04
	Ferruginibacter (256)	0.00 ± 0.00	0.02 ± 0.01	0.06 ± 0.01	0.19 ± 0.08	0.14 ± 0.01	0.13 ± 0.02	0.21 ± 0.07
	Uncultured *Saprospiraceae* (380)	0.00 ± 0.00	0.02 ± 0.01	0.04 ± 0.02	0.05 ± 0.01	0.03 ± 0.01	0.13 ± 0.05	0.16 ± 0.04
	Uncultured *Saprospiraceae* (383)	0.00 ± 0.00	0.01 ± 0.00	0.02 ± 0.00	0.04 ± 0.01	0.18 ± 0.08	0.09 ± 0.02	0.13 ± 0.01

	Uncultured *Saprospiraceae* (472)	0.04 ± 0.01	0.00 ± 0.00	0.01 ± 0.01	0.02 ± 0.01	0.02 ± 0.01	0.14 ± 0.07	0.29 ± 0.14
	Uncultured Sphingobacteriales (711)	0.02 ± 0.01	0.00 ± 0.00	0.02 ± 0.01	0.08 ± 0.02	0.02 ± 0.00	0.12 ± 0.01	0.04 ± 0.02
	Uncultured env.OPS_17 (726)	0.01 ± 0.00	0.00 ± 0.00	0.00 ± 0.00	0.02 ± 0.01	0.00 ± 0.00	0.20 ± 0.08	0.00 ± 0.00
	Uncultured env.OPS_17 (876)	0.01 ± 0.00	0.00 ± 0.00	0.02 ± 0.01	0.05 ± 0.02	0.01 ± 0.00	0.11 ± 0.03	0.01 ± 0.01
	Uncultured env.OPS_17 (548)	0.01 ± 0.01	0.00 ± 0.00	0.00 ± 0.00	0.00 ± 0.00	0.05 ± 0.00	0.01 ± 0.01	0.09 ± 0.09
	Uncultured KD3-93 (46)	0.33 ± 0.06	0.00 ± 0.00	0.00 ± 0.00	0.17 ± 0.00	0.02 ± 0.01	0.61 ± 0.26	0.09 ± 0.06
	NS11-12_marine_group (616)	0.01 ± 0.01	0.00 ± 0.00	0.00 ± 0.00	0.09 ± 0.02	0.03 ± 0.01	0.14 ± 0.03	0.03 ± 0.02
	PHOS-HE51 (1186)	0.00 ± 0.00	0.00 ± 0.00	0.00 ± 0.00	0.02 ± 0.01	0.01 ± 0.00	0.07 ± 0.03	0.04 ± 0.01
	Uncultured Sphingobacteriales (409)	0.23 ± 0.09	0.00 ± 0.00	0.00 ± 0.00	0.00 ± 0.00	0.04 ± 0.01	0.00 ± 0.00	0.09 ± 0.03
	Terrimonas (139)	0.02 ± 0.00	0.02 ± 0.01	0.03 ± 0.01	0.22 ± 0.05	0.14 ± 0.03	0.21 ± 0.05	0.72 ± 0.21
	Terrimonas (149)	0.04 ± 0.00	0.03 ± 0.01	0.07 ± 0.01	0.36 ± 0.27	0.06 ± 0.01	0.15 ± 0.07	0.12 ± 0.05
	Terrimonas (253)	0.03 ± 0.01	0.03 ± 0.00	0.12 ± 0.02	0.17 ± 0.02	0.10 ± 0.01	0.15 ± 0.02	0.11 ± 0.03
Cand_div_BRC1	Uncultured Candidate_div_BRC1 (542)	0.00 ± 0.00	0.00 ± 0.00	0.02 ± 0.01	0.02 ± 0.01	0.01± 0.00	0.00 ± 0.00	0.01 ± 0.01
	Uncultured Candidate_div_BRC1 (1346)	0.01 ± 0.00	0.00 ± 0.00	0.00 ± 0.00	0.00 ± 0.00	0.00 ± 0.00	0.00 ± 0.00	0.00 ± 0.00
Gemmatimonadetes	Uncultured *Gemmatimonadaceae* (124)	0.00 ± 0.00	0.01 ± 0.01	0.08 ± 0.03	0.13 ± 0.01	0.10 ± 0.01	0.17 ± 0.01	0.13 ± 0.01
	Uncultured *Gemmatimonadaceae* (32)	0.51 ± 0.07	0.14 ± 0.04	0.41 ± 0.04	0.62 ± 0.08	1.06 ± 0.07	0.41 ± 0.04	0.48 ± 0.08
	Uncultured *Gemmatimonadaceae* (52)	0.31 ± 0.05	0.09 ± 0.02	0.28 ± 0.05	0.30 ± 0.08	0.42 ± 0.05	0.21 ± 0.05	0.26 ± 0.05
	Uncultured *Gemmatimonadaceae* (251)	0.00 ± 0.00	0.00 ± 0.00	0.06 ± 0.00	0.06 ± 0.03	0.09 ± 0.00	0.05 ± 0.02	0.08 ± 0.01
	Uncultured *Gemmatimonadaceae* (269)	0.09 ± 0.05	0.01 ± 0.00	0.04 ± 0.01	0.10 ± 0.01	0.07 ± 0.01	0.06 ± 0.02	0.06 ± 0.01
	Uncultured *Gemmatimonadaceae* (299)	0.01 ± 0.01	0.03 ± 0.00	0.02 ± 0.00	0.06 ± 0.00	0.17 ± 0.01	0.07 ± 0.03	0.13 ± 0.07
	Uncultured *Gemmatimonadaceae* (388)	0.06 ± 0.02	0.05 ± 0.01	0.07 ± 0.02	0.08 ± 0.01	0.08 ± 0.01	0.04 ± 0.01	0.03 ± 0.01
Chlorobi	Uncultured BSV26 (580)	0.02 ± 0.01	0.00 ± 0.00	0.02 ± 0.01	0.06 ± 0.01	0.02 ± 0.01	0.04 ± 0.00	0.01 ± 0.01
	Uncultured BSV26 (93)	0.20 ± 0.01	0.11 ± 0.04	0.33 ± 0.13	0.63 ± 0.04	0.24 ± 0.01	0.45 ± 0.09	0.18 ± 0.02
	Uncultured BSV26 (413)	0.05 ± 0.01	0.01 ± 0.00	0.06 ± 0.01	0.10 ± 0.03	0.05 ± 0.01	0.13 ± 0.03	0.05 ± 0.01

	Uncultured OPB56 (740)	0.00 ± 0.00	0.00 ± 0.00	0.01 ± 0.00	0.02 ± 0.00	0.00 ± 0.00	0.04 ± 0.03	0.01 ± 0.00
	Uncultured SJA-28 (284)	0.07 ± 0.00	0.07 ± 0.00	0.21 ± 0.02	0.26 ± 0.04	0.12 ± 0.01	0.10 ± 0.01	0.11 ± 0.01
Chloroflexi	Uncultured *Caldilineaceae* (110)	0.14 ± 0.02	0.09 ± 0.00	0.76 ± 0.07	0.27 ± 0.05	0.38 ± 0.09	0.21 ± 0.06	0.38 ± 0.11
	Uncultured KD4-96 (27)	0.08 ± 0.02	0.31 ± 0.10	0.56 ± 0.02	0.56 ± 0.04	0.97 ± 0.08	0.47 ± 0.06	0.93 ± 0.09
	Uncultured *Anaerolineaceae* (233)	0.10 ± 0.02	0.02 ± 0.01	0.15 ± 0.01	0.09 ± 0.01	0.19 ± 0.01	0.11 ± 0.03	0.12 ± 0.03
	Uncultured *Anaerolineaceae* (611)	0.04 ± 0.01	0.00 ± 0.00	0.00 ± 0.00	0.04 ± 0.01	0.05 ± 0.02	0.03 ± 0.02	0.13 ± 0.04
	Uncultured *Anaerolineaceae* (628)	0.04 ± 0.01	0.00 ± 0.00	0.03 ± 0.01	0.04 ± 0.02	0.07 ± 0.01	0.03 ± 0.02	0.04 ± 0.01
	Unclassified Gitt-GS-136 (127)	0.14 ± 0.02	0.11 ± 0.10	0.13 ± 0.02	0.17 ± 0.03	0.42 ± 0.02	0.10 ± 0.01	0.30 ± 0.05
	Uncultured KD4-96 (96)	0.12 ± 0.03	0.20 ± 0.03	0.16 ± 0.03	0.20 ± 0.03	0.66 ± 0.05	0.14 ± 0.04	0.20 ± 0.04
	Unclassified JG30-KF-CM45 (145)	0.05 ± 0.00	0.05 ± 0.01	0.13 ± 0.02	0.13 ± 0.01	0.24 ± 0.03	0.18 ± 0.03	0.43 ± 0.08
	Uncultured KD4-96 (8)	0.12 ± 0.05	1.59 ± 0.47	1.33 ± 0.04	1.18 ± 0.02	1.06 ± 0.03	1.08 ± 0.11	1.18 ± 0.06
	Uncultured KD4-96 (16)	0.38 ± 0.04	0.83 ± 0.20	0.43 ± 0.07	0.74 ± 0.05	1.09 ± 0.05	0.48 ± 0.05	1.04 ± 0.10
Latescibacteria	Uncultured Latescibacteria (95)	0.21 ± 0.01	0.04 ± 0.01	0.07 ± 0.01	0.18 ± 0.02	0.15 ± 0.02	0.18 ± 0.01	0.08 ± 0.03
	Uncultured Latescibacteria (154)	0.14 ± 0.01	0.00 ± 0.00	0.06 ± 0.03	0.12 ± 0.01	0.02 ± 0.01	0.08 ± 0.03	0.06 ± 0.02
	Uncultured Latescibacteria (308)	0.01 ± 0.00	0.01 ± 0.01	0.01 ± 0.01	0.01 ± 0.01	0.10 ± 0.02	0.01 ± 0.01	0.15 ± 0.05
	Uncultured Latescibacteria (326)	0.08 ± 0.00	0.02 ± 0.01	0.04 ± 0.02	0.08 ± 0.02	0.04 ± 0.01	0.07 ± 0.02	0.04 ± 0.01
	Uncultured Latescibacteria (342)	0.06 ± 0.00	0.02 ± 0.01	0.07 ± 0.01	0.05 ± 0.02	0.05 ± 0.01	0.03 ± 0.01	0.04 ± 0.00
	Uncultured Latescibacteria (453)	0.00 ± 0.00	0.02 ± 0.01	0.06 ± 0.01	0.03 ± 0.01	0.06 ± 0.00	0.03 ± 0.01	0.04 ± 0.00
	Uncultured Latescibacteria (503)	0.03 ± 0.00	0.00 ± 0.00	0.02 ± 0.01	0.06 ± 0.01	0.02 ± 0.00	0.03 ± 0.01	0.01 ± 0.01
	Uncultured Latescibacteria (534)	0.02 ± 0.00	0.02 ± 0.00	0.04 ± 0.01	0.06 ± 0.01	0.08 ± 0.02	0.03 ± 0.00	0.03 ± 0.01
	Uncultured Latescibacteria (706)	0.01 ± 0.01	0.01 ± 0.00	0.12 ± 0.03	0.02 ± 0.00	0.03 ± 0.01	0.01 ± 0.01	0.01± 0.00
	Uncultured Latescibacteria (732)	0.01 ± 0.00	0.00 ± 0.00	0.02 ± 0.00	0.02 ± 0.01	0 .00 ± 0.00	0.01 ± 0.00	0.01 ± 0.00
	Uncultured Latescibacteria (864)	0.01 ± 0.01	0.00 ± 0.00	0.01 ± 0.01	0.01 ± 0.00	0.02 ± 0.01	0.01 ± 0.01	0.02 ± 0.01
	Uncultured Latescibacteria (422)	0.07 ± 0.01	0.05 ± 0.02	0.25 ± 0.04	0.10 ± 0.01	0.06 ± 0.01	0.05 ± 0.01	0.04 ± 0.00

	Uncultured Latescibacteria (1054)	0.00 ± 0.00	0.00 ± 0.00	0.01 ± 0.00	0.01 ± 0.01	0.02 ± 0.01	0.01 ± 0.01	0.01 ± 0.01
Nitrospirae	*Nitrospira* (7)	0.85 ± 0.14	0.1 ± 0.01	0.22 ± 0.03	0.36 ± 0.03	0.29 ± 0.04	0.62 ± 0.06	0.29 ± 0.08
	Nitrospira (23)	1.12 ± 0.20	0.09 ± 0.02	0.36 ± 0.08	0.48 ± 0.14	0.15 ± 0.02	0.41 ± 0.08	0.20 ± 0.03
	Nitrospira (26)	0.70 ± 0.09	0.04 ± 0.01	0.32 ± 0.06	0.28 ± 0.03	0.13 ± 0.01	0.19 ± 0.09	0.06 ± 0.03
Alphaproteobacteria	*Sphingobium* (17)	0.61 ± 0.07	0.00 ± 0.00	0.00 ± 0.00	0.30 ± 0.28	0.08 ± 0.02	0.04 ± 0.02	0.04 ± 0.02
	Hyphomicrobium (60)	0.35 ± 0.07	0.09 ± 0.02	0.30 ± 0.04	0.23 ± 0.02	0.29 ± 0.03	0.29 ± 0.00	0.23 ± 0.03
	Hyphomicrobium (347)	0.06 ± 0.02	0.02 ± 0.00	0.04 ± 0.04	0.00 ± 0.00	0.08 ± 0.01	0.02 ± 0.02	0.07 ± 0.01
	Hirschia (62)	0.07 ± 0.02	0.05 ± 0.01	0.27 ± 0.04	0.25 ± 0.04	0.30 ± 0.03	0.36 ± 0.04	0.45 ± 0.20
	Novosphingobium (64)	0.00 ± 0.00	0.02 ± 0.00	0.01 ± 0.01	0.05 ± 0.01	2.04 ± 0.78	0.51 ± 0.48	0.03 ± 0.02
	Pedomicrobium (84)	0.07 ± 0.01	0.08 ± 0.01	0.22 ± 0.02	0.15 ± 0.02	0.28 ± 0.03	0.26 ± 0.01	0.38 ± 0.06
	Uncultured Rhizobiales (335)	0.16 ± 0.03	0.03 ± 0.01	0.10 ± 0.04	0.04 ± 0.01	0.13 ± 0.03	0.17 ± 0.04	0.15 ± 0.06
	Defluviimonas (202)	0.00 ± 0.00	0.03 ± 0.00	0.10 ± 0.01	0.23 ± 0.03	0.15 ± 0.01	0.20 ± 0.03	0.13 ± 0.01
	Hyphomicrobium (129)	0.09 ± 0.02	0.05 ± 0.00	0.16 ± 0.03	0.12 ± 0.01	0.26 ± 0.02	0.13 ± 0.00	0.19 ± 0.03
	Uncultured AKYH478 (645)	0.01 ± 0.00	0.00 ± 0.00	0.02 ± 0.01	0.03 ± 0.02	0.04 ± 0.00	0.06 ± 0.01	0.19 ± 0.06
	Uncultured F0723 (1059)	0.04 ± 0.01	0.01 ± 0.00	0.01 ± 0.00	0.00 ± 0.00	0.01 ± 0.00	0.03 ± 0.02	0.09 ± 0.03
	Uncultured MNG7 (35)	0.00 ± 0.00	0.20 ± 0.04	0.66 ± 0.11	0.75 ± 0.03	0.43 ± 0.02	0.89 ± 0.08	0.83 ± 0.13
	Uncultured *Phyllobacteriaceae* (87)	0.14 ± 0.02	0.04 ± 0.01	0.04 ± 0.01	0.04 ± 0.01	0.05 ± 0.01	0.16 ± 0.05	0.39 ± 0.12
	Uncultured *Sphingomonadaceae* (1)	0.00 ± 0.00	0.13 ± 0.02	0.15 ± 0.03	1.30 ± 0.16	0.28 ± 0.02	6.16 ± 4.93	4.14 ± 1.16
	Uncultured *Sphingomonadaceae* (119)	0.00 ± 0.00	0.01 ± 0.01	0.05 ± 0.01	0.27 ± 0.02	0.18 ± 0.04	0.29 ± 0.01	0.26 ± 0.09
	Sphingopyxis (553)	0.02 ± 0.00	0.00 ± 0.00	0.09 ± 0.02	0.08 ± 0.01	0.01 ± 0.01	0.08 ± 0.00	0.01 ± 0.00
	Woodsholea (181)	0.07 ± 0.01	0.00 ± 0.00	0.11 ± 0.01	0.22 ± 0.03	0.25 ± 0.07	0.23 ± 0.04	0.09 ± 0.02
	Woodsholea (183)	0.01 ± 0.00	0.02 ± 0.00	0.16 ± 0.01	0.21 ± 0.03	0.15 ± 0.03	0.15 ± 0.02	0.10 ± 0.01
	Woodsholea (615)	0.04 ± 0.01	0.01 ± 0.00	0.05 ± 0.01	0.04 ± 0.01	0.04 ± 0.01	0.10 ± 0.02	0.02 ± 0.00
	Uncultured *Rhizobiaceae* (100)	0.46 ± 0.10	0.02 ± 0.01	0.07 ± 0.02	0.19 ± 0.01	0.09 ± 0.02	0.21 ± 0.03	0.32 ± 0.19

	Uncultured AKYH478 (645)	0.01 ± 0.00	0.00 ± 0.00	0.02 ± 0.01	0.03 ± 0.02	0.04 ± 0.00	0.06 ± 0.01	0.19 ± 0.06
	Defluviimonas (202)	0.00 ± 0.00	0.03 ± 0.00	0.10 ± 0.01	0.23 ± 0.03	0.15 ± 0.01	0.20 ± 0.03	0.13 ± 0.01
Deltaproteobacteria	*Bdellovibrio* (63)	0.05 ± 0.00	0.00 ± 0.00	0.00 ± 0.00	0.46 ± 0.11	0.00 ± 0.01	0.08 ± 0.00	0.11 ± 0.07
	Haliangium (479)	0.01 ± 0.00	0.00 ± 0.00	0.02 ± 0.00	0.04 ± 0.02	0.04 ± 0.01	0.01 ± 0.00	0.02 ± 0.00
	Haliangium (248)	0.00 ± 0.00	0.00 ± 0.00	0.00 ± 0.00	0.01 ± 0.00	0.08 ± 0.04	0.00 ± 0.00	0.12 ± 0.06
	Haliangium (457)	0.10 ± 0.02	0.00 ± 0.00	0.01 ± 0.00	0.02 ± 0.00	0.06 ± 0.01	0.01 ± 0.01	0.03 ± 0.02
	Haliangium (739)	0.00 ± 0.00	0.00 ± 0.00	0.01 ± 0.00	0.02 ± 0.01	0.02 ± 0.01	0.02 ± 0.01	0.02 ± 0.01
	OM27-Clade (166)	0.01 ± 0.00	0.00 ± 0.00	0.00 ± 0.00	0.01 ± 0.00	0.00 ± 0.00	0.11 ± 0.05	0.08 ± 0.07
	Anaeromyxobacter (220)	0.15 ± 0.02	0.01 ± 0.00	0.02 ± 0.01	0.05 ± 0.02	0.05 ± 0.00	0.06 ± 0.01	0.06 ± 0.02
	Anaeromyxobacter (82)	0.24 ± 0.02	0.16 ± 0.01	0.16 ± 0.01	0.17 ± 0.03	0.16 ± 0.01	0.14 ± 0.03	0.06 ± 0.02
	Sandaracinus (260)	0.05 ± 0.01	0.00 ± 0.00	0.01 ± 0.00	0.05 ± 0.03	0.02 ± 0.01	0.02 ± 0.01	0.03 ± 0.02
	Sandaracinus (382)	0.00 ± 0.00	0.00 ± 0.00	0.01 ± 0.01	0.04 ± 0.02	0.01 ± 0.00	0.02 ± 0.01	0.01 ± 0.00
	Sorangium (709)	0.01 ± 0.01	0.00 ± 0.00	0.03 ± 0.00	0.01 ± 0.01	0.01 ± 0.00	0.02 ± 0.00	0.00 ± 0.00
	Sorangium (1348)	0.00 ± 0.00	0.00 ± 0.00	0.01 ± 0.00	0.00 ± 0.00	0.01 ± 0.00	0.00 ± 0.00	0.00 ± 0.00
	Sorangium (352)	0.00 ± 0.00	0.01 ± 0.00	0.01 ± 0.00	0.00 ± 0.00	0.05 ± 0.02	0.00 ± 0.00	0.04 ± 0.02
	Uncultured *Cystobacteraceae* (203)	0.02 ± 0.00	0.00 ± 0.00	0.05 ± 0.01	0.02 ± 0.00	0.02 ± 0.00	0.05 ± 0.01	0.06 ± 0.02
	Uncultured *Sandaracinaceae* (33)	0.16 ± 0.01	0.00 ± 0.00	0.11 ± 0.02	0.24 ± 0.05	0.21 ± 0.05	0.12 ± 0.04	0.04 ± 0.01
	Uncultured *Sandaracinaceae* (404)	0.04 ± 0.01	0.00 ± 0.00	0.01 ± 0.00	0.05 ± 0.01	0.02 ± 0.00	0.01 ± 0.01	0.00 ± 0.00
	Uncultured *Sandaracinaceae* (686)	0.07 ± 0.01	0.00 ± 0.00	0.00 ± 0.00	0.04 ± 0.01	0.00 ± 0.00	0.02 ± 0.01	0.00 ± 0.00
	Uncultured *Sandaracinaceae* (240)	0.1 ± 0.03	0.00 ± 0.00	0.02 ± 0.01	0.06 ± 0.02	0.05 ± 0.02	0.09 ± 0.00	0.04 ± 0.01
	Uncultured *Sandaracinaceae* (764)	0.01 ± 0.01	0.00 ± 0.00	0.00 ± 0.00	0.00 ± 0.00	0.01 ± 0.01	0.00 ± 0.00	0.00 ± 0.00
	Uncultured Sh765B-TzT-29 (414)	0.05 ± 0.02	0.02 ± 0.01	0.10 ± 0.01	0.10 ± 0.01	0.06 ± 0.01	0.07 ± 0.02	0.09 ± 0.01
	Uncultured δ-bacteria (105)	0.18 ± 0.02	0.00 ± 0.00	0.00 ± 0.00	0.00 ± 0.00	0.00 ± 0.00	0.00 ± 0.00	0.07 ± 0.05
	Uncultured δ-bacteria (54)	0.37 ± 0.04	0.00 ± 0.00	0.00 ± 0.00	0.00 ± 0.00	0.04 ± 0.03	0.00 ± 0.00	0.02 ± 0.02

	Phaselicystis (163)	0.02 ± 0.01	0.00 ± 0.00	0.00 ± 0.00	0.00 ± 0.00	0.00 ± 0.00	0.00 ± 0.00	0.12 ± 0.01
Gammaproteobacteria (Betaproteobacteriales)	*Hydrogenophaga* (9)	1.15 ± 0.17	0.02 ± 0.00	0.11 ± 0.02	0.21 ± 0.03	0.35 ± 0.08	0.32 ± 0.13	1.29 ± 0.13
	Leeia (513)	0.05 ± 0.02	0.00 ± 0.00	0.01 ± 0.01	0.10 ± 0.03	0.03 ± 0.01	0.01 ± 0.00	0.00 ± 0.00
	Piscinibacter (14)	0.16 ± 0.01	0.03 ± 0.00	0.13 ± 0.01	0.27 ± 0.04	0.17 ± 0.01	0.29 ± 0.02	0.76 ± 0.12
	Rhizobacter (123)	0.33 ± 0.03	0.00 ± 0.00	0.07 ± 0.01	0.04 ± 0.01	0.01 ± 0.00	0.20 ± 0.09	0.05 ± 0.02
	Uncultured *Comamonadaceae* (4)	0.00 ± 0.00	0.10 ± 0.02	0.28 ± 0.05	0.40 ± 0.03	0.28 ± 0.01	0.58 ± 0.14	1.29 ± 0.26
	Uncultured *Comamonadaceae* (36)	0.24 ± 0.04	0.02 ± 0.00	0.07 ± 0.01	0.19 ± 0.05	0.16 ± 0.03	0.26 ± 0.06	0.70 ± 0.16
	Uncultured *Comamonadaceae* (113)	0.05 ± 0.01	0.01 ± 0.00	0.00 ± 0.00	0.11 ± 0.06	0.08 ± 0.05	0.45 ± 0.25	0.28 ± 0.14
	Uncultured *Comamonadaceae* (152)	0.02 ± 0.00	0.02 ± 0.01	0.01 ± 0.00	0.05 ± 0.01	0.03 ± 0.00	0.07 ± 0.01	0.34 ± 0.24
	Uncultured *Comamonadaceae* (1113)	0.04 ± 0.01	0.00 ± 0.00	0.00 ± 0.00	0.01 ± 0.01	0.04 ± 0.02	0.04 ± 0.02	0.02 ± 0.01
	Uncultured *Comamonadaceae* (12)	0.46 ± 0.12	0.17 ± 0.03	0.35 ± 0.04	0.32 ± 0.02	0.17 ± 0.01	0.40 ± 0.07	0.52 ± 0.08
	Azohydromonas (43)	0.53 ± 0.10	0.02 ± 0.01	0.06 ± 0.02	0.05 ± 0.02	0.05 ± 0.01	0.11 ± 0.02	0.16 ± 0.04
	Uncultured *Gallionellaceae* (320)	0.10 ± 0.00	0.02 ± 0.01	0.06 ± 0.03	0.16 ± 0.02	0.05 ± 0.01	0.13 ± 0.02	0.06 ± 0.03
	Uncultured *Nitrosomonadaceae* (20)	0.11 ± 0.00	0.35 ± 0.04	0.65 ± 0.08	0.81 ± 0.03	0.70 ± 0.12	1.18 ± 0.04	1.33 ± 0.30
	Uncultured *Nitrosomonadaceae* (350)	0.05 ± 0.01	0.01 ± 0.01	0.05 ± 0.02	0.13 ± 0.02	0.03 ± 0.02	0.19 ± 0.02	0.34 ± 0.19
	Uncultured *Nitrosomonadaceae* (92)	0.07 ± 0.01	0.21 ± 0.04	0.24 ± 0.04	0.39 ± 0.05	0.27 ± 0.05	0.30 ± 0.02	0.23 ± 0.03
	Uncultured *Oxalobacteraceae* (215)	0.14 ± 0.04	0.01 ± 0.00	0.00 ± 0.00	0.15 ± 0.04	0.04 ± 0.01	0.11 ± 0.01	0.03 ± 0.01
	Vogesella (44)	0.03 ± 0.01	0.00 ± 0.00	0.00 ± 0.00	0.00 ± 0.00	0.00 ± 0.00	0.00 ± 0.00	0.00 ± 0.00
	Dechloromonas (138)	0.11 ± 0.01	0.01 ± 0.00	0.01 ± 0.01	0.01 ± 0.01	0.00 ± 0.00	0.03 ± 0.01	0.01 ± 0.00
	Thauera (40)	0.13 ± 0.01	0.00 ± 0.00	0.00 ± 0.00	0.01 ± 0.01	0.01 ± 0.00	0.02 ± 0.01	0.01 ± 0.00
	Uncultured SC-I-84 (89)	0.20 ± 0.04	0.21 ± 0.03	0.24 ± 0.02	0.29 ± 0.04	0.26 ± 0.03	0.44 ± 0.11	0.35 ± 0.12
	Uncultured SC-I-84 (354)	0.07 ± 0.00	0.05 ± 0.00	0.07 ± 0.01	0.08 ± 0.03	0.07 ± 0.01	0.16 ± 0.05	0.15 ± 0.03
	Uncultured β-proteobacteria (66)	0.14 ± 0.00	0.15 ± 0.01	0.10 ± 0.02	0.16 ± 0.05	0.19 ± 0.05	0.34 ± 0.03	0.83 ± 0.25
	Zoogloea (266)	0.00 ± 0.00	0.00 ± 0.00	0.00 ± 0.00	0.00 ± 0.00	0.00 ± 0.00	0.00 ± 0.00	0.00 ± 0.00

	Acidiferrobacter (67)	0.17 ± 0.03	0.16 ± 0.01	0.13 ± 0.05	0.48 ± 0.04	0.20 ± 0.05	0.53 ± 0.04	0.19 ± 0.06
	Arenimonas (520)	0.02 ± 0.01	0.00 ± 0.00	0.01 ± 0.00	0.06 ± 0.01	0.10 ± 0.01	0.03 ± 0.00	0.02 ± 0.01
	Arenimonas (357)	0.00 ± 0.00	0.00 ± 0.00	0.02 ± 0.01	0.25 ± 0.05	0.06 ± 0.01	0.25 ± 0.06	0.03 ± 0.01
	Pseudomonas (24)	0.09 ± 0.02	0.00 ± 0.00	0.08 ± 0.01	0.50 ± 0.12	0.15 ± 0.03	0.41 ± 0.06	0.08 ± 0.03
	Uncultured *Pseudomonadaceae* (3)	0.00 ± 0.00	0.00 ± 0.00	0.00 ± 0.00	0.00 ± 0.00	0.00 ± 0.00	0.00 ± 0.00	0.00 ± 0.00
	Thermomonas (58)	0.07 ± 0.01	0.05 ± 0.01	0.06 ± 0.01	0.43 ± 0.14	0.32 ± 0.08	0.59 ± 0.07	0.37 ± 0.12
	Uncultured *Xanthomonadaceae* (70)	0.05 ± 0.01	0.11 ± 0.04	0.41 ± 0.01	0.75 ± 0.07	0.21 ± 0.01	0.31 ± 0.02	0.47 ± 0.15
	Uncultured γ-proteobacteria (452)	0.00 ± 0.00	0.00 ± 0.00	0.01 ± 0.00	0.08 ± 0.02	0.02 ± 0.00	0.05 ± 0.02	0.01 ± 0.00
	Uncultured γ-proteobacteria (541)	0.02 ± 0.00	0.00 ± 0.00	0.00 ± 0.00	0.05 ± 0.01	0.02 ± 0.01	0.00 ± 0.00	0.00 ± 0.00
	Uncultured γ-proteobacteria (505)	0.04 ± 0.01	0.00 ± 0.00	0.03 ± 0.02	0.09 ± 0.03	0.01 ± 0.01	0.11 ± 0.01	0.02 ± 0.01
	Uncultured γ-proteobacteria (715)	0.01 ± 0.01	0.00 ± 0.00	0.02 ± 0.01	0.06 ± 0.02	0.02 ± 0.01	0.03 ± 0.01	0.00 ± 0.00
	Uncultured γ-proteobacteria (1758)	0.00 ± 0.00	0.00 ± 0.00	0.00 ± 0.00	0.08 ± 0.06	0.04 ± 0.01	0.00 ± 0.00	0.00 ± 0.00
	Acidiferrobacter (196)	0.02 ± 0.01	0.06 ± 0.01	0.08 ± 0.02	0.21 ± 0.03	0.21 ± 0.01	0.24 ± 0.02	0.15 ± 0.02
Saccharibacteria	Uncultured Saccharibacteria (980)	0.01 ± 0.00	0.00 ± 0.00	0.00 ± 0.00	0.13 ± 0.05	0.05 ± 0.01	0.05 ± 0.03	0.02 ± 0.01
Verrucomicrobia	*Prosthecobacter* (162)	0.20 ± 0.04	0.00 ± 0.00	0.02 ± 0.01	0.04 ± 0.02	0.05 ± 0.01	0.40 ± 0.02	0.10 ± 0.03
	Verrucomicrobium (903)	0.01 ± 0.01	0.00 ± 0.00	0.00 ± 0.00	0.01 ± 0.01	0.01 ± 0.00	0.11 ± 0.03	0.20 ± 0.05
	Uncultured DEV007 (1517)	0.00 ± 0.00	0.00 ± 0.00	0.00 ± 0.00	0.00 ± 0.00	0.00 ± 0.00	0.00 ± 0.00	0.10 ± 0.03
Unclassified	Uncultured bacterium (315)	0.01 ± 0.00	0.00 ± 0.00	0.01 ± 0.01	0.11 ± 0.04	0.42 ± 0.09	0.00 ± 0.00	0.00 ± 0.00
	Uncultured bacterium (255)	0.19 ± 0.02	0.00 ± 0.00	0.00 ± 0.00	0.01 ± 0.00	0.05 ± 0.02	0.03 ± 0.01	0.06 ± 0.05
		**RNA**						
Phylum/Subphylum-level	Genus-level (OTU No.)	0_0‘	0_3‘	0_5‘	40_3‘	40_5‘	400_3‘	400_5‘
Acidobacteria	*Bryobacter* (144)	0.06 ± 0.02	0.04 ± 0.01	0.15 ± 0.02	0.21 ± 0.01	0.21 ± 0.06	0.26 ± 0.02	0.12 ± 0.01
	Bryobacter (61)	0.27 ± 0.05	0.14 ± 0.06	0.51 ± 0.08	0.62 ± 0.03	0.33 ± 0.03	0.63 ± 0.01	0.52 ± 0.10

	Bryobacter (115)	0.11 ± 0.03	0.06 ± 0.03	0.55 ± 0.11	0.16 ± 0.02	0.29 ± 0.09	0.15 ± 0.06	0.28 ± 0.04
	Bryobacter (562)	0.01 ± 0.00	0.01 ± 0.00	0.03 ± 0.01	0.04 ± 0.01	0.04 ± 0.00	0.08 ± 0.02	0.13 ± 0.03
	Uncultured subgroup_3 (901)	0.02 ± 0.01	0.00 ± 0.00	0.03 ± 0.00	0.04 ± 0.01	0.02 ± 0.01	0.07 ± 0.02	0.04 ± 0.01
	Uncultured subgroup_3 (55)	0.47 ± 0.04	0.20 ± 0.13	0.81 ± 0.01	0.39 ± 0.03	0.33 ± 0.08	0.66 ± 0.05	0.65 ± 0.14
	Uncultured subgroup_3 (516)	0.04 ± 0.00	0.01 ± 0.01	0.06 ± 0.01	0.09 ± 0.01	0.04 ± 0.01	0.09 ± 0.00	0.08 ± 0.01
	Blastocatella (126)	0.03 ± 0.01	0.03 ± 0.01	0.05 ± 0.01	0.03 ± 0.01	0.05 ± 0.02	0.05 ± 0.02	0.12 ± 0.02
	Uncultured subgroup_6 (85)	0.00 ± 0.01	0.02 ± 0.00	0.02 ± 0.01	0.07 ± 0.02	0.06 ± 0.01	0.06 ± 0.03	0.47 ± 0.16
	Uncultured subgroup_6 (10)	0.14 ± 0.02	0.18 ± 0.04	1.21 ± 0.27	0.79 ± 0.07	1.34 ± 0.18	0.94 ± 0.24	1.56 ± 0.44
	Uncultured subgroup_6 (212)	0.03 ± 0.01	0.01 ± 0.00	0.15 ± 0.02	0.21 ± 0.05	0.23 ± 0.01	0.25 ± 0.10	0.14 ± 0.05
	Uncultured subgroup_6 (304)	0.01 ± 0.00	0.02 ± 0.00	0.06 ± 0.00	0.15 ± 0.03	0.19 ± 0.05	0.09 ± 0.01	0.12 ± 0.04
	Uncultured subgroup_6 (401)	0.01 ± 0.00	0.01 ± 0.00	0.04 ± 0.02	0.06 ± 0.01	0.06 ± 0.01	0.07 ± 0.03	0.06 ± 0.01
	Uncultured subgroup_6 (581)	0.03 ± 0.02	0.00 ± 0.00	0.17 ± 0.05	0.08 ± 0.02	0.04 ± 0.01	0.05 ± 0.00	0.04 ± 0.01
	Uncultured subgroup_6 (412)	0.01 ± 0.00	0.01 ± 0.00	0.06 ± 0.01	0.04 ± 0.01	0.08 ± 0.00	0.06 ± 0.00	0.03 ± 0.01
	Uncultured subgroup_6 (147)	0.01 ± 0.00	0.06 ± 0.02	0.01 ± 0.00	0.06 ± 0.02	0.15 ± 0.04	0.09 ± 0.01	0.08 ± 0.02
	Uncultured subgroup_6 (85)	0.00 ± 0.00	0.02 ± 0.00	0.02 ± 0.01	0.07 ± 0.02	0.06 ± 0.01	0.06 ± 0.03	0.47 ± 0.16
	Uncultured subgroup_17 (39)	0.07 ± 0.00	0.07 ± 0.03	0.41 ± 0.03	0.39 ± 0.05	0.34 ± 0.01	0.41 ± 0.09	0.30 ± 0.11
	Uncultured subgroup_22 (329)	0.03 ± 0.00	0.01 ± 0.00	0.10 ± 0.03	0.12 ± 0.03	0.21 ± 0.01	0.06 ± 0.01	0.06 ± 0.02
	Uncultured subgroup_22 (340)	0.03 ± 0.00	0.02 ± 0.02	0.24 ± 0.07	0.13 ± 0.03	0.10 ± 0.00	0.11 ± 0.02	0.05 ± 0.02
	Uncultured subgroup_22 (57)	0.10 ± 0.05	0.07 ± 0.03	0.47 ± 0.09	0.71 ± 0.25	0.43 ± 0.05	0.89 ± 0.18	0.54 ± 0.02
Actinobacteria	Uncultured Sva0996_marine_group (83)	0.01 ± 0.00	0.01 ± 0.00	0.05 ± 0.02	0.05 ± 0.01	0.12 ± 0.03	0.07 ± 0.02	0.09 ± 0.08
	Aeromicrobium (332)	0.00 ± 0.00	0.00 ± 0.00	0.00 ± 0.00	0.00 ± 0.00	0.01 ± 0.00	0.00 ± 0.00	0.04 ± 0.02
	Marmoricola (69)	0.04 ± 0.01	0.04 ± 0.01	0.03 ± 0.00	0.13 ± 0.02	0.13 ± 0.00	0.04 ± 0.00	0.12 ± 0.06
	Nocardioides (372)	0.01 ± 0.00	0.00 ± 0.01	0.00 ± 0.00	0.00 ± 0.00	0.00 ± 0.00	0.01 ± 0.00	0.01 ± 0.01
	Nocardioides (210)	0.00 ± 0.00	0.01 ± 0.00	0.01 ± 0.00	0.05 ± 0.01	0.09 ± 0.02	0.01 ± 0.01	0.08 ± 0.04

	Nocardioides (102)	0.03 ± 0.00	0.02 ± 0.01	0.03 ± 0.01	0.06 ± 0.02	0.02 ± 0.01	0.03 ± 0.01	0.04 ± 0.01
	Uncultured MB-A2-108 (94)	0.02 ± 0.00	0.01 ± 0.00	0.10 ± 0.01	0.17 ± 0.02	0.10 ± 0.01	0.14 ± 0.04	0.07 ± 0.02
	Iamia (188)	0.01 ± 0.00	0.01 ± 0.01	0.02 ± 0.01	0.12 ± 0.01	0.06 ± 0.01	0.14 ± 0.05	0.06 ± 0.01
	Iamia (362)	0.01 ± 0.00	0.00 ± 0.00	0.01 ± 0.00	0.02 ± 0.00	0.04 ± 0.01	0.00 ± 0.00	0.03 ± 0.01
	Uncultured Acidimicrobiales (1265)	0.00 ± 0.00	0.00 ± 0.00	0.00 ± 0.00	0.03 ± 0.01	0.00 ± 0.00	0.02 ± 0.02	0.00 ± 0.00
	Uncultured 480-2(627)	0.01 ± 0.01	0.00 ± 0.00	0.00 ± 0.00	0.02 ± 0.01	0.03 ± 0.01	0.01 ± 0.00	0.00 ± 0.00
	Solirubrobacter (29)	0.02 ± 0.01	0.01 ± 0.00	0.02 ± 0.01	0.07 ± 0.02	0.08 ± 0.00	0.03 ± 0.00	0.04 ± 0.01
	Sva0996_marine_group(441)	0.01 ± 0.00	0.01 ± 0.01	0.06 ± 0.02	0.12 ± 0.02	0.09 ± 0.02	0.15 ± 0.08	0.11 ± 0.03
	Flavobacterium (633)	0.00 ± 0.00	0.00 ± 0.00	0.00 ± 0.00	0.02 ± 0.00	0.00 ± 0.00	0.04 ± 0.02	0.00 ± 0.00
	Flavobacterium (2394)	0.00 ± 0.00	0.00 ± 0.00	0.00 ± 0.00	0.00 ± 0.00	0.00 ± 0.00	0.02 ± 0.02	0.00 ± 0.00
	Uncultured NS9_marine_group (438)	0.01 ± 0.01	0.01 ± 0.01	0.05 ± 0.02	0.03 ± 0.02	0.01 ± 0.00	0.13 ± 0.10	0.10 ± 0.06
	Uncultured NS9_marine_group (277)	0.03 ± 0.01	0.02 ± 0.01	0.04 ± 0.01	0.04 ± 0.01	0.03 ± 0.01	0.21 ± 0.17	0.09 ± 0.03
	Rhodococcus (459)	0.00 ± 0.00	0.00 ± 0.00	0.07 ± 0.01	0.05 ± 0.04	0.08 ± 0.03	0.08 ± 0.02	0.10 ± 0.04
	Fodinicola (15)	0.13 ± 0.04	0.13 ± 0.06	0.28 ± 0.02	0.59 ± 0.02	0.38 ± 0.06	0.55 ± 0.01	0.19 ± 0.05
	Nocardioides (405)	0.01 ± 0.00	0.01 ± 0.00	0.02 ± 0.01	0.01 ± 0.00	0.01 ± 0.00	0.01 ± 0.01	0.02 ± 0.01
	Uncultured Acidimicrobiales (612)	0.01 ± 0.00	0.00 ± 0.00	0.07 ± 0.02	0.07 ± 0.01	0.02 ± 0.01	0.03 ± 0.01	0.03 ± 0.01
	Uncultured Actinobacteria (359)	0.01 ± 0.01	0.01 ± 0.00	0.02 ± 0.01	0.07 ± 0.01	0.01 ± 0.01	0.05 ± 0.01	0.02 ± 0.02
	Uncultured MB-A2-108 (137)	0.03 ± 0.01	0.01 ± 0.01	0.12 ± 0.01	0.23 ± 0.03	0.13 ± 0.03	0.22 ± 0.03	0.06 ± 0.01
	CL500-29_marine_group (28)	0.02 ± 0.01	0.05 ± 0.01	0.04 ± 0.01	0.10 ± 0.01	0.12 ± 0.00	0.13 ± 0.01	1.34 ± 0.14
	Uncultured Acidimicrobiales (569)	0.01 ± 0.00	0.01 ± 0.00	0.00 ± 0.00	0.07 ± 0.01	0.03 ± 0.01	0.04 ± 0.02	0.00 ± 0.00
	Uncultured *Intrasporangiaceae* (19)	0.07 ± 0.02	0.17 ± 0.03	0.06 ± 0.01	0.19 ± 0.02	0.15 ± 0.01	0.16 ± 0.01	0.10 ± 0.02
	Gaiella (45)	0.05 ± 0.01	0.11 ± 0.06	0.13 ± 0.01	0.24 ± 0.02	0.43 ± 0.02	0.16 ± 0.01	0.19 ± 0.04
Armatimonadetes	Uncultured Armatimonadetes (371)	0.00 ± 0.00	0.00 ± 0.00	0.00 ± 0.00	0.00 ± 0.00	0.03 ± 0.02	0.00 ± 0.00	0.18 ± 0.01
Bacteroidetes	*Chryseolinea* (165)	0.01 ± 0.00	0.00 ± 0.00	0.02 ± 0.01	0.05 ± 0.01	0.02 ± 0.01	0.03 ± 0.01	0.03 ± 0.00

	Chryseolinea (483)	0.01 ± 0.00	0.01 ± 0.01	0.00 ± 0.00	0.02 ± 0.00	0.02 ± 0.01	0.02 ± 0.00	0.01 ± 0.01
	Chryseolinea (231)	0.04 ± 0.01	0.02 ± 0.01	0.01 ± 0.00	0.06 ± 0.01	0.03 ± 0.01	0.05 ± 0.02	0.03 ± 0.00
	Chryseolinea (1195)	0.02 ± 0.01	0.00 ± 0.00	0.01 ± 0.00	0.01 ± 0.01	0.00 ± 0.00	0.01 ± 0.00	0.00 ± 0.00
	Uncultured *Rhodothermaceae* (363)	0.00 ± 0.00	0.00 ± 0.00	0.00 ± 0.00	0.01 ± 0.00	0.12 ± 0.02	0.02 ± 0.01	0.09 ± 0.07
	Ferruginibacter (499)	0.00 ± 0.00	0.01 ± 0.01	0.00 ± 0.00	0.02 ± 0.01	0.01 ± 0.01	0.04 ± 0.02	0.06 ± 0.02
	Ferruginibacter (378)	0.01 ± 0.01	0.00 ± 0.00	0.00 ± 0.00	0.02 ± 0.00	0.01 ± 0.00	0.02 ± 0.01	0.04 ± 0.02
	Flavobacterium (2394)	0.00 ± 0.00	0.00 ± 0.00	0.00 ± 0.00	0.00 ± 0.00	0.00 ± 0.00	0.02 ± 0.02	0.00 ± 0.00
	Flavobacterium (376)	0.00 ± 0.00	0.00 ± 0.00	0.00 ± 0.00	0.04 ± 0.01	0.01 ± 0.01	0.01 ± 0.00	0.00 ± 0.00
	Uncultured *Cytophagaceae* (451)	0.00 ± 0.00	0.01 ± 0.00	0.01 ± 0.00	0.01 ± 0.01	0.00 ± 0.00	0.00 ± 0.00	0.01 ± 0.00
	Ferruginibacter (256)	0.01 ± 0.00	0.02 ± 0.01	0.01 ± 0.00	0.03 ± 0.01	0.02 ± 0.00	0.02 ± 0.00	0.06 ± 0.02
	Uncultured *Saprospiraceae* (380)	0.00 ± 0.00	0.01 ± 0.01	0.03 ± 0.02	0.03 ± 0.00	0.04 ± 0.02	0.09 ± 0.04	0.12 ± 0.04
	Uncultured *Saprospiraceae* (383)	0.00 ± 0.00	0.00 ± 0.00	0.00 ± 0.00	0.01 ± 0.00	0.02 ± 0.01	0.01 ± 0.01	0.01 ± 0.00
	Uncultured *Saprospiraceae* (472)	0.00 ± 0.00	0.00 ± 0.00	0.00 ± 0.00	0.01 ± 0.00	0.01 ± 0.00	0.03 ± 0.02	0.10 ± 0.05
	Uncultured Sphingobacteriales (711)	0.00 ± 0.00	0.00 ± 0.00	0.00 ± 0.00	0.00 ± 0.00	0.00 ± 0.00	0.01 ± 0.01	0.01 ± 0.01
	Uncultured env.OPS_17 (726)	0.00 ± 0.00	0.00 ± 0.00	0.00 ± 0.00	0.01 ± 0.00	0.00 ± 0.00	0.05 ± 0.01	0.00 ± 0.00
	Uncultured env.OPS_17 (876)	0.00 ± 0.00	0.00 ± 0.00	0.00 ± 0.00	0.02 ± 0.00	0.00 ± 0.00	0.02 ± 0.00	0.00 ± 0.00
	Uncultured env.OPS_17 (548)	0.03 ± 0.01	0.00 ± 0.00	0.02 ± 0.00	0.01 ± 0.00	0.20 ± 0.00	0.00 ± 0.00	0.55 ± 0.51
	Uncultured KD3-93 (46)	0.00 ± 0.00	0.00 ± 0.00	0.01 ± 0.01	0.69 ± 0.15	0.05 ± 0.00	2.92 ± 1.93	0.32 ± 0.16
	NS11-12_marine_group (616)	0.00 ± 0.00	0.00 ± 0.00	0.00 ± 0.00	0.01 ± 0.00	0.01 ± 0.00	0.02 ± 0.01	0.01 ± 0.01
	PHOS-HE51 (1186)	0.00 ± 0.00	0.00 ± 0.00	0.00 ± 0.00	0.00 ± 0.00	0.00 ± 0.00	0.03 ± 0.02	0.05 ± 0.01
	Uncultured Sphingobacteriales (409)	0.00 ± 0.00	0.00 ± 0.00	0.00 ± 0.00	0.00 ± 0.00	0.12 ± 0.07	0.02 ± 0.01	0.41 ± 0.16
	Terrimonas (139)	0.01 ± 0.00	0.01 ± 0.00	0.00 ± 0.00	0.02 ± 0.01	0.01 ± 0.00	0.02 ± 0.01	0.06 ± 0.03
	Terrimonas (149)	0.01 ± 0.00	0.01 ± 0.00	0.01 ± 0.01	0.02 ± 0.01	0.00 ± 0.00	0.03 ± 0.01	0.02 ± 0.01
	Terrimonas (253)	0.02 ± 0.01	0.02 ± 0.00	0.03 ± 0.01	0.03 ± 0.01	0.02 ± 0.01	0.03 ± 0.01	0.01 ± 0.01

Cand_div_BRC1	Uncultured Candidate_div_BRC1 (542)	0.01 ± 0.00	0.00 ± 0.00	0.13 ± 0.08	0.22 ± 0.10	0.03 ± 0.01	0.05 ± 0.03	0.01 ± 0.00
	Uncultured Candidate_div_BRC1 (1346)	0.00 ± 0.00	0.00 ± 0.00	0.04 ± 0.02	0.09 ± 0.04	0.00 ± 0.00	0.02 ± 0.01	0.03 ± 0.02
Gemmatimonadetes	Uncultured *Gemmatimonadaceae* (124)	0.01 ± 0.01	0.01 ± 0.01	0.14 ± 0.03	0.32 ± 0.06	0.23 ± 0.05	0.29 ± 0.03	0.26 ± 0.05
	Uncultured *Gemmatimonadaceae* (32)	0.03 ± 0.01	0.06 ± 0.05	0.21 ± 0.03	0.77 ± 0.17	0.67 ± 0.12	0.64 ± 0.07	0.14 ± 0.03
	Uncultured *Gemmatimonadaceae* (52)	0.02 ± 0.00	0.06 ± 0.04	0.28 ± 0.03	0.57 ± 0.10	0.56 ± 0.05	0.46 ± 0.09	0.37 ± 0.04
	Uncultured *Gemmatimonadaceae* (251)	0.01 ± 0.01	0.01 ± 0.00	0.07 ± 0.02	0.22 ± 0.05	0.18 ± 0.04	0.28 ± 0.07	0.08 ± 0.01
	Uncultured *Gemmatimonadaceae* (269)	0.02 ± 0.00	0.00 ± 0.00	0.09 ± 0.02	0.15 ± 0.03	0.11 ± 0.01	0.17 ± 0.03	0.12 ± 0.03
	Uncultured *Gemmatimonadaceae* (299)	0.00 ± 0.00	0.01 ± 0.00	0.01 ± 0.00	0.05 ± 0.01	0.15 ± 0.02	0.09 ± 0.07	0.10 ± 0.08
	Uncultured *Gemmatimonadaceae* (388)	0.02 ± 0.00	0.01 ± 0.01	0.06 ± 0.03	0.09 ± 0.00	0.11 ± 0.00	0.09 ± 0.02	0.05 ± 0.04
Chlorobi	Uncultured BSV26 (580)	0.05 ± 0.02	0.01 ± 0.01	0.06 ± 0.01	0.06 ± 0.02	0.04 ± 0.02	0.05 ± 0.00	0.02 ± 0.01
	Uncultured BSV26 (93)	0.02 ± 0.01	0.01 ± 0.00	0.05 ± 0.01	0.06 ± 0.01	0.04 ± 0.02	0.04 ± 0.00	0.03 ± 0.01
	Uncultured BSV26 (413)	0.03 ± 0.02	0.02 ± 0.01	0.02 ± 0.01	0.06 ± 0.01	0.05 ± 0.00	0.08 ± 0.00	0.06 ± 0.01
	Uncultured OPB56 (740)	0.02 ± 0.01	0.00 ± 0.00	0.05 ± 0.02	0.12 ± 0.02	0.01 ± 0.00	0.13 ± 0.11	0.03 ± 0.01
	Uncultured SJA-28 (284)	0.02 ± 0.00	0.02 ± 0.01	0.03 ± 0.01	0.01 ± 0.00	0.01 ± 0.00	0.00 ± 0.00	0.02 ± 0.01
Chloroflexi	Uncultured *Caldilineaceae* (110)	0.01 ± 0.00	0.01 ± 0.00	0.22 ± 0.05	0.12 ± 0.01	0.03 ± 0.02	0.05 ± 0.02	0.10 ± 0.02
	Uncultured KD4-96 (27)	0.05 ± 0.01	0.07 ± 0.02	0.16 ± 0.04	0.39 ± 0.05	0.26 ± 0.02	0.30 ± 0.04	0.39 ± 0.00
	Uncultured *Anaerolineaceae* (233)	0.02 ± 0.01	0.01 ± 0.01	0.08 ± 0.03	0.06 ± 0.02	0.21 ± 0.00	0.05 ± 0.00	0.14 ± 0.03
	Uncultured *Anaerolineaceae* (611)	0.00 ± 0.00	0.00 ± 0.00	0.01 ± 0.00	0.01 ± 0.00	0.06 ± 0.02	0.00 ± 0.00	0.12 ± 0.03
	Uncultured *Anaerolineaceae* (628)	0.00 ± 0.00	0.01 ± 0.00	0.02 ± 0.01	0.02 ± 0.00	0.11 ± 0.00	0.00 ± 0.00	0.06 ± 0.03
	Unclassified Gitt-GS-136 (127)	0.01 ± 0.00	0.02 ± 0.01	0.05 ± 0.01	0.08 ± 0.02	0.14 ± 0.05	0.04 ± 0.01	0.10 ± 0.04
	Uncultured KD4-96 (96)	0.04 ± 0.00	0.02 ± 0.01	0.06 ± 0.02	0.11 ± 0.01	0.14 ± 0.02	0.12 ± 0.02	0.08 ± 0.01
	Unclassified JG30-KF-CM45 (145)	0.01 ± 0.01	0.02 ± 0.01	0.08 ± 0.02	0.10 ± 0.01	0.16 ± 0.01	0.24 ± 0.16	0.27 ± 0.11
	Uncultured KD4-96 (8)	0.26 ± 0.05	0.32 ± 0.12	0.48 ± 0.12	1.35 ± 0.08	0.45 ± 0.00	1.13 ± 0.08	0.47 ± 0.06
	Uncultured KD4-96 (16)	0.11 ± 0.03	0.16 ± 0.02	0.16 ± 0.07	0.62 ± 0.05	0.48 ± 0.08	0.49 ± 0.08	0.54 ± 0.02

Latescibacteria	Uncultured Latescibacteria (95)	0.06 ± 0.03	0.01 ± 0.00	0.51 ± 0.15	0.57 ± 0.11	0.27 ± 0.03	0.49 ± 0.06	0.25 ± 0.11
	Uncultured Latescibacteria (154)	0.01 ± 0.00	0.00 ± 0.00	0.35 ± 0.12	0.40 ± 0.05	0.23 ± 0.08	0.35 ± 0.03	0.31 ± 0.15
	Uncultured Latescibacteria (308)	0.01 ± 0.00	0.01 ± 0.01	0.01 ± 0.01	0.04 ± 0.01	0.12 ± 0.05	0.03 ± 0.01	0.20 ± 0.07
	Uncultured Latescibacteria (326)	0.02 ± 0.01	0.00 ± 0.00	0.11 ± 0.03	0.18 ± 0.02	0.14 ± 0.06	0.13 ± 0.00	0.08 ± 0.03
	Uncultured Latescibacteria (342)	0.04 ± 0.02	0.02 ± 0.01	0.08 ± 0.01	0.13 ± 0.05	0.11 ± 0.01	0.04 ± 0.00	0.07 ± 0.02
	Uncultured Latescibacteria (453)	0.00 ± 0.00	0.01 ± 0.00	0.13 ± 0.01	0.09 ± 0.02	0.09 ± 0.01	0.08 ± 0.01	0.07 ± 0.02
	Uncultured Latescibacteria (503)	0.02 ± 0.01	0.00 ± 0.00	0.11 ± 0.03	0.13 ± 0.03	0.08 ± 0.00	0.10 ± 0.02	0.06 ± 0.03
	Uncultured Latescibacteria (534)	0.01 ± 0.00	0.00 ± 0.00	0.04 ± 0.01	0.05 ± 0.01	0.16 ± 0.05	0.01 ± 0.01	0.03 ± 0.02
	Uncultured Latescibacteria (706)	0.02 ± 0.01	0.00 ± 0.00	0.19 ± 0.01	0.07 ± 0.01	0.02 ± 0.00	0.04 ± 0.01	0.02 ± 0.00
	Uncultured Latescibacteria (732)	0.00 ± 0.00	0.00 ± 0.00	0.09 ± 0.02	0.10 ± 0.03	0.06 ± 0.03	0.04 ± 0.00	0.02 ± 0.01
	Uncultured Latescibacteria (864)	0.02 ± 0.00	0.00 ± 0.00	0.02 ± 0.00	0.05 ± 0.01	0.09 ± 0.02	0.06 ± 0.01	0.05 ± 0.01
	Uncultured Latescibacteria (422)	0.01 ± 0.00	0.00 ± 0.00	0.28 ± 0.06	0.10 ± 0.02	0.06 ± 0.00	0.07 ± 0.01	0.04 ± 0.02
	Uncultured Latescibacteria (1054)	0.00 ± 0.00	0.00 ± 0.00	0.04 ± 0.01	0.05 ± 0.01	0.04 ± 0.00	0.02 ± 0.01	0.02 ± 0.01
Nitrospirae	*Nitrospira* (7)	0.79 ± 0.18	0.47 ± 0.09	1.02 ± 0.09	0.89 ± 0.12	1.57 ± 0.20	2.32 ± 0.61	1.44 ± 0.22
	Nitrospira (23)	0.49 ± 0.04	0.39 ± 0.08	1.86 ± 0.58	0.77 ± 0.12	1.51 ± 0.17	0.74 ± 0.07	0.80 ± 0.20
	Nitrospira (26)	0.52 ± 0.02	0.61 ± 0.03	1.36 ± 0.13	1.16 ± 0.16	0.73 ± 0.12	0.91 ± 0.38	0.42 ± 0.21
Alphaproteobacteria	*Sphingobium* (17)	0.00 ± 0.00	0.00 ± 0.00	0.00 ± 0.00	0.12 ± 0.11	0.05 ± 0.00	0.03 ± 0.02	0.02 ± 0.01
	Hyphomicrobium (60)	0.22 ± 0.04	0.23 ± 0.02	0.44 ± 0.08	0.27 ± 0.06	0.38 ± 0.06	0.22 ± 0.02	0.59 ± 0.10
	Hyphomicrobium (347)	0.00 ± 0.00	0.00 ± 0.00	0.16 ± 0.04	0.08 ± 0.01	0.13 ± 0.06	0.09 ± 0.01	0.19 ± 0.06
	Hirschia (62)	0.09 ± 0.01	0.16 ± 0.06	0.34 ± 0.11	0.15 ± 0.00	0.55 ± 0.01	0.14 ± 0.01	1.18 ± 0.76
	Novosphingobium (64)	0.01 ± 0.01	0.01 ± 0.00	0.01 ± 0.00	0.01 ± 0.00	0.62 ± 0.10	0.14 ± 0.01	0.03 ± 0.02
	Pedomicrobium (84)	0.09 ± 0.02	0.07 ± 0.02	0.15 ± 0.03	0.21 ± 0.03	0.08 ± 0.00	0.30 ± 0.00	0.48 ± 0.22
	Uncultured Rhizobiales (335)	0.01 ± 0.00	0.02 ± 0.01	0.03 ± 0.01	0.01 ± 0.00	0.03 ± 0.02	0.01 ± 0.01	0.04 ± 0.04
	Defluviimonas (202)	0.06 ± 0.01	0.05 ± 0.01	0.09 ± 0.02	0.07 ± 0.01	0.04 ± 0.01	0.03 ± 0.01	0.11 ± 0.04

	Hyphomicrobium (129)	0.11 ± 0.01	0.06 ± 0.01	0.15 ± 0.02	0.22 ± 0.02	0.14 ± 0.01	0.23 ± 0.01	0.22 ± 0.14
	Uncultured AKYH478 (645)	0.01 ± 0.00	0.01 ± 0.01	0.00 ± 0.00	0.01 ± 0.01	0.01 ± 0.01	0.03 ± 0.00	0.12 ± 0.05
	Uncultured F0723 (1059)	0.03 ± 0.00	0.02 ± 0.00	0.00 ± 0.00	0.00 ± 0.00	0.00 ± 0.00	0.03 ± 0.01	0.09 ± 0.04
	Uncultured MNG7 (35)	0.15 ± 0.01	0.17 ± 0.02	0.12 ± 0.01	0.11 ± 0.01	0.09 ± 0.02	0.13 ± 0.01	0.24 ± 0.07
	Uncultured *Phyllobacteriaceae* (87)	0.09 ± 0.01	0.20 ± 0.04	0.05 ± 0.00	0.02 ± 0.01	0.05 ± 0.03	0.09 ± 0.06	0.48 ± 0.13
	Uncultured *Sphingomonadaceae* (1)	0.09 ± 0.02	0.10 ± 0.01	0.06 ± 0.02	3.26 ± 0.56	0.28 ± 0.01	7.71 ± 4.09	4.41 ± 0.57
	Uncultured *Sphingomonadaceae* (119)	0.00 ± 0.00	0.01 ± 0.00	0.01 ± 0.00	0.03 ± 0.01	0.08 ± 0.06	0.02 ± 0.01	0.08 ± 0.01
	Sphingopyxis (553)	0.02 ± 0.00	0.04 ± 0.01	0.02 ± 0.00	0.02 ± 0.01	0.00 ± 0.00	0.01 ± 0.01	0.01 ± 0.01
	Woodsholea (181)	0.03 ± 0.00	0.02 ± 0.01	0.09 ± 0.02	0.07 ± 0.00	0.22 ± 0.05	0.06 ± 0.00	0.06 ± 0.03
	Woodsholea (183)	0.02 ± 0.01	0.07 ± 0.01	0.10 ± 0.02	0.08 ± 0.02	0.11 ± 0.03	0.05 ± 0.02	0.12 ± 0.05
	Woodsholea (615)	0.02 ± 0.01	0.02 ± 0.01	0.05 ± 0.02	0.02 ± 0.00	0.02 ± 0.01	0.02 ± 0.00	0.02 ± 0.02
	Uncultured *Rhizobiaceae* (100)	0.04 ± 0.03	0.07 ± 0.01	0.03 ± 0.01	0.08 ± 0.02	0.10 ± 0.05	0.02 ± 0.00	0.26 ± 0.20
	Uncultured AKYH478 (645)	0.01 ± 0.00	0.01 ± 0.01	0.00 ± 0.00	0.01 ± 0.01	0.01 ± 0.01	0.03 ± 0.00	0.12 ± 0.05
	Defluviimonas (202)	0.06 ± 0.01	0.05 ± 0.01	0.09 ± 0.02	0.07 ± 0.01	0.04 ± 0.01	0.03 ± 0.01	0.11 ± 0.04
Deltaproteobacteria	*Bdellovibrio* (63)	0.01 ± 0.00	0.00 ± 0.00	0.00 ± 0.00	0.98 ± 0.42	0.01 ± 0.00	0.16 ± 0.04	0.26 ± 0.19
	Haliangium (479)	0.01 ± 0.00	0.00 ± 0.00	0.07 ± 0.01	0.07 ± 0.01	0.07 ± 0.02	0.02 ± 0.01	0.02 ± 0.00
	Haliangium (248)	0.00 ± 0.00	0.00 ± 0.00	0.00 ± 0.00	0.05 ± 0.01	0.27 ± 0.12	0.02 ± 0.01	0.39 ± 0.19
	Haliangium (457)	0.00 ± 0.00	0.00 ± 0.00	0.04 ± 0.02	0.08 ± 0.02	0.13 ± 0.00	0.05 ± 0.03	0.03 ± 0.02
	Haliangium (739)	0.01 ± 0.00	0.00 ± 0.00	0.04 ± 0.02	0.07 ± 0.01	0.04 ± 0.02	0.05 ± 0.02	0.03 ± 0.01
	OM27-Clade (166)	0.02 ± 0.00	0.02 ± 0.01	0.02 ± 0.00	0.11 ± 0.02	0.01 ± 0.01	0.61 ± 0.46	0.47 ± 0.37
	Anaeromyxobacter (220)	0.05 ± 0.01	0.05 ± 0.01	0.12 ± 0.03	0.20 ± 0.04	0.24 ± 0.07	0.18 ± 0.01	0.18 ± 0.01
	Anaeromyxobacter (82)	0.16 ± 0.00	0.15 ± 0.05	0.29 ± 0.08	0.53 ± 0.09	0.45 ± 0.06	0.59 ± 0.07	0.17 ± 0.06
	Sandaracinus (260)	0.01 ± 0.00	0.00 ± 0.00	0.07 ± 0.01	0.25 ± 0.08	0.16 ± 0.01	0.07 ± 0.06	0.18 ± 0.13
	Sandaracinus (382)	0.01 ± 0.00	0.00 ± 0.00	0.09 ± 0.01	0.26 ± 0.06	0.01 ± 0.00	0.06 ± 0.04	0.02 ± 0.02

	Sorangium (709)	0.00 ± 0.00	0.00 ± 0.00	0.17 ± 0.02	0.10 ± 0.02	0.10 ± 0.04	0.04 ± 0.01	0.01 ± 0.01
	Sorangium (1348)	0.00 ± 0.00	0.00 ± 0.00	0.06 ± 0.02	0.06 ± 0.01	0.01 ± 0.00	0.00 ± 0.00	0.00 ± 0.00
	Sorangium (352)	0.00 ± 0.00	0.00 ± 0.00	0.03 ± 0.01	0.03 ± 0.00	0.36 ± 0.10	0.00 ± 0.00	0.26 ± 0.12
	Uncultured *Cystobacteraceae* (203)	0.05 ± 0.00	0.03 ± 0.00	0.21 ± 0.03	0.12 ± 0.04	0.05 ± 0.01	0.21 ± 0.04	0.27 ± 0.09
	Uncultured *Sandaracinaceae* (33)	0.01 ± 0.01	0.00 ± 0.00	0.73 ± 0.08	1.20 ± 0.17	1.50 ± 0.17	0.44 ± 0.14	0.21 ± 0.04
	Uncultured *Sandaracinaceae* (404)	0.00 ± 0.00	0.00 ± 0.00	0.07 ± 0.01	0.21 ± 0.05	0.09 ± 0.01	0.02 ± 0.00	0.01 ± 0.00
	Uncultured *Sandaracinaceae* (686)	0.00 ± 0.00	0.00 ± 0.00	0.00 ± 0.00	0.11 ± 0.03	0.00 ± 0.01	0.04 ± 0.02	0.00 ± 0.00
	Uncultured *Sandaracinaceae* (240)	0.00 ± 0.00	0.00 ± 0.00	0.07 ± 0.02	0.18 ± 0.04	0.28 ± 0.01	0.14 ± 0.00	0.11 ± 0.04
	Uncultured *Sandaracinaceae* (764)	0.00 ± 0.00	0.00 ± 0.00	0.01 ± 0.00	0.03 ± 0.01	0.13 ± 0.03	0.00 ± 0.00	0.00 ± 0.00
	Uncultured Sh765B-TzT-29 (414)	0.00 ± 0.00	0.00 ± 0.00	0.03 ± 0.00	0.02 ± 0.01	0.05 ± 0.01	0.02 ± 0.00	0.05 ± 0.01
	Uncultured δ-bacteria (105)	0.00 ± 0.00	0.00 ± 0.00	0.00 ± 0.00	0.00 ± 0.01	0.00 ± 0.00	0.00 ± 0.00	0.21 ± 0.15
	Uncultured δ-bacteria (54)	0.00 ± 0.00	0.00 ± 0.00	0.00 ± 0.00	0.01 ± 0.00	0.28 ± 0.21	0.00 ± 0.00	0.18 ± 0.11
	Phaselicystis (163)	0.00 ± 0.00	0.00 ± 0.00	0.00 ± 0.00	0.00 ± 0.00	0.00 ± 0.00	0.00 ± 0.00	0.30 ± 0.28
Gammaproteobacteria (Betaproteobacterales)	*Hydrogenophaga* (9)	0.01 ± 0.00	0.01 ± 0.00	0.10 ± 0.01	0.24 ± 0.02	0.61 ± 0.01	0.24 ± 0.14	1.11 ± 0.14
	Leeia (513)	0.05 ± 0.01	0.02 ± 0.01	0.03 ± 0.01	0.15 ± 0.06	0.05 ± 0.03	0.02 ± 0.00	0.01 ± 0.01
	Piscinibacter (14)	0.17 ± 0.02	0.07 ± 0.03	0.65 ± 0.23	0.48 ± 0.06	1.27 ± 0.19	0.46 ± 0.01	1.76 ± 0.44
	Rhizobacter (123)	0.01 ± 0.00	0.01 ± 0.00	0.20 ± 0.07	0.12 ± 0.03	0.09 ± 0.02	0.26 ± 0.11	0.17 ± 0.06
	Uncultured *Comamonadaceae* (4)	0.28 ± 0.06	0.23 ± 0.03	0.90 ± 0.18	0.71 ± 0.11	1.35 ± 0.21	1.03 ± 0.01	2.70 ± 0.48
	Uncultured *Comamonadaceae* (36)	0.03 ± 0.02	0.02 ± 0.00	0.29 ± 0.09	0.25 ± 0.05	0.64 ± 0.10	0.49 ± 0.13	1.98 ± 0.36
	Uncultured *Comamonadaceae* (113)	0.01 ± 0.00	0.01 ± 0.00	0.00 ± 0.00	0.09 ± 0.05	0.09 ± 0.05	0.38 ± 0.31	0.30 ± 0.15
	Uncultured *Comamonadaceae* (152)	0.06 ± 0.02	0.02 ± 0.01	0.14 ± 0.04	0.13 ± 0.02	0.26 ± 0.01	0.18 ± 0.01	0.65 ± 0.23
	Uncultured *Comamonadaceae* (1113)	0.01 ± 0.00	0.00 ± 0.00	0.00 ± 0.00	0.01 ± 0.00	0.03 ± 0.03	0.13 ± 0.01	0.01 ± 0.01
	Uncultured *Comamonadaceae* (12)	0.6 ± 0.07	0.51 ± 0.07	0.91 ± 0.21	0.51 ± 0.03	1.01 ± 0.14	0.65 ± 0.11	1.58 ± 0.34
	Azohydromonas (43)	0.07 ± 0.02	0.03 ± 0.01	0.16 ± 0.04	0.11 ± 0.01	0.11 ± 0.03	0.14 ± 0.00	0.47 ± 0.09

	Uncultured *Gallionellaceae* (320)	0.06 ± 0.03	0.10 ± 0.01	0.07 ± 0.02	0.08 ± 0.01	0.03 ± 0.00	0.05 ± 0.02	0.02 ± 0.01
	Uncultured *Nitrosomonadaceae* (20)	0.07 ± 0.03	0.05 ± 0.02	0.06 ± 0.01	0.09 ± 0.02	0.13 ± 0.04	0.14 ± 0.01	0.21 ± 0.06
	Uncultured *Nitrosomonadaceae* (350)	0.01 ± 0.01	0.03 ± 0.02	0.01 ± 0.00	0.01 ± 0.01	0.01 ± 0.01	0.00 ± 0.00	0.04 ± 0.03
	Uncultured *Nitrosomonadaceae* (92)	0.04 ± 0.03	0.04 ± 0.01	0.03 ± 0.02	0.03 ± 0.01	0.04 ± 0.00	0.03 ± 0.01	0.05 ± 0.02
	Uncultured *Oxalobacteraceae* (215)	0.00 ± 0.00	0.00 ± 0.00	0.00 ± 0.00	0.07 ± 0.05	0.03 ± 0.01	0.03 ± 0.01	0.01 ± 0.00
	Vogesella (44)	0.00 ± 0.00	0.00 ± 0.00	0.00 ± 0.00	0.00 ± 0.00	0.00 ± 0.00	0.00 ± 0.00	0.00 ± 0.00
	Dechloromonas (138)	0.05 ± 0.00	0.04 ± 0.01	0.04 ± 0.00	0.03 ± 0.00	0.01 ± 0.00	0.04 ± 0.00	0.02 ± 0.01
	Thauera (40)	0.01 ± 0.00	0.01 ± 0.01	0.02 ± 0.00	0.02 ± 0.01	0.00 ± 0.00	0.04 ± 0.00	0.02 ± 0.01
	Uncultured SC-I-84 (89)	0.05 ± 0.01	0.02 ± 0.02	0.06 ± 0.01	0.09 ± 0.01	0.04 ± 0.01	0.19 ± 0.05	0.13 ± 0.03
	Uncultured SC-I-84 (354)	0.01 ± 0.01	0.00 ± 0.00	0.01 ± 0.00	0.02 ± 0.01	0.01 ± 0.00	0.03 ± 0.02	0.03 ± 0.01
	Uncultured β-proteobacteria (66)	0.13 ± 0.07	0.07 ± 0.00	0.05 ± 0.00	0.19 ± 0.05	0.15 ± 0.00	0.30 ± 0.00	0.57 ± 0.10
	Zoogloea (266)	0.00 ± 0.00	0.00 ± 0.00	0.00 ± 0.00	0.00 ± 0.00	0.00 ± 0.00	0.00 ± 0.00	0.00 ± 0.00
	Acidiferrobacter (67)	0.36 ± 0.12	0.41 ± 0.10	0.18 ± 0.02	0.31 ± 0.03	0.23 ± 0.05	0.26 ± 0.04	0.10 ± 0.02
	Arenimonas (520)	0.00 ± 0.00	0.00 ± 0.00	0.01 ± 0.00	0.08 ± 0.01	0.07 ± 0.02	0.08 ± 0.00	0.01 ± 0.00
	Arenimonas (357)	0.00 ± 0.00	0.00 ± 0.00	0.01 ± 0.00	0.07 ± 0.01	0.00 ± 0.00	0.09 ± 0.03	0.02 ± 0.01
	Pseudomonas (24)	0.00 ± 0.00	0.02 ± 0.01	0.08 ± 0.01	0.33 ± 0.12	0.19 ± 0.02	0.21 ± 0.04	0.06 ± 0.01
	Unclutured *Pseudomonadaceae* (3)	0.00 ± 0.00	0.02 ± 0.01	0.00 ± 0.00	0.00 ± 0.00	0.00 ± 0.00	0.00 ± 0.00	0.00 ± 0.00
	Thermomonas (58)	0.02 ± 0.01	0.06 ± 0.02	0.05 ± 0.01	0.12 ± 0.03	0.19 ± 0.09	0.15 ± 0.03	0.09 ± 0.03
	Uncultured *Xanthomonadaceae* (70)	0.02 ± 0.00	0.07 ± 0.03	0.07 ± 0.02	0.14 ± 0.01	0.05 ± 0.02	0.03 ± 0.00	0.11 ± 0.03
	Uncultured γ-proteobacteria (452)	0.00 ± 0.00	0.00 ± 0.00	0.07 ± 0.02	0.14 ± 0.03	0.07 ± 0.03	0.06 ± 0.02	0.02 ± 0.01
	Uncultured γ-proteobacteria (541)	0.00 ± 0.00	0.00 ± 0.00	0.00 ± 0.00	0.13 ± 0.01	0.10 ± 0.01	0.00 ± 0.00	0.00 ± 0.00
	Uncultured γ-proteobacteria (505)	0.02 ± 0.02	0.04 ± 0.01	0.05 ± 0.02	0.05 ± 0.02	0.04 ± 0.01	0.07 ± 0.02	0.05 ± 0.01
	Uncultured γ-proteobacteria (715)	0.00 ± 0.00	0.00 ± 0.00	0.01 ± 0.01	0.05 ± 0.01	0.01 ± 0.00	0.07 ± 0.05	0.00 ± 0.00
	Uncultured γ-proteobacteria (1758)	0.00 ± 0.00	0.00 ± 0.00	0.00 ± 0.00	0.01 ± 0.01	0.00 ± 0.00	0.00 ± 0.00	0.00 ± 0.00

	Acidiferrobacter (196)	0.13 ± 0.06	0.13 ± 0.05	0.05 ± 0.02	0.07 ± 0.01	0.05 ± 0.00	0.06 ± 0.02	0.03 ± 0.00
Saccharibacteria	Uncultured Saccharibacteria (980)	0.00 ± 0.00	0.00 ± 0.00	0.00 ± 0.00	0.01 ± 0.00	0.01 ± 0.00	0.00 ± 0.00	0.00 ± 0.00
Verrucomicrobia	*Prosthecobacter* (162)	0.03 ± 0.01	0.02 ± 0.01	0.03 ± 0.00	0.10 ± 0.02	0.07 ± 0.04	0.57 ± 0.04	0.09 ± 0.02
	Verrucomicrobium (903)	0.00 ± 0.00	0.00 ± 0.00	0.00 ± 0.00	0.01 ± 0.01	0.01 ± 0.01	0.11 ± 0.04	0.13 ± 0.02
	Uncultured DEV007 (1517)	0.00 ± 0.00	0.00 ± 0.00	0.00 ± 0.00	0.00 ± 0.00	0.00 ± 0.00	0.00 ± 0.00	0.10 ± 0.03
Unclassified	Uncultured bacterium (315)	0.00 ± 0.00	0.00 ± 0.00	0.00 ± 0.00	0.02 ± 0.00	0.16 ± 0.01	0.00 ± 0.00	0.00 ± 0.00
	Uncultured bacterium (255)	0.00 ± 0.00	0.00 ± 0.00	0.00 ± 0.00	0.01 ± 0.00	0.06 ± 0.04	0.02 ± 0.02	0.24 ± 0.21
